# A concept of dual-responsive prodrugs based on oligomerization-controlled reactivity of ester groups: an improvement of cancer cells *versus* neutrophils selectivity of camptothecin†

**DOI:** 10.1039/d3md00609c

**Published:** 2024-01-31

**Authors:** Insa Klemt, Viktor Reshetnikov, Subrata Dutta, Galyna Bila, Rostyslav Bilyy, Itziar Cossío Cuartero, Andrés Hidalgo, Adrian Wünsche, Maximilian Böhm, Marit Wondrak, Leoni A. Kunz-Schughart, Rainer Tietze, Frank Beierlein, Petra Imhof, Sabrina Gensberger-Reigl, Monika Pischetsrieder, Marlies Körber, Tina Jost, Andriy Mokhir

**Affiliations:** a Department of Chemistry and Pharmacy, Organic Chemistry II, Friedrich-Alexander-University of Erlangen-Nürnberg (FAU) 91058 Erlangen Germany Andriy.Mokhir@fau.de; b Department of Histology, Cytology and Embryology, Danylo Halytsky Lviv National Medical University 79010 Lviv Ukraine; c Program of Cardiovascular Regeneration, Centro Nacional de Investigaciones Cardiovasculares Carlos III (CNIC) C. Melchor Fernández Almagro, 3 28029 Madrid Spain; d OncoRay, National Center for Radiation Research in Oncology, Faculty of Medicine and University Hospital Carl Gustav Carus, TU Dresden and Helmholtz-Zentrum Dresden-Rossendorf Dresden Germany; e National Center for Tumor Diseases (NCT) Partner Site Dresden Germany; f Department of Otorhinolaryngology, Head and Neck Surgery, Section of Experimental Oncology and Nanomedicine (SEON), FAU University Hospital 91054 Erlangen Germany; g Erlangen National High Performance Computing Center (NHR@FAU), FAU 91058 Erlangen Germany; h Computer-Chemistry-Center, Department of Chemistry and Pharmacy, FAU Germany; i Department of Chemistry and Pharmacy, Food Chemistry, FAU 91058 Erlangen Germany; j Department of Radiation Oncology, FAU University Hospital 91054 Erlangen Germany

## Abstract

Many known chemotherapeutic anticancer agents exhibit neutropenia as a dose-limiting side effect. In this paper we suggest a prodrug concept solving this problem for camptothecin (HO-cpt). The prodrug is programmed according to Boolean “AND” logic. In the absence of H_2_O_2_ (trigger T1), *e.g.* in the majority of normal cells, it exists as an inactive oligomer. In cancer cells and in primed neutrophils (high H_2_O_2_), the oligomer is disrupted forming intermediate (inactive) lipophilic cationic species. These are accumulated in mitochondria (Mit) of cancer cells, where they are activated by hydrolysis at mitochondrial pH 8 (trigger T2) with formation of camptothecin. In contrast, the intermediates remain stable in neutrophils lacking Mit and therefore a source of T2. In this paper we demonstrated a proof-of-concept. Our prodrug exhibits antitumor activity both *in vitro* and *in vivo*, but is not toxic to normal cell and neutrophils in contrast to known single trigger prodrugs and the parent drug HO-cpt.

## Abstract 2

We introduced a concept of prodrugs activated by H_2_O_2_ and mitochondrial OH^−^, which is applicable to camptothecin and its derivatives.

## Graphical abstract

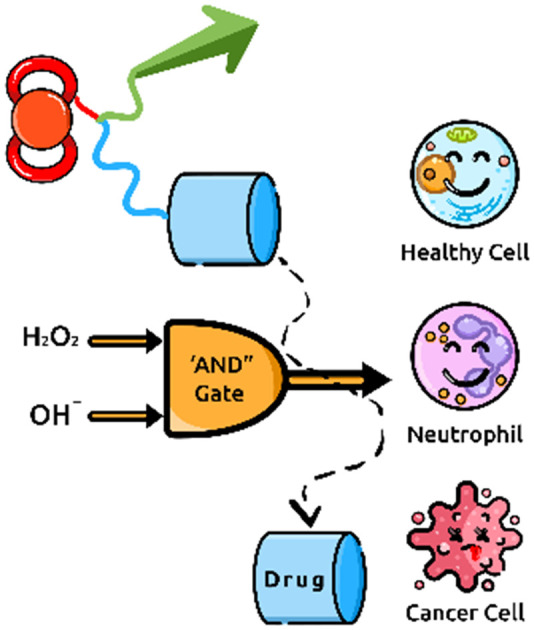

## Introduction

Chemotherapy is one of the three major concepts in current cancer treatment alone, or in combination with surgery and radiotherapy. Clinically used chemotherapeutics include both natural and synthetic compounds, *e.g.*, camptothecins,^1^ bleomycin,^2^ doxorubicin,^3^ paclitaxel^4^ and Pt(ii) complexes (cisplatin, oxaliplatin).^5^ Their common side effect is neutropenia,^1–5^ a condition characterized by the low number of neutrophils. Since neutrophils belong to the first line of defence against infections, neutropenia causes strong suppression of the immune system in patients and, as a consequence, even minor infections can become life threatening.

Side effects of drugs can be eliminated by converting them to prodrugs, which are activated under cancer specific conditions, but remain inactive in other cells.^6^ For example, we^7^ and others^8^ have used differences in the amount of H_2_O_2_ in cancer and normal cells^9^ to design H_2_O_2_-responsive anticancer prodrugs. However, neutrophils also produce large amounts of H_2_O_2_ that can activate the prodrugs causing neutropenia. Representative examples include H_2_O_2_-responsive prodrugs of (a) gemcitabine exhibiting residual neutrophil toxicity compared to the vehicle^10^ and (b) *N*-alkylaminoferrocene (AF) causing death of neutrophils *via* formation of neutrophil extracellular traps (NETs).^11^ These literature data indicate that improved concepts for H_2_O_2_-responsive prodrugs are warranted.

**Fig. 1:**
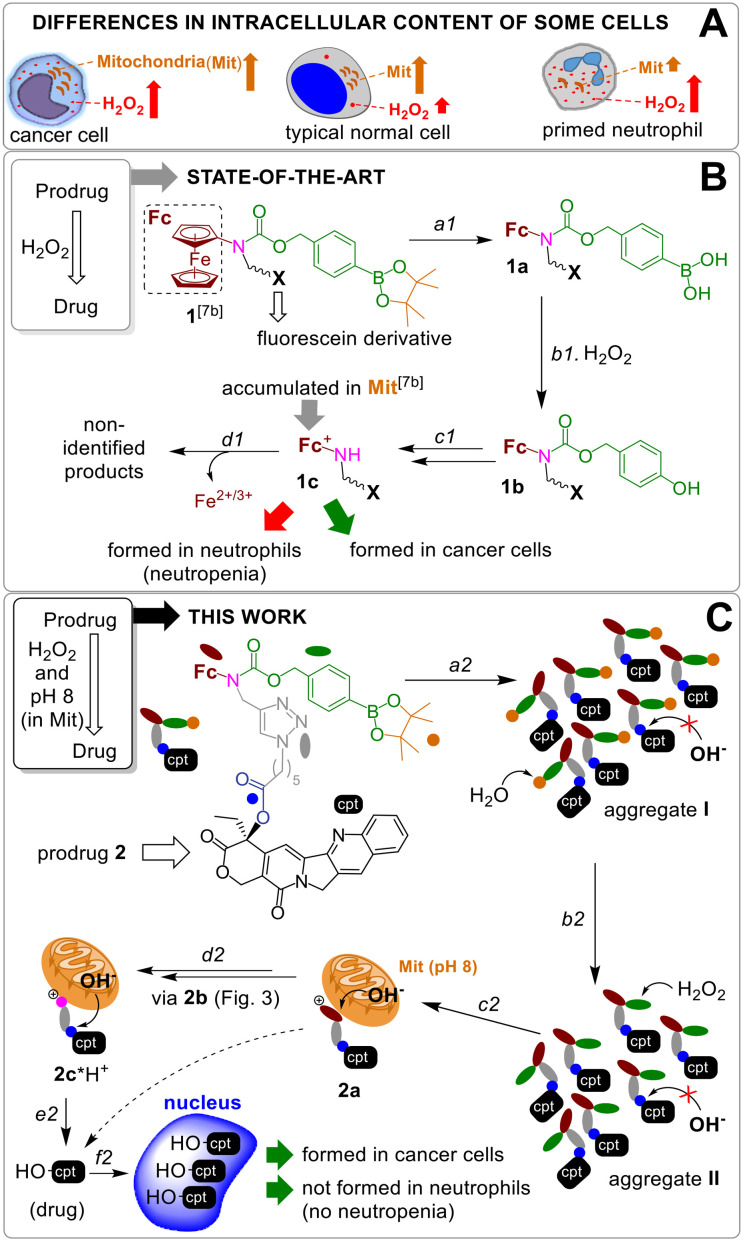
A: Differences in H_2_O_2_ amount and the number of Mit in human cells. B: The activation of known prodrug 1 by H_2_O_2_: a1 – hydrolysis of pinacol ester; b1 – H_2_O_2_-mediated cleavage of arylboronic acid; c1 – 1,6-elimination of *p*-quinone methide, CO_2_ release and fc oxidation by H_2_O_2_; d1 – decomposition of ferrocenium 1c. C: Activation of prodrug 2: a2 – aggregation of the prodrug in aqueous solution; b2 = a1; c2 = b1 + c1; d2 = d1; e2 – hydrolysis of ester E in Mit at pH 8; f2 – accumulation of HO-cpt in nucleus.

**Fig. 2:**
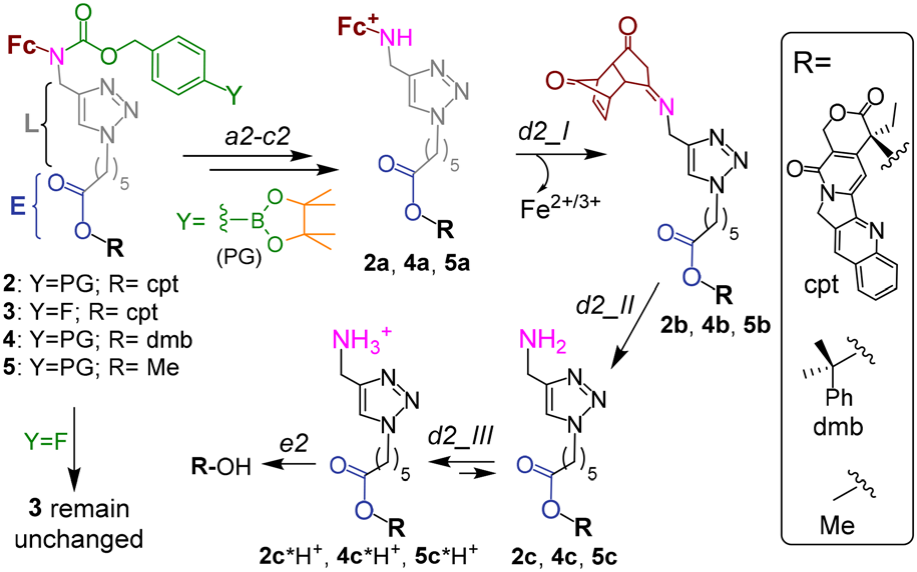
The mechanism of activation of prodrug 2, its analogues 4 and 6 as well as unreactive controls 3 and 5. Steps a2–c2 and e2 are described in the caption to Fig. 1C. Steps d2_I and d2_II occur spontaneously.

Since the number of mitochondria (Mit) in cancer cells is substantially higher than that in neutrophils (Fig. 1A),^12^ we hypothesized that a combination of the H_2_O_2_-mediated activation with the Mit-driven chemistry can be used to improve cancer cells *versus* neutrophils specificity of H_2_O_2_-responsive prodrugs. Herein, we report on the data confirming the feasibility of this hypothesis. We developed AF^PG2/PG1^-L-E-cpt (where AF: aminoferrocene, PG2/PG1: a H_2_O_2_-responsive protecting group, L: a linker, E: an ester moiety, cpt: a camptothecin residue, Fig. 1 and 2), which is a prodrug of an inhibitor of topoisomerase I (TOPI) camptothecin (HO-cpt). HO-cpt was selected, since along with the strong anticancer activity it exhibits severe neutropenia.^1^ Two HO-cpt derivatives (irinotecan and topotecan) are already used in clinics.^1^ Single trigger H_2_O_2_ – activated prodrugs of HO-cpt^13^ as well as single trigger mitochondrial OH^−^ (OH^−^_Mit_, pH 8)^14^ – activated prodrugs have been previously described.^15^ In contrast, the reported here AF^PG2/PG1^-L-E-cpt is a dual trigger prodrug activated only when H_2_O_2_ (T1) and a OH^−^_Mit_ (T2) are present (Fig. 1B and C). It exhibits low μM anticancer activity *in vitro* towards a variety of human cancer cell lines representing blood, ovarian, prostate, pharynx and tongue, but is not toxic towards normal cells including PMA-primed neutrophils. Its anticancer activity is potentiated by ionizing radiation. The prodrug retains its activity *in vivo* in Nemeth–Kellner lymphoma model of murine cancer. In contrast to HO-cpt, it does not induce neutropenia *in vivo*.

## Results and discussion

A previously reported prodrug 1 is hydrolyzed within <2 h in aqueous solution with formation of the boronic acid 1a (step a1, Fig. 1B).^7*b*^ The 1a is taken up by both normal and cancer cells, but is activated only in H_2_O_2_-rich, cancer cells *via* steps b1–c1 with formation of a lipophilic cation ferrocenium 1c (*via* intermediate 1b), which is found to be accumulated in Mit.^7*b*^ The 1c is decomposed forming not identified polar products. The T1 (H_2_O_2_)-responsive moiety of the 1 was applied to design the prodrug reported in this paper.

### Design of a T1/T2-responsive prodrug 2

The proposed activation mechanism of prodrug 2 and its chemical structure are shown in Fig. 1C and 2.

It includes three elements: moieties responsive to T1 (arylboronic acid pinacol ester, coloured green in Fig. 2 (ref. 7*b*)) and T2 (L-E) as well as a drug fragment (cpt), deactivated by acylation of the critical for the activity HO group with formation of an ester E.^16^ Ideally, the E is cleaved in the presence of both H_2_O_2_ (T1) and OH^−^_Mit_ (T2) releasing the active drug HO-cpt (cancer cell: T1^+^/T2^+^), but remains intact when one of the triggers is absent (normal cell: T1^−^/T2^+^; neutrophil: T1^+^/T2^−^). This goal is achieved in the following way. The prodrug is designed to be lipophilic enough to exist in the oligomer-form in aqueous solution (a mixture of aggregates I and II, Fig. 1C). In the aggregates the hydrophobic L-E moiety is buried in the interior and, therefore, not accessible to the hydrolysis by hydrophilic T2. A polar boronic acid moiety is located at the exterior of the aggregate and accessible to T1. Assuming that the T1-responsive moiety^7*b*^ retains its properties in the prodrug, its activation induced by T1 will occur as outlined in Fig. 2. In steps b2 and c2, the prodrug will be converted to ferrocenium 2a in the reaction with T1. A positive charge of this intermediate should destabilize the aggregate, thereby favouring the monomeric species. In cells, the de-aggregation will be further supported by loading of the 2a into Mit (Fig. 1C). In this state the ester E will be accessible for T2 and can therefore be hydrolysed forming HO-cpt as indicated with a dashed arrow in Fig. 1C. As it is described in the experimental part, the latter reaction occurs stepwise (d2 + e2) *via* intermediates 2b, 2c and 2c*H^+^ (Fig. 2). In step f2, the drug is accumulated in the nucleus, where it inhibits TOPI.

### Activation of the prodrug by T1 and T2 in cell free settings

Synthesis of the prodrug and controls is described in the ESI.† Their purity was confirmed by C, H, N elemental analysis and was found to be >95%. The characterization and confirmation of prodrug stability in solid state and solution are provided in Fig. S1–S24.†

### Aggregation of the prodrug

**Fig. 3:**
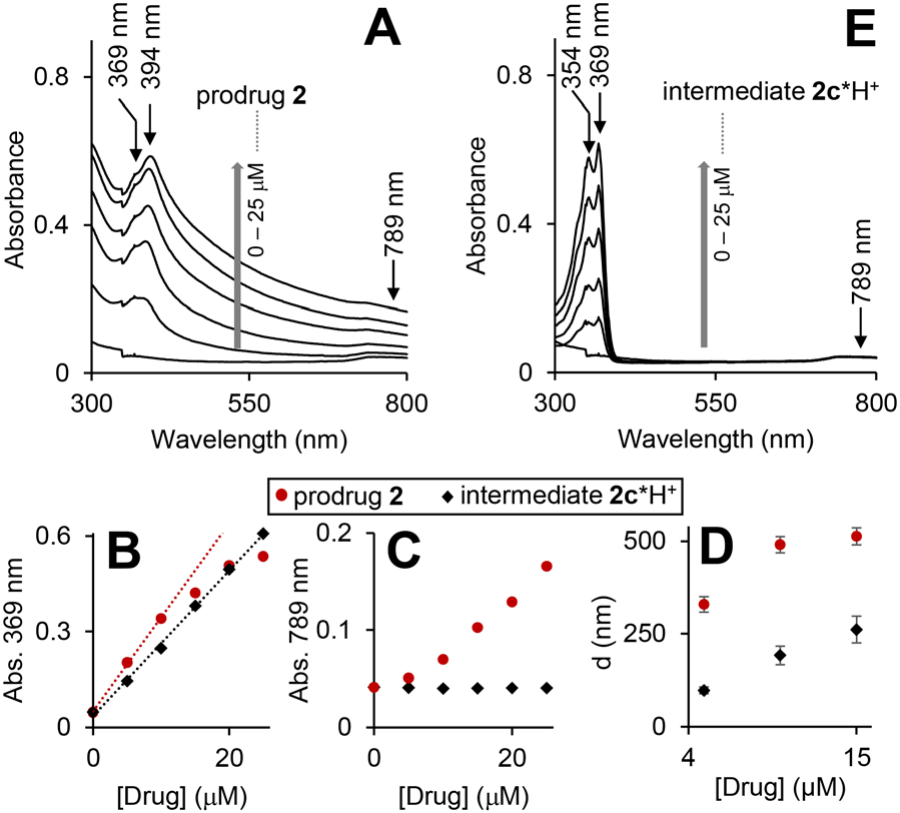
UV-visible spectra of the prodrug 2 (A) and intermediate 2c*H^+^ (E) in triethylammonium acetate (TEAA) buffer (150 mM, pH 8, CH_3_CN 15% v/v, DMSO 1%, v/v) at concentrations 0, 10, 15, 20 and 25 μM. Plots of absorbance at 369 and 789 nm *versus* time obtained form the spectra shown in A and E are provided in plots B and C, correspondingly. Linear fits of the initial time periods (0–10 min) are provided as dotted lines. D: DLS data for aqueous solutions (same as in A and E) of the prodrug (red circles) and the intermediate (black diamonds): “D” is the mean size of the aggregates detected.

The prodrug 2 is lipophilic as evidenced by its high *n*-octanol/water partition coefficient (log *P*) of 4.27 ± 0.04 (Table S1, ESI†). However, it is still moderately soluble at ≤30 μM in Dulbecco's phosphate-buffered saline (DPBS) (Table S2, ESI†). To investigate the aggregation phenomena in the aqueous solutions, we recorded UV-visible spectra of the prodrug at concentrations up to 25 μM (Fig. 3A).

As expected, the spectra are dominated by the cpt chromophore (*λ*_max_ = 369 and 394 nm), whereas the ferrocenyl moiety is not visible due to its low extinction coefficient. Absorbance at these maxima does not obey Beer–Lambert law (Fig. 3B: representative data for *λ*_max_ = 369 nm) indicating the presence of more than one cpt-chromophore-containing species in solution. Interestingly, the significant absorbance at >500 nm is also observed (Fig. 3A and D). Since the prodrug has no chromophores absorbing >500 nm, this feature can be interpreted by Rayleigh light scattering on an aggregate. By using dynamic light scattering (DLS) we confirmed the formation of aggregates with sizes ranging from 350 ± 21 to 535 ± 65 nm. The *λ*_max_ values of the prodrug (369/394 nm) are close to those observed for the solution of HO-cpt dissolved in unpolar solvent toluene (369/386 nm), but do not match those of HO-cpt dissolved in polar water (355/368 nm).^17^ These data indicate that the cpt moiety is located in the hydrophobic interior of the aggregates. Since the E-L is directly linked to the cpt, it will also be located in the hydrophobic interior that will protect it from the attack by T2 (Fig. 1C).

### Reaction of the prodrug with H_2_O_2_

**Fig. 4:**
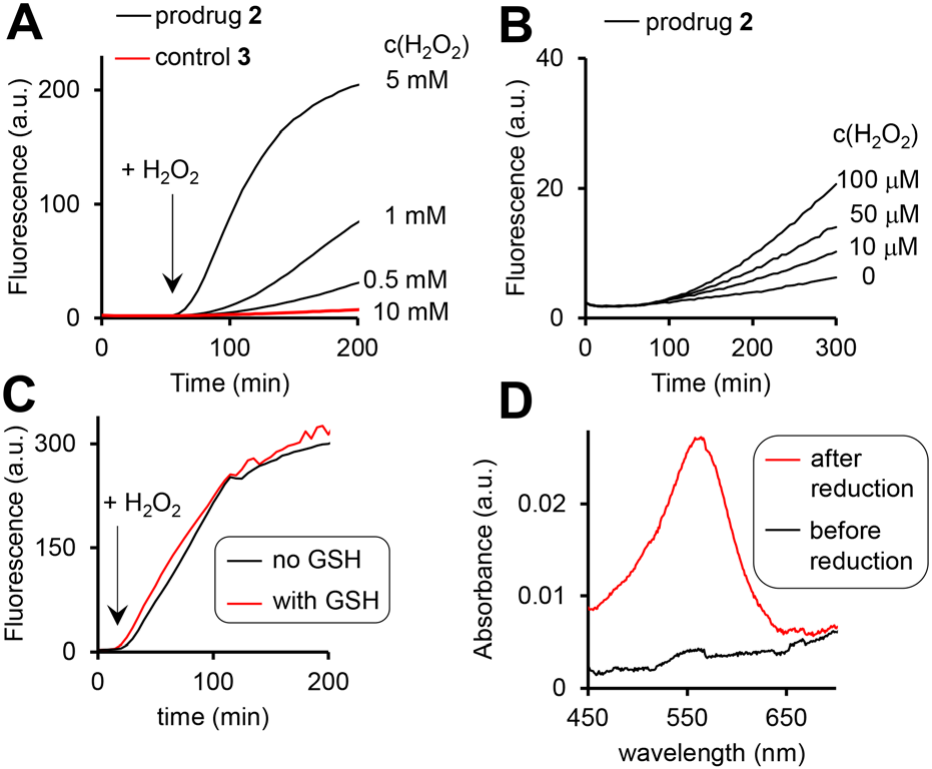
The fluorescence increase (*λ*_ex_ = 365 nm, *λ*_em_ = 400 nm) in aqueous solutions of the prodrug 2 (10 μM, pH 8) in the presence of H_2_O_2_ (A: 0.5–10 mM; B: 0–100 μM). C: Data indicating that GSH (5 mM) does not affect the activation of the prodrug by H_2_O_2_ (10 mM). D: Detection of Fe^2+^ (“before reduction”) and the sum of Fe^3+^ and Fe^2+^ (“after reduction”) in the solution of the prodrug (20 μM, pH 8) incubated with H_2_O_2_ (10 mM) for 2 h. Other experimental details are provided in the ESI.†

The prodrug 2 as well as the 3, where the H_2_O_2_-responsive boronic acid moiety was replaced with a fluorine, are practically not fluorescent in aqueous solutions (Fig. 4A). The possible reasons are (a) photo-induced electron transfer (PET) from the Fc moiety to the excited state of the cpt and (b) aggregation. We observed the H_2_O_2_-dose dependent increase of the fluorescence characteristic for the cpt fluorophore (*λ*_ex_ = 365 nm, *λ*_em_ = 460 nm) in the mixtures of prodrug (20 μM) and H_2_O_2_ (10 μM to 10 mM, Fig. 4A and B). Further data obtained at pH 6, 7, 8 and 10 are shown in Fig. S26A, ESI.† This reaction is facilitated at higher pH (1.5- and 1.9-fold faster at pH 8 and 10 than at pH 7) and substantially slowed down at the acidic pH (Table S3, ESI†). Importantly, the prodrug activation by H_2_O_2_ is not affected by the physiological concentration of glutathione (GSH, 5 mM) (Fig. 4C) and is ∼2-fold activated by fetal bovine serum (FBS) (5%) (Fig. S26C, ESI†) indicating that the reaction is possible within living cells.

**Fig. 5:**
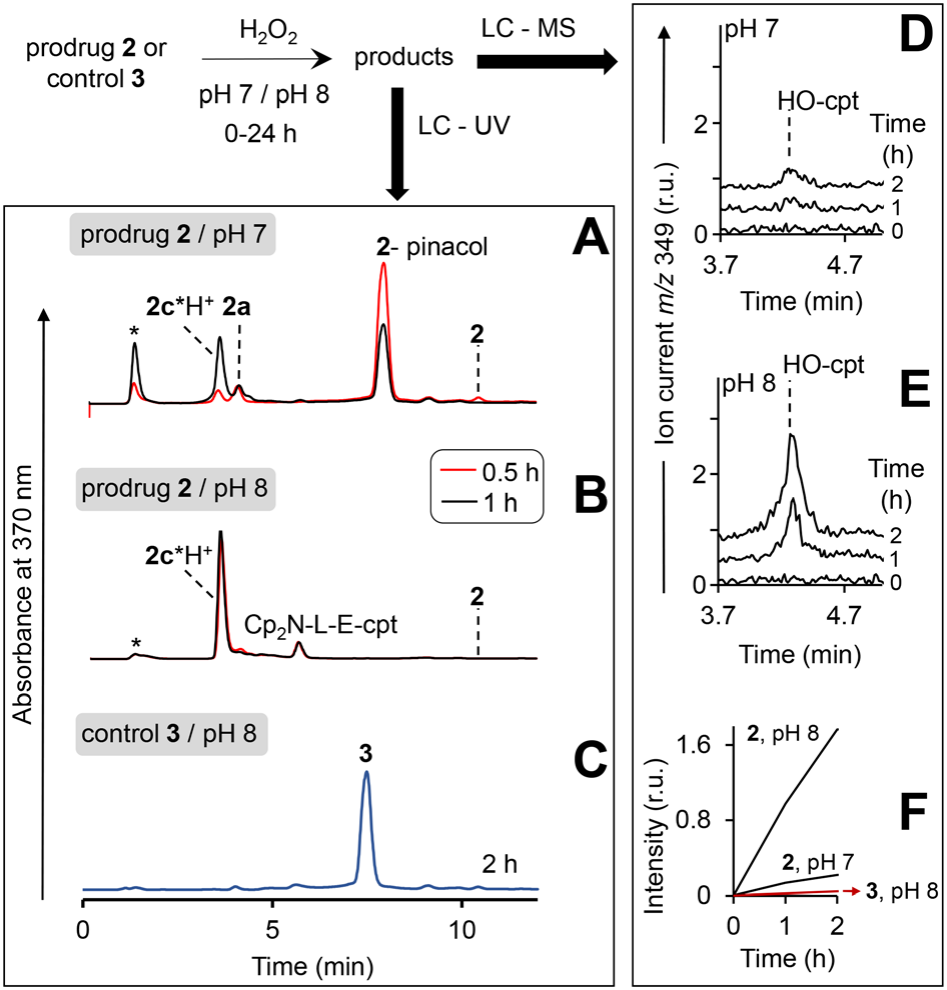
Study of the reaction of the prodrug 2 and the control 3 with H_2_O_2_ at pH 7 and 8 by using HPLC. HPLC profiles (detection of absorbance at 370 nm) are shown in A and B. Solutions of the prodrug (20 μM) at pH 7 (A) or pH 8 (B) were incubated for 0.5–1 h in the presence of H_2_O_2_ (10 mM) and analysed. Injection peak is indicated with *. C: The same as in B, except that the prodrug was replaced with the control 3 and the incubation time was extended to 2 h. D and E: Analysis of the prodrug/H_2_O_2_ mixtures (incubation times are shown on the plot) by using LC – quadrupole-time-of-flight mass detector (detection of positive ion current at *m*/*z* 349 Da: protonated HO-cpt). F: Dependence of peak areas at *m*/*z* 349 observed in experiments shown in D and E from the time of incubation with H_2_O_2_.

Furthermore, the activation relies on the presence of the arylboronic acid group, since the control 3 lacking this group remains non-emissive even in the presence of the highest H_2_O_2_ concentration tested (10 mM, Fig. 4A, red colored trace). Importantly, both the prodrug 2 and the control 3 are stable at pH 7 and 8 in the absence of H_2_O_2_, indicating that the direct hydrolysis of the ester E does not take place. Compared to pH 7, the direct hydrolysis is facilitated at pH 10 by 6-fold for the prodrug and 2.5-fold for the control (Table S3, ESI†). The fluorescent product generated upon the prodrug activation can be both HO-cpt and other fluorescent intermediates as will be discussed later. To identify these products, we first examined the release of Fe ions in the mixture of the prodrug (20 μM) and H_2_O_2_ (10 mM) by making use of the formation of red colored complex of Fe^2+^ with ferrozine.^18^ We detected no Fe^2+^ both at pH 7 and 8 after 2 h incubation (ESI†). However, when the mixtures were first reduced by hydroxylamine to convert Fe^3+^ to Fe^2+^, followed by addition of ferrozine, the characteristic red solution was obtained at both pH 7 and 8: representative data obtained at pH 8 are shown in Fig. 4D. This indicates the H_2_O_2_-mediated release of Fe^3+^, occurring in the result of the decomposition of the intermediate 2a (Fig. 2). Other intermediates and HO-cpt were identified by using HPLC coupled to UV-light (LC-UV) and electrospray ionization MS detectors (LC-MS, Fig. 5).

In particular, at pH 7 traces of the prodrug are observed at the retention time *R*_*t*_ = 10.5 min (*m*/*z* 986 [M–e^−^]^+^) after 30 min incubation with H_2_O_2_ (10 mM) (red line, Fig. 5A). After 1 h incubation it is not detectable anymore (black line). The major peak at both 30 min and 1 h incubation at pH 7 is the hydrolyzed prodrug, which has lost a pinacol moiety – 2-pinacol: *R*_*t*_ = 7.8 min (*m*/*z* 904 [M–e^−^]^+^). Further detected intermediates include a 2a (*R*_*t*_ = 4.1 min, *m*/*z* 726 [M]^+^) and its decomposition product 2c*H^+^ (*R*_*t*_ = 3.7 min, *m*/*z* 543 [M]^+^). The formation of the latter intermediate is in agreement with the release of Fe^3+^ (Fig. 4D). At 30 min incubation ferrocenium/amine ratio is equal to 3/4, whereas at 1 h incubation it is 1/9. At pH 8 the same intermediates are formed. However, the reaction is substantially faster. Already after 30 min, neither prodrug nor its hydrolyzed form are detectable, whereas the ferrocenium intermediate is present only as traces. The major product is 2c*H^+^. Additionally, a weak peak is observed at *R*_*t*_ = 5.6 min (*m*/*z* 701 [M]^+^), which corresponds to 2b formed in the result of decomposition of 2a (Fig. S27, ESI†).

We observed that at the separation conditions selected for LC-UV (Fig. 5A and B) both HO-cpt and 2c*H^+^ (Fig. S28, ESI†) elute at the same time (3.7 min) that did not allow for the accurate quantification of HO-cpt. We solved this problem by using more efficient chromatographic separation with high-resolution mass spectrometry (Fig. S29, ESI†). The data obtained are provided in Fig. 5D and E. In Fig. 5F, areas of the peaks corresponding to HO-cpt are plotted as a function of time. In the prodrug/H_2_O_2_ mixture at pH 7 a HO-cpt peak is observed only after 2 h incubation (Fig. 5D). In contrast, at pH 8 this peak is detectable already after 1 h and the peak intensity is increased after 2 h. Importantly, practically no HO-cpt could be detected in the mixture of the control 3/H_2_O_2_ at pH 8 (red trace, Fig. 5F) indicating that the direct hydrolysis of E does not take place.

To investigate whether the latter reaction sequence is specific for cpt derivatives, we prepared a prodrug 4 containing 1,1-dimethylbenzyl in place of the cpt moiety. By using LC-UV-MS we confirmed that this compound forms related intermediates as the prodrug 2 in the presence of H_2_O_2_ (Fig. S30A, ESI†). The reaction was also found to be facilitated at pH 8 as compared to pH 7. By using GC-MS we confirmed the formation of HO-dmb in the 4/H_2_O_2_/pH 8 mixture (Fig. S30B, ESI†). Thus, these data indicate that the reaction sequence reported in this paper is not restricted to the cpt-prodrugs. It can potentially be applied to design prodrugs of other anticancer drugs, which possess a tertiary OH group critical for their activity. Possible examples of such drugs are clinically used irinotecan and topotecan.^1^

### The mechanism of the reaction of the prodrug with H_2_O_2_ at pH 8

According to the data discussed above the prodrug is aggregated in aqueous solution that blocks the reactivity of the L-E (Fig. 3A and D). In the presence of H_2_O_2_ the prodrug is converted to the intermediate 2c*H^+^ (Fig. 5A and B). This compound is monomeric according to the following experimental evidences. First, its UV-visible spectra at 0 to 25 μM are characteristic for a monomeric cpt derivative. In particular, the absorbance maxima (354/369 nm) are close to those observed for HO-cpt dissolved water (355/368 nm).^17^ Absorbance at these maxima obey Beer–Lambert law (Fig. 3B, black trace). No absorbance is observed at >500 nm, indicating the absence of aggregates able to scatter light. Finally, DLS data indicate the absence of aggregates larger than 100 nm at the compound concentration <5 μM. This indicates that the cpt in 2c*H^+^ is exposed to the solvent.

To find out whether the hydrolysis is facilitated by the intramolecular interactions, we analysed possible conformations of 2c*H^+^ in aqueous solution by molecular dynamics (MD) simulations (Fig. S31A–F, ESI†). We observed only low probabilities for conformations where hydrogen bonding between the ammonia and the C=O (E) is possible (Fig. S31A–C, ESI†). Combined quantum mechanical and molecular mechanical (QM/MM) simulations of the hydrolysis reaction by an OH^−^ ion on 2c*H^+^ In a stretched conformation, *i.e.* with the ammonium group far from the E, show a step-wise reaction, in which the rate-determining step is the OH^−^ attack with formation of a tetrahedral intermediate with a feasible free energy barrier of 34.0 ± 0.7 kcal mol^−1^ (Fig. S31E and F, ESI†). In simulations, in which the ammonium group has been constrained to be close to the C=O (E), the free energy barrier for the nucleophilic attack does not change significantly (36.1 ± 0.6 kcal mol^−1^) compared to the simulations without this distance constraint (Fig. S31F†). This indicates that hydrolysis of E does not rely on intramolecular interactions involving the ammonium group. It is therefore conceivable that the hydrolysis occurs by direct OH^−^ attack of the E of 2c*H^+^. Further experimental data are necessary to confirm or reject this suggestion.

### Effect of the prodrug on viability of cancer and normal cells

We selected several representative human cancer cell lines from different organs, including Burkitt's lymphoma BL-2 (blood), A2780 (ovary), DU-145 (prostate), SAS (tongue) and FaDu (pharynx), known to produce elevated ROS amounts.^7,19,20^ Incubation of these cells with the prodrug for 96 h affects strongly their viability. In particular, half inhibitory concentration (IC_50_) ranges from 0.3 ± 0.1 μM (for most sensitive BL-2 cells) to 1.1 ± 0.5 μM (for least sensitive DU-145 cells) (Table 1).

**Table 1:** Effect of the prodrug/controls on viability of selected cancer cell lines

Prodrug/controls	Cancer cell lines/IC_50_, μM				
	BL-2^a^	A2780^a^	DU-145^a^	SAS^b^	FaDu^b^
2	0.3 ± 0.1	1.0 ± 0.2	1.1 ± 0.5	4 ± 2	8 ± 2
3	1.3 ± 0.2	8.0 ± 0.2	>30	>40	>40
5	28 ± 5	34 ± 5	>50	—	—
HO-cpt	0.02 ± 0.01	0.08 ± 0.02	0.03 ± 0.01	—	—

a 96 h incubation.

b 48 h incubation.

Experiments with controls revealed the importance of the individual components of the prodrug. In particular, the control 5 (ref. 7*b*) lacking the cpt fragment is from 34- to 93-fold and the control 3 lacking the H_2_O_2_-responsive fragment is from 4.3 to 27.5-fold less active than the prodrug (Table 1). The trend is retained at the shorter incubation times of 24 and 48 h (Tables S5 and S6, ESI†). Importantly, the prodrug does not affect the viability of representative normal cell lines, producing low amounts of intracellular ROS: normal human dermal fibroblasts (NHDF), IC_50_ > 20 μM; retinal pigment epithelia (ARPE-19) cells, IC_50_ > 10 μM and primary human fibroblasts (HF), IC_50_ > 10 μM (Table S6, Fig. S32, ESI†). The differences in the anticancer effects of the prodrug and the controls can be caused by the different uptake efficacy. To evaluate this possibility, we studied the uptake of the latter compounds in representative cancer cells – BL-2 (Tables S8 and S9 ESI†). We found that the uptake of control 3 is more efficient than that of the prodrug 2, whereas control 5 (ref. 7*b*) and the prodrug 2 are taken up with almost the same efficacy. Thus, the stronger anticancer effect of the prodrug than that of the controls is not caused by the differences in their uptake efficacy.

### Enhancement of the prodrug efficacy

The anticancer effect of the prodrug 2 is 13 to 37-fold weaker than that of HO-cpt (Table 1) suggesting that its intracellular activation is not complete. Since ionizing irradiation (IR) increases ROS in cells,^21^ we assumed that it could also facilitate the prodrug activation.^22^ We tested this hypothesis in two representative cell lines, SAS and FaDu, which are cancer models established in our laboratory for exploring synergistic effects between IR and new drugs.^22^

First, we confirmed that the prodrug inhibits growth of both SAS (IC_50_ = 4 ± 2 μM) and FaDu cells (IC_50_ = 8 ± 2 μM) at 48 h incubation time. At the concentration of 0.5 μM (but not 0.2 μM) it also inhibits the capacity of these cells to form colonies (*p* < 0.01). Next, we investigated inhibition of colony formation capacity of SAS and FaDu cells by the combination of the prodrug and IR. The prodrug at non-toxic (0.2 μM) and moderately toxic (0.5 μM) concentrations and IR at doses ranging from 0 to 10 Gy were used. For the highest IR dose of 10 Gy and one representative cell line (FaDu) the IR-induced increase of the intracellular ROS level was confirmed (Fig. S33, ESI†). We were pleased to observe that the effect of the prodrug at both tested concentrations on both cell lines is strongly enhanced by IR (*p* < 10^−3^) (Fig. S34, ESI†). In contrast, the H_2_O_2_-resistant control FcN^F^-L-E-cpt exhibits practically no synergy with IR (Fig. S34, ESI†).

### A mechanism of anticancer activity of the prodrug

**Fig. 6:**
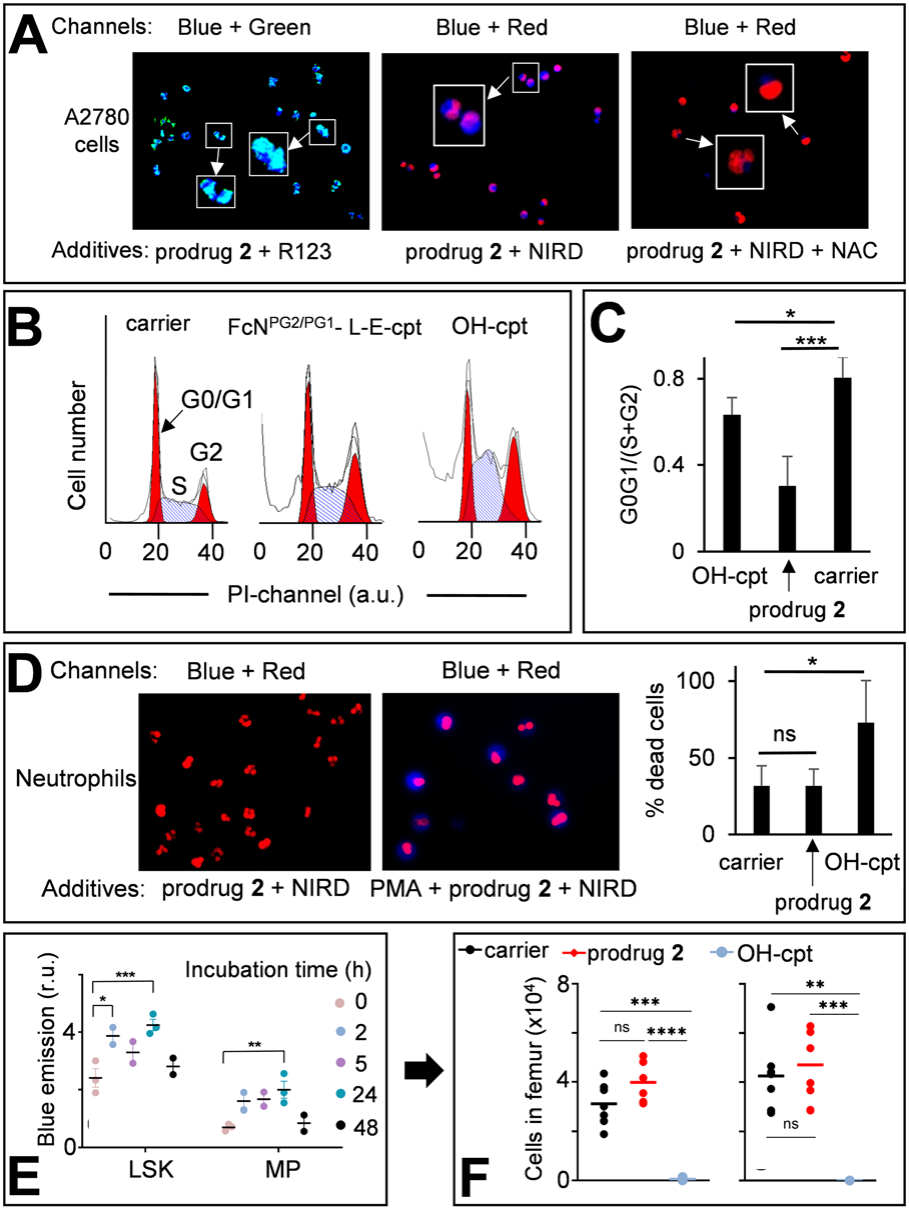
A: Left image – A2780 cells incubated with the prodrug 2 (20 μM) and Mit-specific dye R123 (1 μM). Two channels were used for emission monitoring: Ch1 – *λ*_ex_ = 365 nm, *λ*_em_ = 420–470 nm (for detecting PDFPs) and Ch2 – *λ*_ex_ = 430–510 nm, *λ*_em_ = 475–575 nm (for detecting R123). Ch1 + Ch2 combination is provided. Middle image – A2780 cells incubated with the prodrug 2 (20 μM) and nuclei-labelling NUCLEAR-ID® red dye (NIRD). Two channels were used for emission monitoring: Ch1 and Ch3 – *λ*_ex_ = 538–562 nm, *λ*_em_ = 570–640 nm (for NIRD). Ch1 + Ch3 combination is shown. Right image: The same as the middle image, except that *N*-acetyl cysteine (NAC, 2 mM) was added. Zoomed-in (×2.5) images are shown in white boxes. B: Cell cycle distribution of A2780 cells treated for 72 h either with DMSO (1%, v/v, carrier) or HO-cpt (5 nM) or the prodrug 2 (0.5 μM). C: Quantification of the data shown in B. G0G1/(S + G2) – the number of cells in phases G0 and G1 divided by the number of cells in phases S and G2. D: The prodrug 2 (20 μM) and NIRD incubated either with human neutrophils (left image) or human neutrophils pre-incubated with PMA (right image). Combined “Ch1 + Ch2” images are shown (Ch1 and Ch2 are defined in in inset A). Right plot – % dead cells in human neutrophils incubated with the carrier, the prodrug 2 and HO-cpt (both 20 μM) for 2 h. E: Monitoring the fluorescence characteristic for PDFPs in immature cells (lineageNEG Sca-1+ c-kit+ (LSK) and myeloid progenitor (MP) cells) from BM in mice injected i.p. on days 0, 2 and 4 with the prodrug (12 mg kg^−1^). F: Monitoring of the number of LSK and MP cells in BM of mice treated as described in E. The analysis was conducted on day 7. Paired *t*-test was conducted between days 0 and 7. *: *P* < 0.05; **: *P* < 0.01; ***: *P* < 0.001.

The prodrug 2 is not fluorescent (Fig. 4A). When it reacts with H_2_O_2_ two long- (2c*H^+^ and HO-cpt) and one short-living (2a) fluorescent products are formed, each exhibiting a broad emission in the range of 420–470 nm (Fig. 4A and 5A, B, D and E). Further, we will call a mixture of these compounds prodrug-derived fluorescent products (PDFPs). Intermediates 2a and 2c*H^+^ are inactive cpt esters unable to bind TOPI, whereas HO-cpt is a TOPI inhibitor and a highly potent anticancer drug. Thus, the fluorescence increase in the reaction of the prodrug 2 with H_2_O_2_ will not necessarily correlate with the high anticancer activity, but is rather an indication of the first step of the activation. We investigated formation and intracellular localization of PDFPs (blue channel) in representative cancer A2780 cells. Mit was labelled with rhodamine 123 (R123: green channel) and nuclei – with NUCLEAR-ID® Red dye (NIRD: red channel). As shown in Fig. 6A, the blue signal is clustered in particular regions. This signal is co-localized with both R123 (green, Pearson's coefficient *r* = 0.68) and NIRD (red, *r* = 0.81) confirming that the PDFPs are present in Mit and in nuclei of the cells. We observed that the signal of PDFPs is practically absent in A2780 cells pretreated with ROS scavenger *N*-acetylcysteine (NAC, Fig. 6A, right image) indicating that PDFPs are formed in cells in the ROS-dependent reaction. This data are in agreement with the suggested mechanism (Fig. 1C) including formation of 2a in the cytoplasm, its accumulation in Mit, where it is transformed *via* the sequence of the OH^−^_Mit_-mediated reactions to HO-cpt. The drug then migrates to the nucleus, where it is trapped by binding to its target TOPI.^23^

Next, we investigated whether the prodrug acts as a cpt-derivative releasing HO-cpt in cells or as a typical AF-prodrug. HO-cpt affects intracellular ROS levels weakly, but is known to induce a strong cell cycle arrest in S and G2 phases as reflected, amongst others, in altered static DNA histograms (Fig. 6B and C). In contrast, the typical effect of AF prodrugs is amplification of intracellular ROS (*e.g.* control 5,^7*b*^ Fig. S37 and S38, ESI†), whereas no major effect on the cell cycle distribution – rather a slight increase in the G1 phase cell fraction – is observed.^7,19^ The prodrug practically does not facilitate ROS production (Fig. S37–S39, ESI†), but causes changes of cell cycle distribution characteristic for HO-cpt (Fig. 6B and C). It is therefore likely that HO-cpt is intracellularly released from the prodrug.

### Effects of the prodrug on neutrophils

We investigated the activation of the prodrug with formation of PDFPs in human neutrophils from healthy individuals. Nuclei of the neutrophils were counter-stained with NIRD (red channel). As shown in Fig. 6D (left image), the blue signal is absent in the cells incubated with the prodrug 2 for 2 h indicating that the latter compound remains intact. In contrast, neutrophils primed with phorbol myristate acetate (PMA, 50 nM), inducing H_2_O_2_ generation, feature the evenly distributed, intense blue signal indicating PDFPs formation (Fig. 6D, right image). However, HO-cpt does not seem to be formed under these conditions, since the prodrug 2 does not enhance the PMA toxicity of neutrophils (Fig. 6D, right plot). In contrast, HO-cpt is toxic under the same conditions.

Next, we assessed the characteristic for PDFPs fluorescence in neutrophils of blood and bone marrow (BM) as well as in immature BM cells (LineageNEG Sca-1+ c-Kit+ (LSK) and myeloid progenitor (MP) cells) from mice treated with the prodrug. At the early incubation time of 2 h, we observed some minor (*p* < 0.05) increase of the fluorescence of blood neutrophils (Fig. S40A, ESI†). The more pronounced fluorescence increase was observed in LSK (2/24 h incubation) and MP cells (24 h) (Fig. 6E). These data indicate formation of PDFPs *in vivo*. However, this does not correlate with a decrease of the number of the corresponding cells in blood and BM (Fig. 6F, S40B and C, ESI†), thereby allowing concluding that toxic drug HO-cpt is not formed at these conditions. Thus, the *in vivo* data are also in agreement with the suggested mechanism of action of the prodrug (Fig. 1C).

### Antitumor activity of the prodrug *in vivo*

**Fig. 7:**
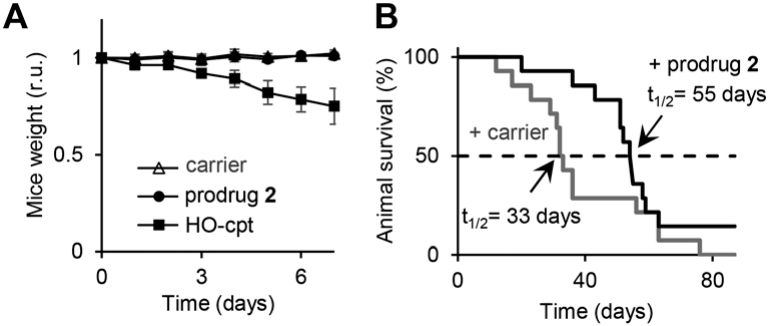
A: Change of weight of wild type mice treated either with DMSO only (carrier: “-”) or the prodrug 2 (12 mg kg^−1^) or drug HO-cpt (6 mg kg^−1^), which were injected i.p. on days 0, 2 and 4. B: Effects of the prodrug (12 mg kg^−1^) and the carrier injected i.p. on days 1, 3, 6, 8, 10, 13, 15, 17, 20 and 22 on survival of mice carrying Nemeth–Kellner lymphoma (Kaplan–Meier plot). The prodrug prolongs mice survival from 33 to 55 days (*p* < 0.05).

Encouraged by the excellent *in vitro* properties of the prodrug 2, we studied its toxicity and antitumor activity *in vivo*. We observed that at doses of 3 to 24 mg kg^−1^ (interperitoneal (i.p.) injections on days 0, 2 and 4), it does not affect weight of Balb/c mice in a 7-day experiment (Fig. S41, Table S10, ESI†). On day 7, the animals were sacrificed and their internal organs inspected. We detected no apparent damage (Fig. S42, ESI†). Further, we extracted the prodrug 2 and PDFPs from the organs and measured the fluorescence of the extracts at *λ*_ex_ = 365 nm, *λ*_em_ = 450 nm (parameters characteristic for PDFPs). We observed no fluorescence increase between untreated and prodrug 2-treated groups (Fig. S43, ESI†). After the extracts were treated with H_2_O_2_ to activate the prodrug 2, significant signals could be detected in extracts from blood (*p* < 0.001), spleen (*p* < 0.001), adipose tissue (*p* < 0.01) and peritoneum (*p* < 0.01) (Fig. S44, ESI†). Thus, the prodrug 2 is accumulated in the mentioned organs, but remains there in the intact state. All these data indicate that at doses ≤24 mg kg^−1^, the prodrug 2 does not exhibit acute toxicity. In contrast, at 6 mg kg^−1^, HO-cpt exhibits acute toxicity reflected in its negative effect on weight of mice (Fig. 7A). At the dose of 24 mg kg^−1^ a precipitate of the prodrug 2 was detected on the surface of internal organs of peritoneal cavity and, locally, near the i.p. injection site. All further experiments were conducted at the highest tested dose, at which no precipitates of the prodrug were detected *in vivo*: 12 mg kg^−1^ (Fig. S42, ESI†). We studied the antitumor effect of the prodrug 2 in the Nemeth–Kellner (NK) lymphoma (Ly) NK/Ly model. The prodrug was injected i.p. on days 1, 3, 6, 8, 10, 13, 15, 17 and 20 at the dose of 12 mg kg^−1^. The control group received only the carrier (DMSO). We observed statistically significant (*p* < 0.05) prolongation of mice survival (*t*_1/2_) in the treated group to 55 days *versus* 33 days in the control group (Fig. 7B).

## Experimental

All experimental data are provided in the ESI.†

## Ethical statement

Black C57/BL6N mice and white mice Balb/c were bred at Animal house of Danylo Halytsky Lviv National Medical University (Lviv, Ukraine). The animal studies were approved by the local ethical committee (Permission to R. Bilyy; 20180226/P2 and 2014-2018/P6) and conducted according to the guidelines of the Federation of European Laboratory Animal Science Associations (FELASA).

## Conclusions

We developed the dual camptothecin prodrug, which is activated in cancer cells in the presence of H_2_O_2_ and OH^−^_Mit_. This mode of action relies on aggregation-derived blocking of the OH^−^_Mit_-sensitive ester E in the prodrug, which is released by de-aggregation in the presence of H_2_O_2_. The prodrug discriminates cancer cells from primed (H_2_O_2_-rich) neutrophils, since the latter cells have the low number of mitochondria and correspondingly lack the OH^−^_Mit_-trigger. We confirmed the effects in cell free settings. The prodrug exhibits high nM to low μM anticancer activity towards a variety of cancer cell lines derived from blood, ovary, prostate, tongue and pharynx *in vitro* and towards Nemeth–Kellner lymphoma *in vivo*, but does not induce neutropenia usual for camptothecins both *in vitro* and *in vivo*. We demonstrated that the prodrug concept reported in this paper is not restricted to the derivatives of cpt and can potentially be applied to other drugs containing the tertiary HO group critical for their activity.

In summary, our approach allows cancer cell-specific delivery of anticancer drugs. Other known approaches for cell specific drug delivery based on nanosized materials have been revewed.^24–26^

## Author contributions

Insa Klemt, Viktor Reshetnikov, Subrata Dutta, Galyna Bila, Itziar Cossío Cuartero, Adrian Wünsche, Maximilian Böhm, Marit Wondrak, Rainer Tietze, Frank Beierlein, Sabrina Gensberger-Reigl, Marlies Körber, Tina Jost: investigation, formal analysis, data curation, visualization, writing – original draft. Rostyslav Bilyy, Andrés Hidalgo, Leoni A. Kunz-Schughart, Rainer Tietze, Petra Imhof, Monika Pischetsrieder, Andriy Mokhir: conceptualization, project administration, funding acquisition, resources, supervisions, writing – original draft, review and editing.

## Conflicts of interest

There are no conflicts to declare.

## Supplementary Material

MD-015-D3MD00609C-s001
