# Supplementary material for: A concept of dual-responsive prodrugs based on oligomerization-controlled reactivity of ester groups: an improvement of cancer cells versus neutrophils selectivity of camptothecin
Source: RSC Med Chem. 2024 Jan 31;15(4):1189–97. doi: 10.1039/d3md00609c (PMC11042170; doi:10.1039/d3md00609c)
Supplement: MD-015-D3MD00609C-s001 [file MD-015-D3MD00609C-s001.pdf]

Electronic Supplementary Material (ESI) for RSC Medicinal Chemistry.
This journal is © The Royal Society of Chemistry 2024

Supporting information

A concept of dual-responsive prodrugs based on oligomerization-controlled reactivity of ester groups: an improvement of cancer cells *versus* neutrophils selectivity of camptothecin

Insa Klemt, Viktor Reshetnikov, Subrata Dutta, Galyna Bila, Rostyslav Bilyy, Itziar Cossío Cuartero, Andrés Hidalgo, Adrian Wünsche, Maximilian Böhm, Marit Wondrak, Leoni A. Kunz-Schughart, Rainer Tietze, Frank Beierlein, Petra Imhof, Sabrina Gensberger-Reigl, Monika Pischetsrieder, Marlies Körber, Tina Jost, Andriy Mokhir*

Table of content

	Page
General	S4
Synthesis of the prodrug **2** and controls	S5-S22
Determination of stability of the prodrug **2** in solid state and in DMSO solution	S23-S25
n-Octanol/water partition coefficient (LogP) of the prodrug and controls	S26
Determination of solubility of the prodrug **2** and controls in aqueous solutions	S26-S27
UV-visible spectra of the prodrug **2** and intermediate **2c***H^+	S27
Dynamic light scattering (DLS) of solutions of the prodrug **2** and **2c***H^+	S27-S28

Stability of the prodrug **2** and control **3** at different pH's in the presence and absence of H_2O_2 monitored by fluorescence spectroscopy	S29-S32
Study of activation of the prodrug **2** and control **3** in the presence of different concentrations of H_2O_2 (0-10 mM)	S32
Monitoring release of iron ions from the prodrug **2**	S33
Monitoring the reaction of the prodrug **2** with H_2O_2 by using HPLC	S33-S34
Preparation of intermediate **2c***H^+	S35
Monitoring HO-cpt release from the prodrug **2** by using HPLC coupled to the mass detector	S35-S36
Monitoring the reaction of control **4** with H_2O_2 by using HPLC	S36-S38
Theoretical studies	S39-S44
Cell culture experiments	S45-S59
Cells and cell culture	S45
Determination of the viability of A2780, BL-2, DU-145 and NHDF cells	S45-S47
Determination of the viability of SAS, FaDu, ARPE-19 and HF cells	S47-S48
Clonogenic assay: testing effects of the prodrug and control on single cell colony formation capacity of cancer cells	S48-S49
Monitoring the uptake of the prodrug by BL-cells by quantification of intracellular fluorescence signal characteristic for camptothecin derivatives	S49-S50
Assessing the uptake of the prodrug by BL-2 cells via monitoring the intracellular concentration of boronic acid	S50-S51
Release of ROS in FaDu cells treated with IR	S52
Radiosensitizing effect of the prodrug and the control	S52-S53
Reaction of the prodrug with H_2O_2 in cells	S54-S56
Exploring the possibility that the prodrug induces the release of reactive oxygen species (ROS) in cells free settings and in cells	S56-S58
Effect of the prodrug on cell cycle of A2780 cells	S58-S59
Experiments with neutrophils	S59-S63
General	S59
Study of distribution of PDFPs	S59-S60
Study of cytotoxicity of the prodrug and drug HO-cpt in the presence of PMA	S60

Assessment of activation of prodrug **2** in blood and bone marrow in wild-type mice by monitoring PDFPs	S60-S62
Evaluation of effects of prodrug **2** and control HO-cpt on bone marrow and blood cells	S62-S63
In vivo effects of the prodrug on organs, tissues and cells in healthy animals	S64-S65
Mice	S64
Toxicity of the prodrug	S64-S65
Selection of the optimal dose of the prodrug	S65-S66
Accumulation of the prodrug in organs	S67-S68
Evaluation of the effect of the prodrug on the survival of mice after inoculation of Nemeth-Kellner (NK) lymphoma (Ly) NK/Ly	S68
References	S68-S71

General

Commercially available chemicals of the best quality from Sigma-Aldrich (Germany) were obtained and used without purification. Both MitoSOX™ Red (mitochondrial superoxide indicator, catalog: M36008) and CM-H2DCFDA (General Oxidative Stress Indicator, catalog: C6827) were obtained from Thermo Fischer Scientific (Germany). NUCLEAR-ID® Red DNA stain (catalog: ENZ-52406) was purchased from Enzo Life Science (Germany). NMR spectra were acquired on a Avance 300 or a Avance 400 spectrometer from Bruker (Germany). Electrospray ionization (ESI) or atmospheric pressure photoionization (APPI) mass spectra were recorded on a ESI MicroTOF II focus or a maXis 4G, both from Bruker (Germany). Elemental analysis (C, H, and N) was performed in the microanalytical laboratory of the chemical institutes of the Friedrich-Alexander-University of Erlangen-Nürnberg, Erlangen, Germany. UV-visible spectra were measured on either a Lambda Bio+ UV/Vis spectrophotometer (Perkin Elmer, Germany) or a Cary 100 UV-Vis Spectrophotometer (Agilent Technologies, Germany) by using either quartz glass cuvettes (Hellma GmbH, Germany) with a sample volume of 1 mL or micro-cuvettes with a sample volume of 100 µL (BRAND GmbH, Germany). Fluorescence spectra were acquired on a Cary Eclipse fluorescence spectrophotometer (Agilent Technologies, Germany) using fluorescence cuvettes (Hellma GmbH, Germany) with a sample volume of 1 mL. The fluorescence of live cells was quantified using either a Guava easyCyteTM 6-2L flow cytometer from Merck Millipore (Germany) or CytoFlex flow cytometer from Beckman Coulter, Inc (Germany). The data were processed using the inCyteTM software package from Merck Millipore (Germany) and ModFIT LTTM software from Verity Software House (USA). The microscopy images were taken with a Axio Vert.A1 microscope from Zeiss (Germany). The absorbance of solutions in microplates were measured in a Biotek Epoch Microplate Spectrophotometer from Agilent (USA) or a Varioskan LUX Multimode Microplate Reader from ThermoScientific (Germany). The latter was also used for fluorescence spectroscopy experiments in microplates. The purity of the prodrugs used in the biological assays was determined by elemental analysis (C, H, and N analysis). According to these data, the purity was greater than 95%. Statistical analysis of the data was conducted using paired Student's t-test if not stated otherwise. Two data sets were considered significantly different from each other when $p<0.05$.

Synthesis of the prodrug 2 and controls

Previously known compound **5**[S1] (main text, Figure 2) and intermediates **S1**,[S2] **S2**[S3] were synthesized as described elsewhere.

Synthesis of prodrug **2**

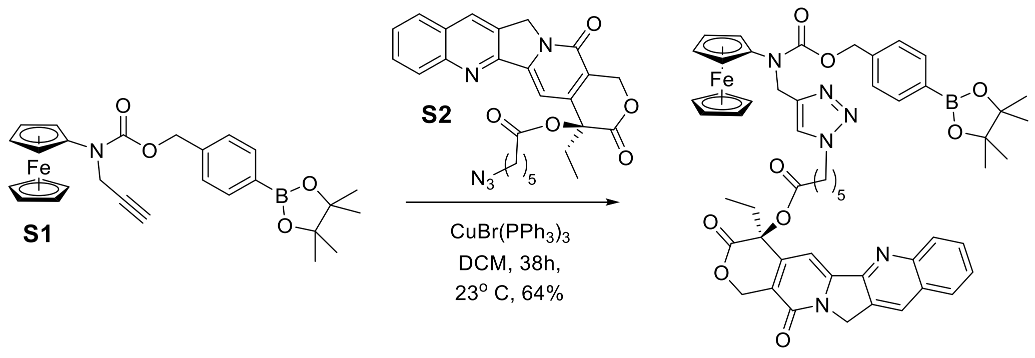

prodrug **2**

Bromotris(triphenylphosphine)copper(I) (111 mg, 120 µmol) was added to a solution of intermediates **S1** (300 mg, 600 µmol) and **S2**[S3] (293 mg, 600 µmol) in anhydrous dichloromethane (DCM, 15 mL). The resulting mixture was stirred at 23° C for 38 h under nitrogen atmosphere. The solvent was removed *in vacuo* (0.01 mbar) and the crude product was purified by column chromatography (silica gel, DCM/methanol 90/10, v/v, R_f = 0.57) and recrystallized at -20° C from acetonitrile. Product **2** was obtained as yellow solid (380 mg, 385 µmol, 64 %). ^{1}H NMR (300 MHz, chloroform-d1), δ = 8.39 (br. s., 1H), 8.21-8.18 (m., 1H), 7.96-7.92 (m., 1H), 7.84-7.78 (m., 2H), 7.67 (q, *J* = 7x(3) Hz, 2H), 7.58-7.47 (m., 1H), 7.34 (d, *J* = 7.5 Hz, 2H), 7.21-7.19 (m., 1H), 5.69 (d, *J* = 17.1 Hz, 1H), 5.41 (d, *J* = 17.3 Hz, 1H), 5.29 (s., 2H), 5.17 (s., 2H), 4.64 (br. s., 2H), 4.14 (br. s., 7H), 2.25-2.45 (m., 2H), 2.32-2.21 (m., 2H) 1.85 (br. s., 2H), 1.72-1.58 (m., 2H), 1.46-1.21 (m., 14H), 1.01-0.95 (m., 3H). ^{1}H NMR spectrum as well as assignments of all proton signals are provided in Figure S1. ^{13}C NMR (75 MHz, chloroform-d1) δ = 172.23, 167.47, 157.31, 152.33, 148.81, 146.23, 145.81, 139.69, 129.51, 128.52, 128.45, 128.40, 128.19, 128.15, 128.02, 120.26, 95.82, 83.88, 77.20, 75.80, 75.75, 69.09, 67.09, 51.11, 49.93, 49.90, 33.41, 31.82, 29.84, 29.65, 25.74, 24.84, 23.95, 7.54. ^{13}C NMR spectrum as well as assignments of all carbon signals are provided in Figure S2. HR-ESI-MS (positive mode),

m/z: calcd. 986.3477 for $C_{53}H_{55}BFeN_6O_9$ $[M-e^-]^+$, found 986.3486. Elemental analysis: calcd (%) for $C_{53}H_{55}BFeN_6O_9·0.33CH_3CN$: C 64.43, H 5.64, N 8.86; found: C 64.46, H 5.51, N 8.98.

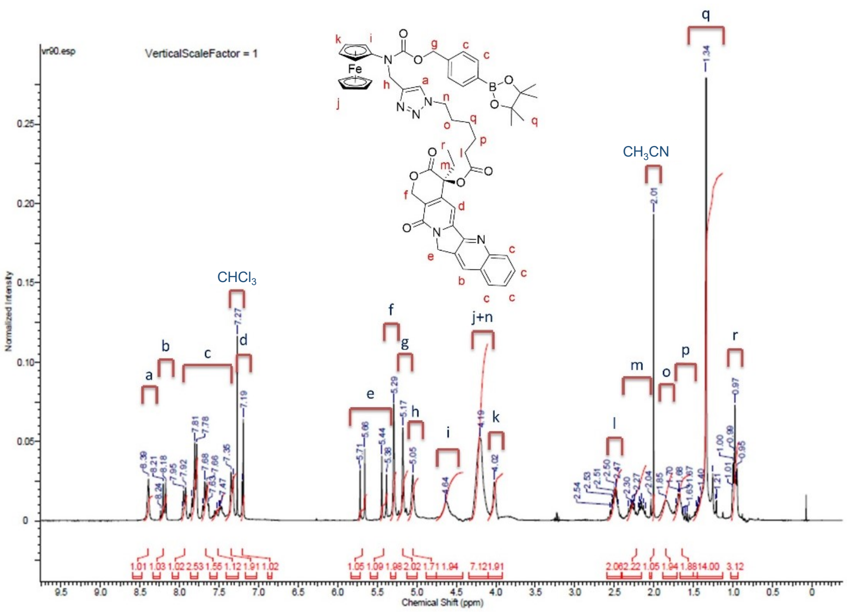

Figure **S1**. ^{1}H NMR spectrum of prodrug **2**. Solvent: $CDCl_3$.

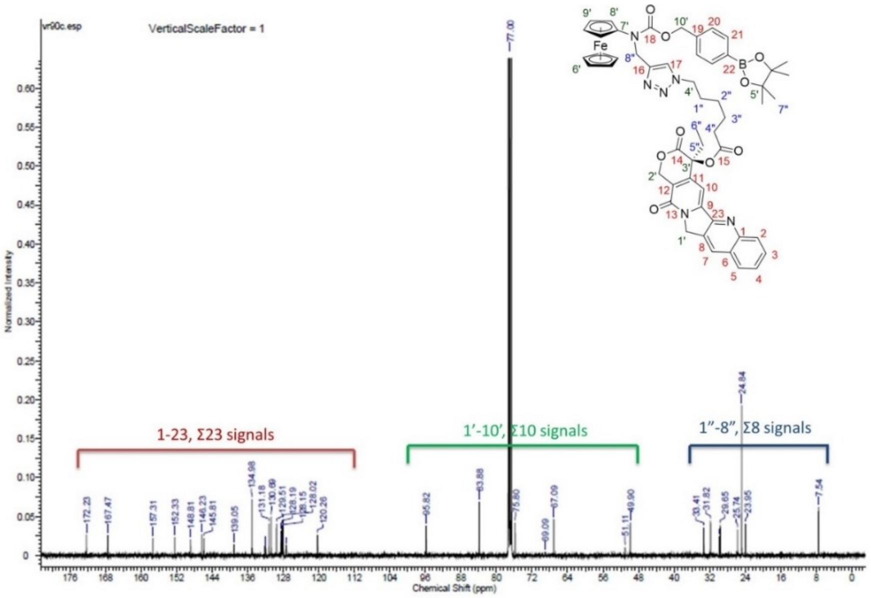

Figure **S2**. ^{13}H NMR spectrum of prodrug **2**. Solvent: $CDCl_3$.

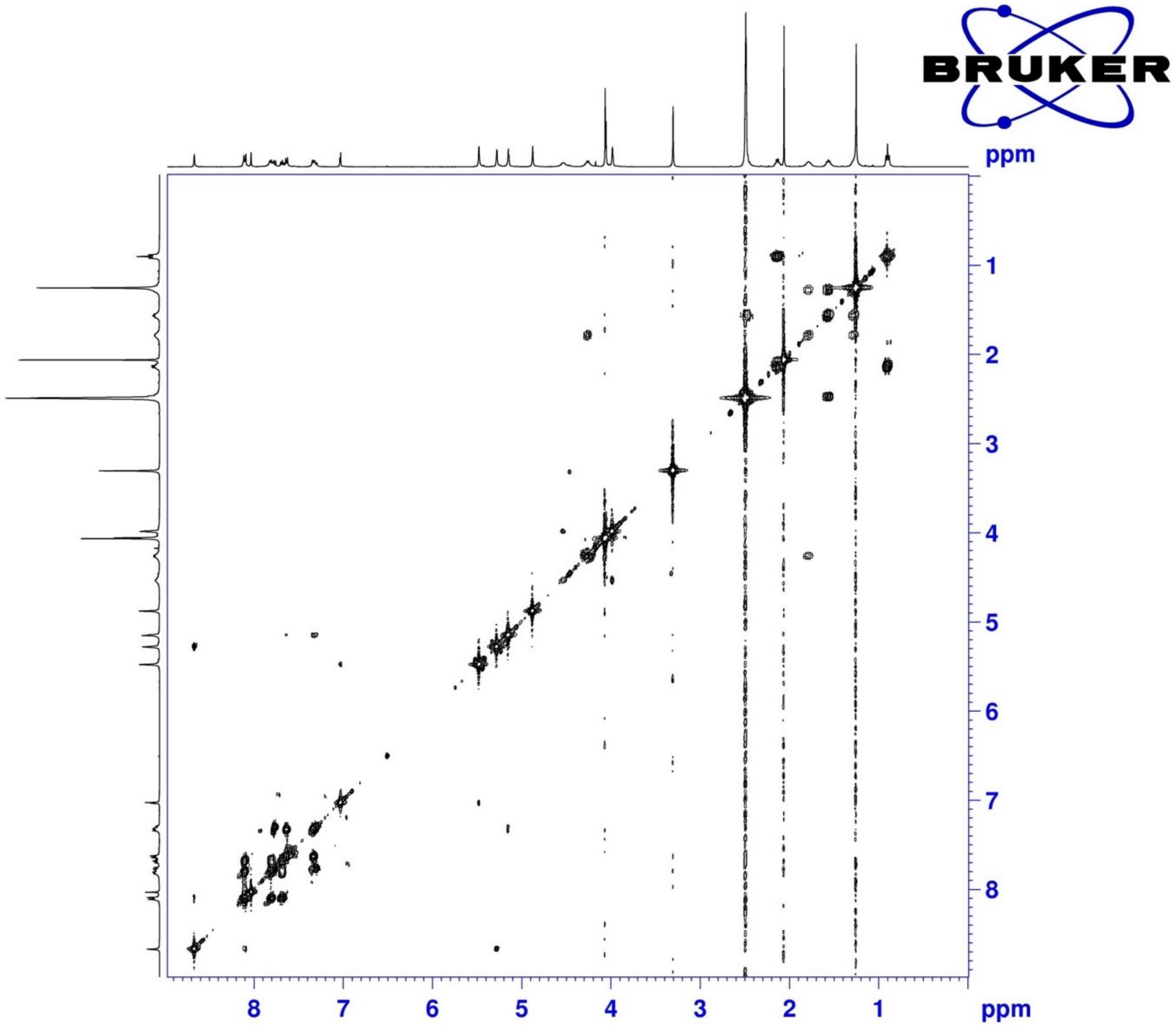

Figure **S3**. $^{1}H,^{1}H$-COSY NMR spectrum of prodrug **2**. Solvent: $CDCl_3$.

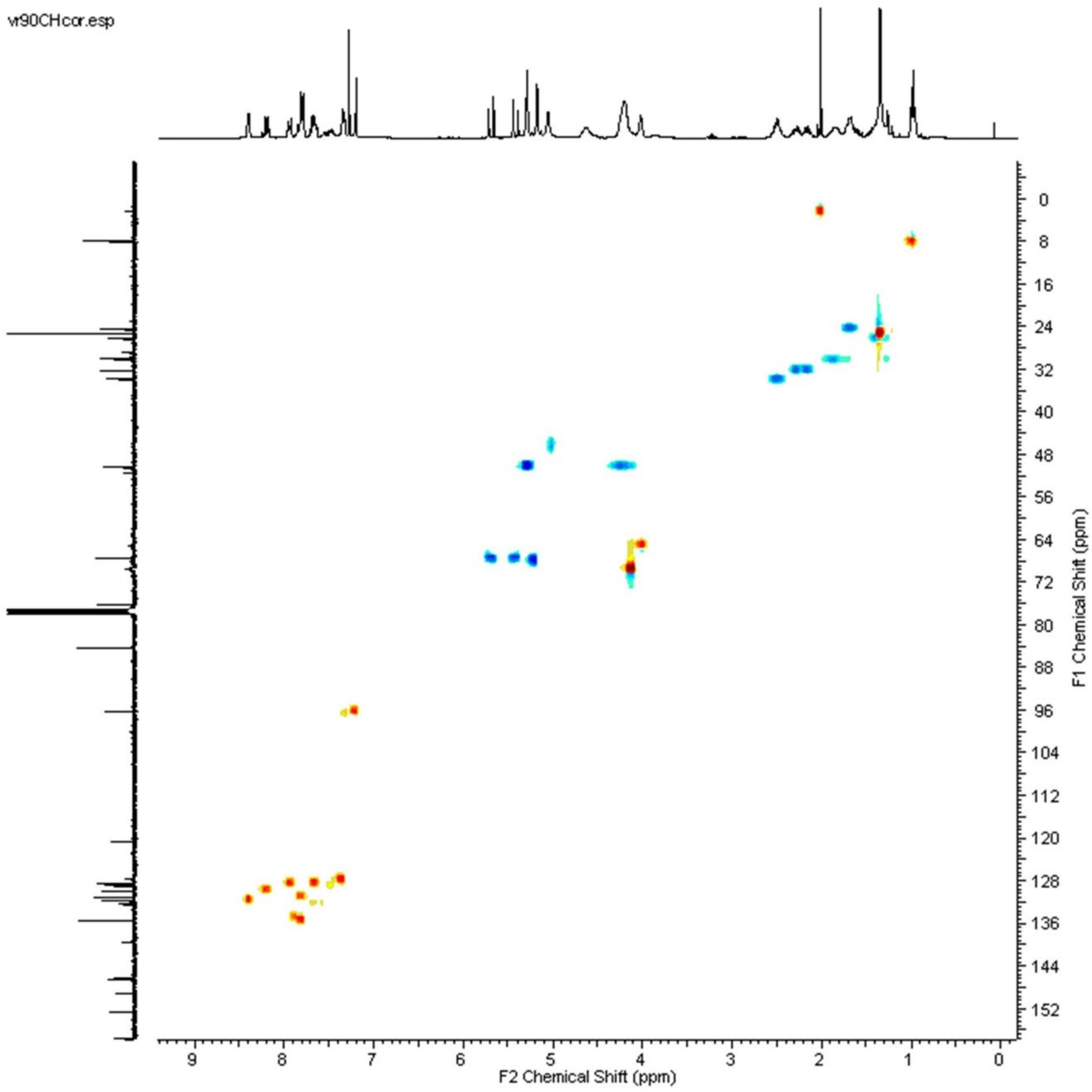

Figure **S4**. $^{1}H,^{13}C$-HSQC NMR spectrum of prodrug **2**. Solvent: $CDCl_3$.

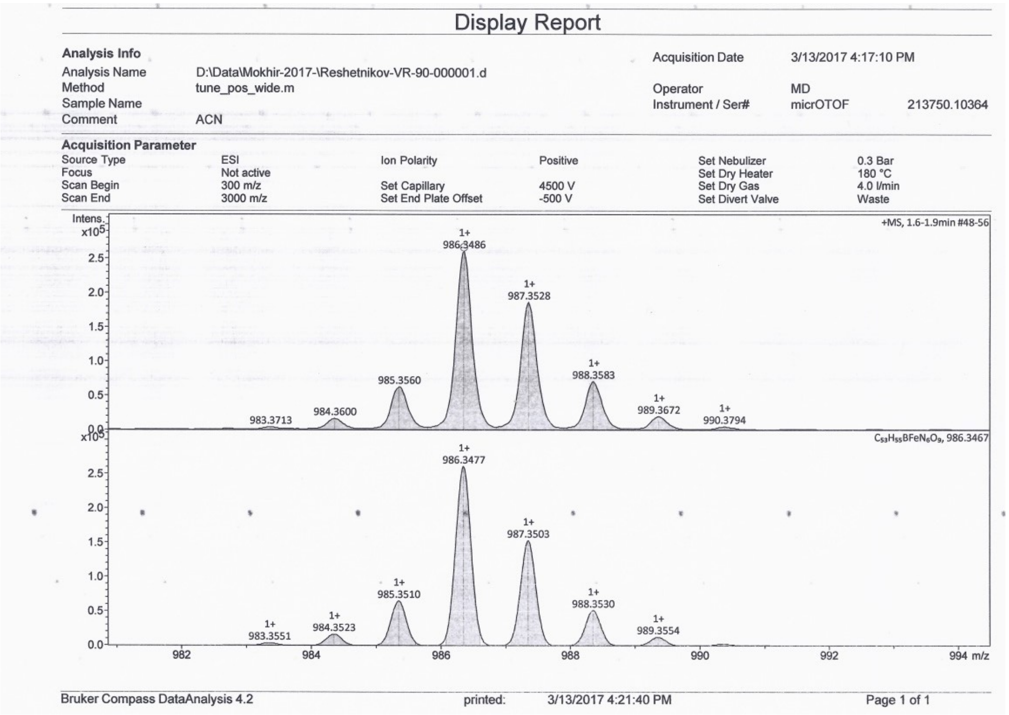

Figure **S5**. Experimental high resolution mass spectrum of prodrug **2** (upper plot) as well as its calculated mass spectrum (lower plot).

Synthesis of **S4**

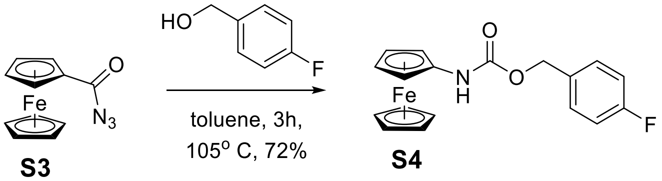

S3 **S4**

Acylazidoferrocene **S3** (7.00 g, 27,45 mmol) was dissolved in toluene (100 mL), the solution was heated to 105 °C, and (4-flourophenyl)methanol (4.50 g, 35.68 mmol) was added in a single portion. The red solution obtained was stirred at 105 °C for 3 h, by which time the color was changed to dark orange. The mixture was cooled to 23 °C and the solvent was removed *in vacuo* (0.01 mbar) to give orange solid. The product was purified by column chromatography (silica gel, DCM / acetone 99/1, v/v, R_f =0.33). **S4** was obtained as yellow solid (7.01 g, 72%). ^{1}H NMR (400 MHz, chloroform-d1), δ = 7.37 (br. s., 2H), 7.07 (t., J= 7.6x(2) Hz, 2H), 5.94 (br. s., 1H), 5.12 (br. s., 2H), 4.65 (br. s., 2H),

4.25-4.14 (m., 7H). ^{1}H NMR spectrum as well as assignments of all proton signals are provided in Figure S6. ^{13}C NMR (101 MHz, chloroform-d1) δ = 163.72, 161.27, 153.33, 131.95, 130.15, 130.06, 115.48, 115.27, 97.39, 69.83, 66.09, 64.85, 60.77. ^{13}C NMR spectrum as well as assignments of all carbon signals are provided in Figure S7. ^{19}F-NMR (377 MHz, chloroform-d1): δ = -113.55. HR-ESI-MS (positive mode), *m/z*: calcd. 353.0509 for $C_{18}H_{16}FeFNO_2$ $[M-e^-]^+$, found 353.0510. Elemental analysis: calcd (%) for $C_{18}H_{16}FeFNO_2$: C 61.22, H 4.57, N 3.97; found: C 61.09, H 4.55, N 3.85.

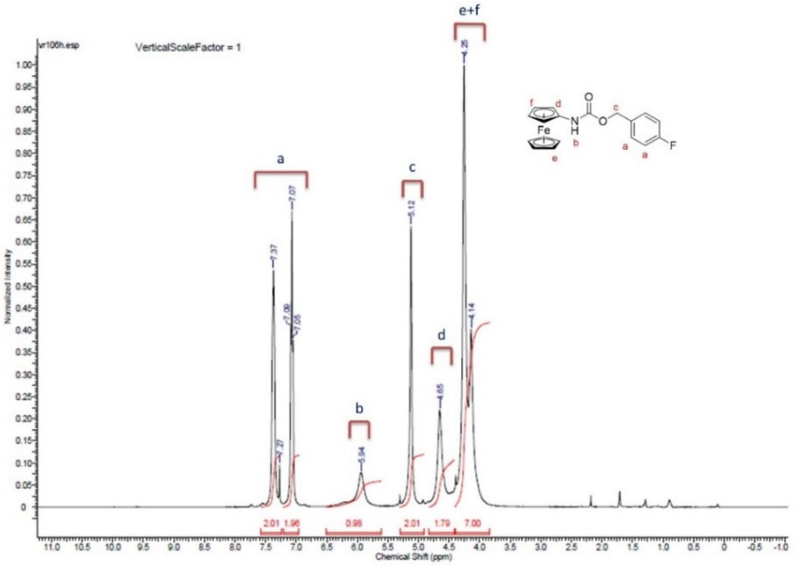

Figure **S6**. ^{1}H NMR spectrum of intermediate **S4**. Solvent: $CDCl_3$.

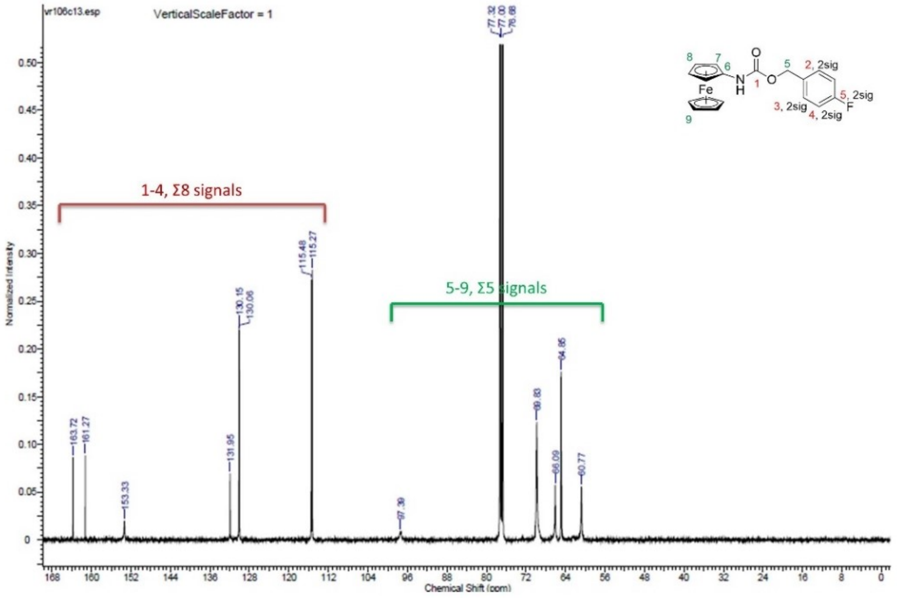

Figure **S7**. ^{13}C NMR spectrum of intermediate **S4**. Solvent: $CDCl_3$.

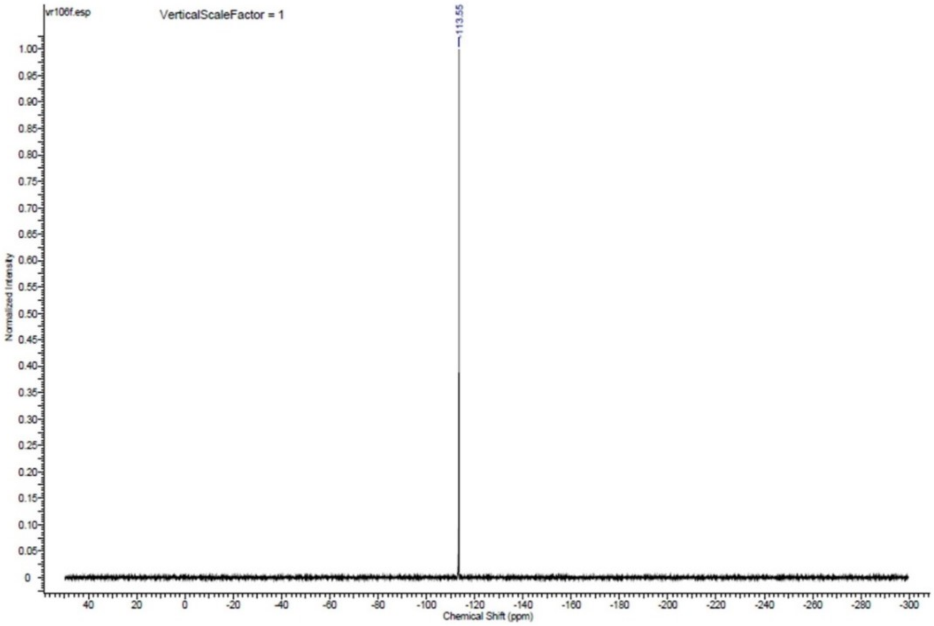

Figure **S8**. ^{19}F NMR spectrum of intermediate **S4**. Solvent: $CDCl_3$.

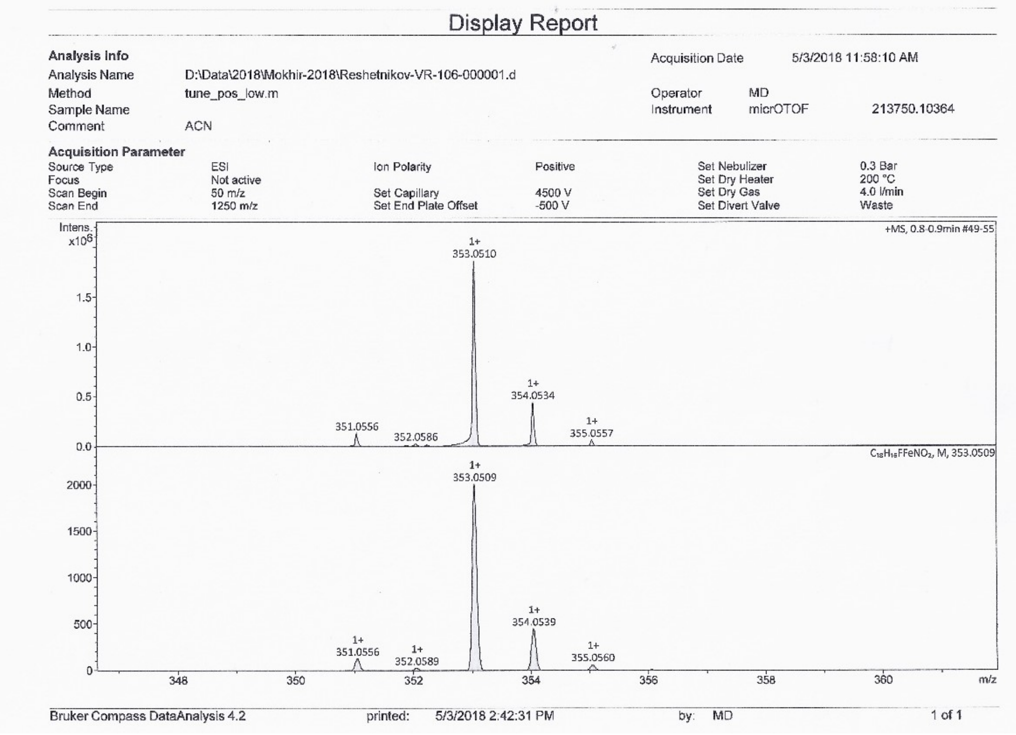

Figure **S9**. Experimental high resolution mass spectrum of intermediate **S4** (upper plot) as well as its calculated mass spectrum (lower plot).

Synthesis of **S5**

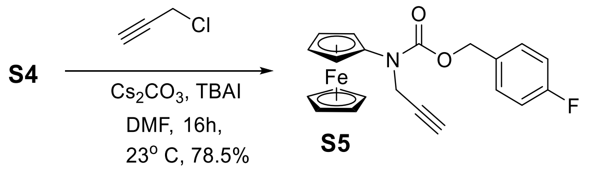

Intermediate **S4** (3.00 g, 8.49 mmol) Cs_2CO_3 (11.07 g, 34.00 mmol) and TBAI (12.52 g, 34.00 mmol) were dissolved in anhydrous DMF (20 mL) under nitrogen atmosphere. Afterwards propargyl chloride (2.53 g, 34.00 mmol) was added and the mixture was stirred for 18 h at 23 °C. Then, the solvent was removed *in vacuo* (0.01 mbar) and the product was purified by column chromatography (silica gel, DCM / acetone 99/1, v/v, R_f =0.56). Product **S5** was obtained as yellow solid (2.61 g, 78.5 %). ^{1}H NMR (400 MHz, chloroform-d1), δ = 7.48 (dd, *J* = 8.6, 5.6 Hz, 2H), 7.24-7.20 (m., 2H), 5.15 (s, 2H), 4.50 (br. s., 2H), 4.45 (d, *J* = 2.3 Hz, 2H), 4.18 (s., 5H), 4.05 (t., J= 2x(2) Hz, 2H), 3.33 (s., 2H). ^{1}H NMR spectrum as well as assignments of all proton signals are provided in Figure S10. ^{13}C NMR (101MHz, chloroform-d1) δ = 163.54, 161.12, 153.85, 133.08, 130.67, 115.84, 115.63, 100.42, 81.10, 75.15, 69.39, 66.88, 64.79, 62.70. ^{13}C NMR spectrum as well as assignments of all carbon signals are provided in Figure S11. ^{19}F-NMR (377 MHz, chloroform-d1): δ = -114.05. HR-ESI-MS (positive mode), *m/z*: calcd. 391.0666 for $C_{21}H_{18}FFeNO_2$ $[M-e^-]^+$, found 391.0653. Elemental analysis: calcd (%) for $C_{21}H_{18}FFeNO_2$: C 64.47, H 4.64, N 3.58; found: C 64.43, H 4.72, N 3.71.

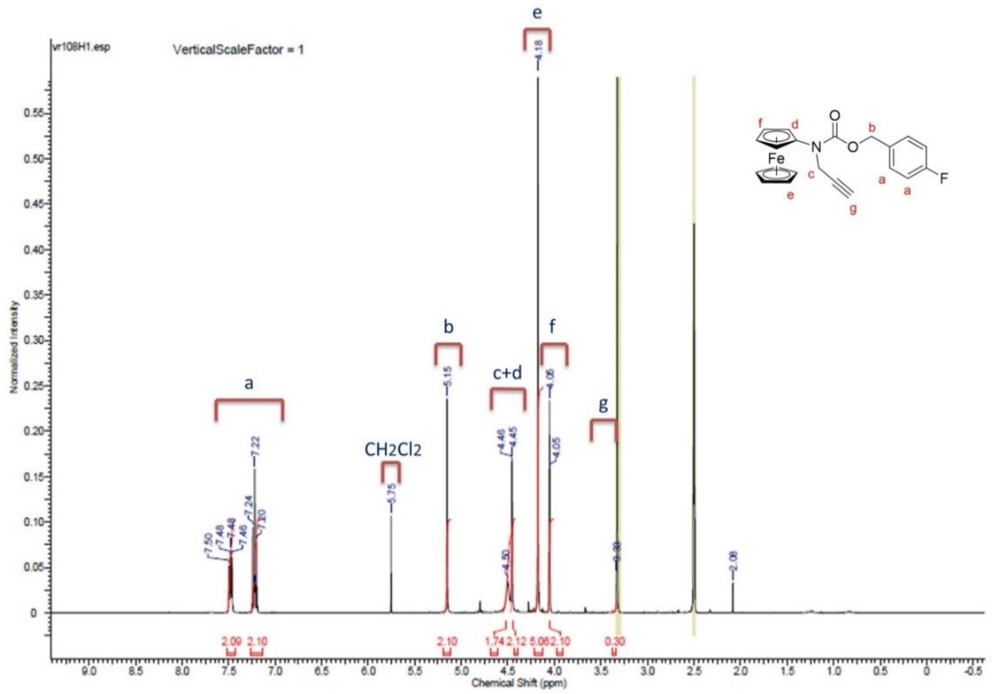

Figure **S10**. ^{1}H NMR spectrum of intermediate **S5**. Solvent: $CDCl_3$.

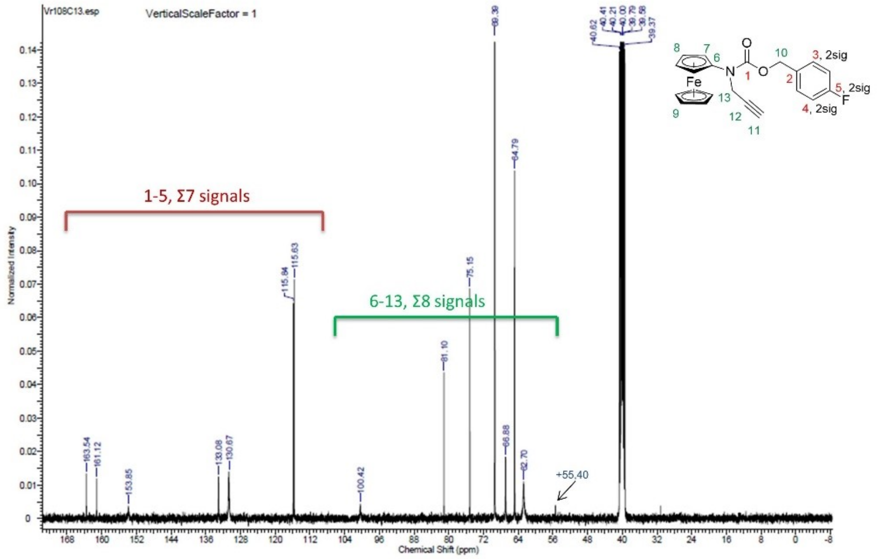

Figure **S11**. ^{13}C NMR spectrum of intermediate **S5**. Solvent: $CDCl_3$.

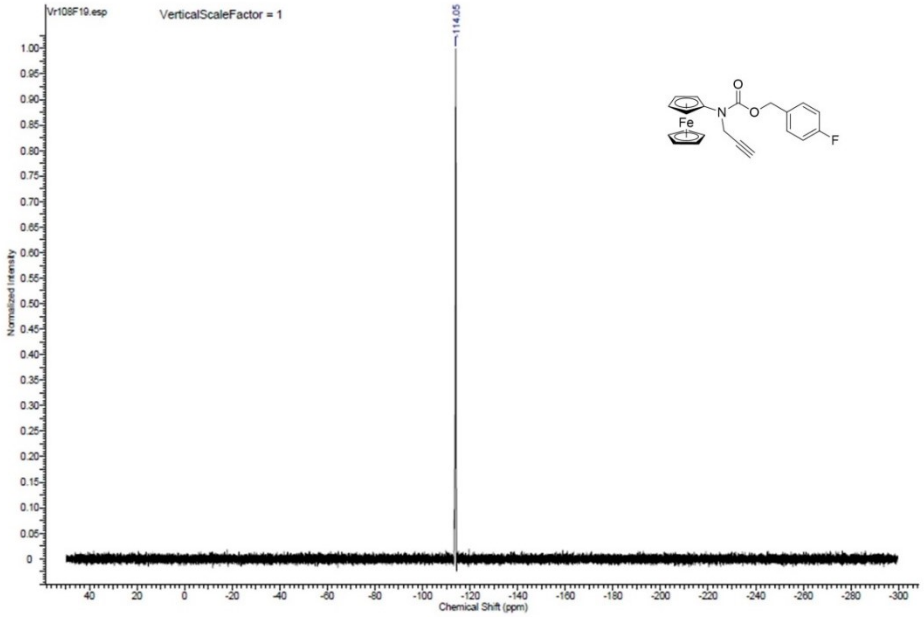

Figure **S12**. ^{19}F NMR spectrum of intermediate **S5**. Solvent: $CDCl_3$.

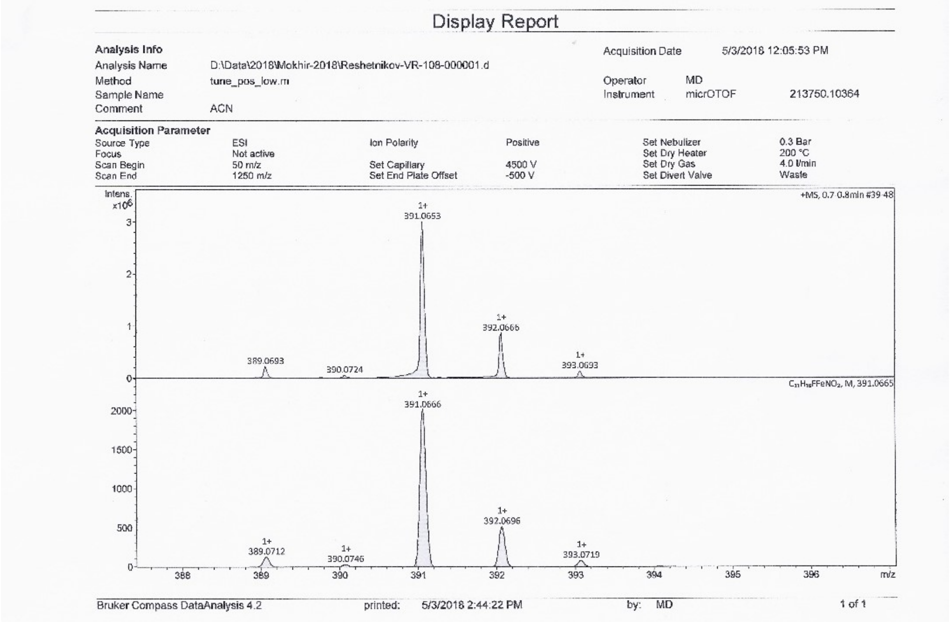

Figure **S13**. Experimental high resolution mass spectrum of intermediate **S5** (upper plot) as well as its calculated mass spectrum (lower plot).

Synthesis of control **3**

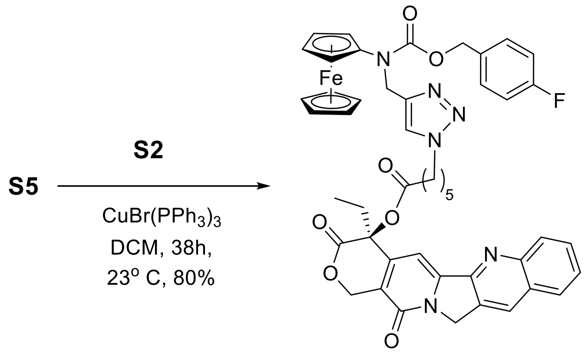

control **3**

To a solution of intermediates **S2** (200 mg, 410 µmol) and **S5** (200 mg, 512 µmol) in anhydrous DCM (12 mL) was added bromotris(triphenylphosphine)copper(I) (76 mg, 82 µmol). The resulting solution was stirred at 23° C for 38 h under nitrogen atmosphere. The solvent was removed *in vacuo* (0.01 mbar) and the crude product was purified by column chromatography (silica gel, DCM / methanol 95/5, v/v, R_f = 0.39) and recrystallized at -20° C from acetonitrile. Product **3** was obtained as yellow solid (290 mg, 330 µmol, 80 %). ^{1}H NMR (400 MHz, chloroform-d1), δ = 8.4 (s., 1H), 8.19 (d, *J* = 8.5 Hz, 1H), 7.95-7.93 (m., 1H), 7.82 (ddd, *J* = 8.5, 7.0, 1.3 Hz, 1H),), 7.67 (ddd, *J* = 8.1, 6.9, 1.0 Hz, 1H), 7.34 (dd, *J* = 8.5, 5.5 Hz, 2H), 7.20 (s., 1H), 7.06-7.02 (m., 2H), 5.69 (d, *J* = 17.3 Hz, 1H), 5.42 (d, *J* = 17.3 Hz, 1H), 5.30 (s, 2H), 5.15 (s., 2H), 5.01 (s., 2H), 4.59 (br. s., 2H), 4.23 (br. s., 2H), 4.13 (br. s., 5H), 4.01 (br. s., 2H), 2.57-2.42 (m., 2H), 2.33-2.11 (m., 2H), 1.85 (br. s., 2H) 1.73-1.64 (m., 2H), 1.39 (br.s., 2H), 0.98 (t., J= 7.5x(2) Hz, 3H). ^{1}H NMR spectrum as well as assignments of all hydrogen signals are provided in Figure S14. ^{13}C NMR (101MHz, chloroform-d1) δ = 172.26, 167.52, 163.83, 161.38, 157.33, 52.37, 148.84, 146.25, 145.82, 145.06, 132.05, 131.94, 131.20, 130.71, 130.31, 130.25, 129.52, 128.49, 128.22, 128.17, 128.04, 120.27, 119.82, 115.59, 115.38, 95.84, 77.20, 75.85, 68.97, 67.11, 64.61, 63.03, 49.98, 49.91, 33.41, 31.84, 29.86, 25.73, 23.95, 7.56, 1.88. ^{13}C NMR spectrum as well as assignments of all carbon signals are provided in Figure S15. ^{19}F-NMR (377 MHz, chloroform-d1): δ = -113.47. HR-ESI-MS (positive mode), *m/z*: calcd. 878.2522 for $C_{47}H_{43}BFFeN_6O_7$ $[M-e^-]^+$, found 878.2516. Elemental analysis: calcd (%) for $C_{47}H_{43}BFFeN_6O_7{\cdot}0.33CH_3CN$: C 64.15, H 4.97, N 9.94; found: C 64.05, H 5.05, N 10.03.

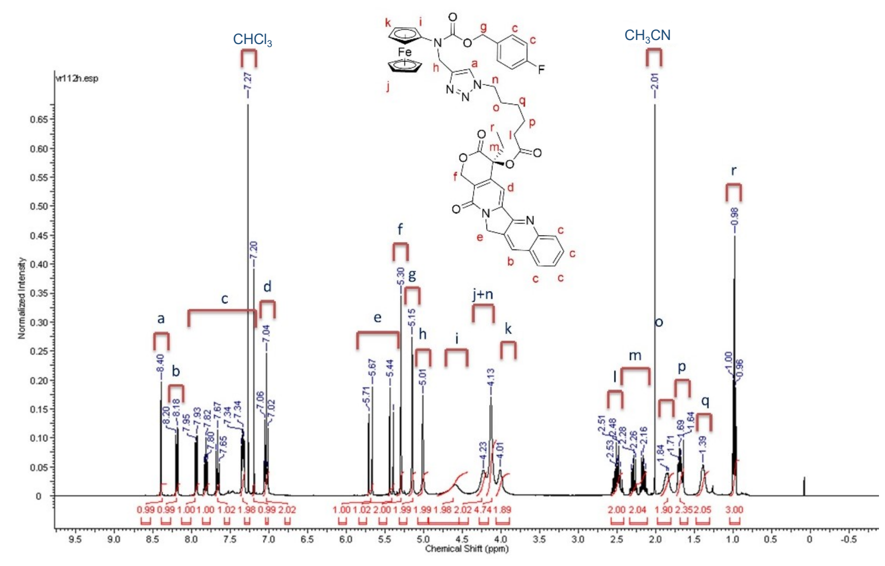

Figure **S14**. ^{1}H NMR spectrum of intermediate **3**. Solvent: $CDCl_3$.

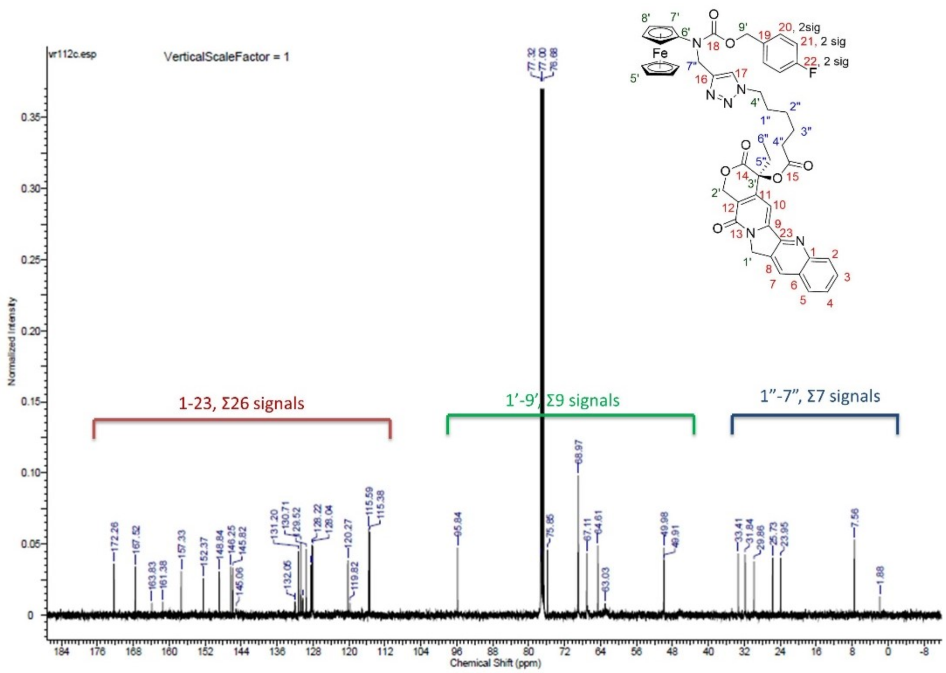

Figure **S15**. ^{13}C NMR spectrum of intermediate **3**. Solvent: $CDCl_3$.

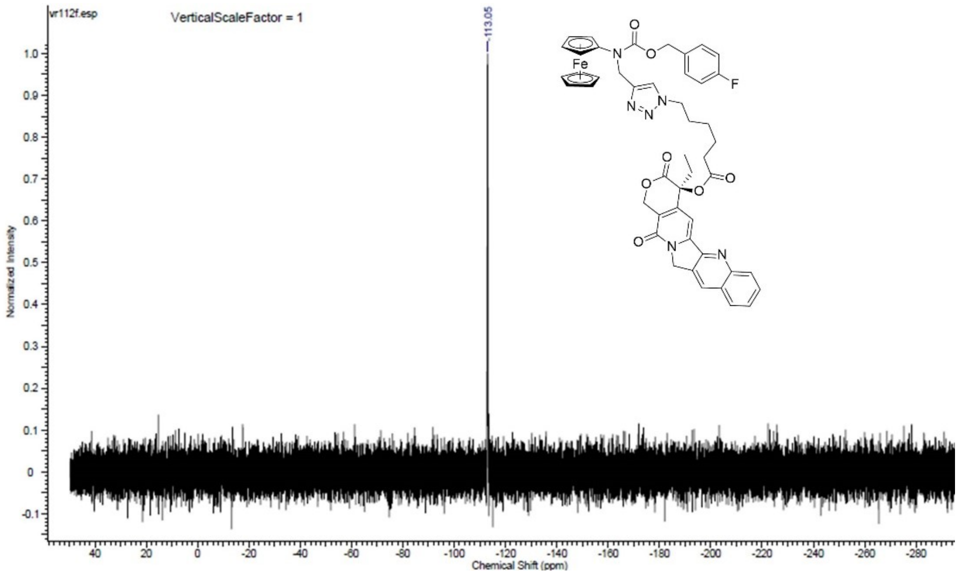

Figure **S16**. ^{19}F NMR spectrum of intermediate **3**. Solvent: $CDCl_3$.

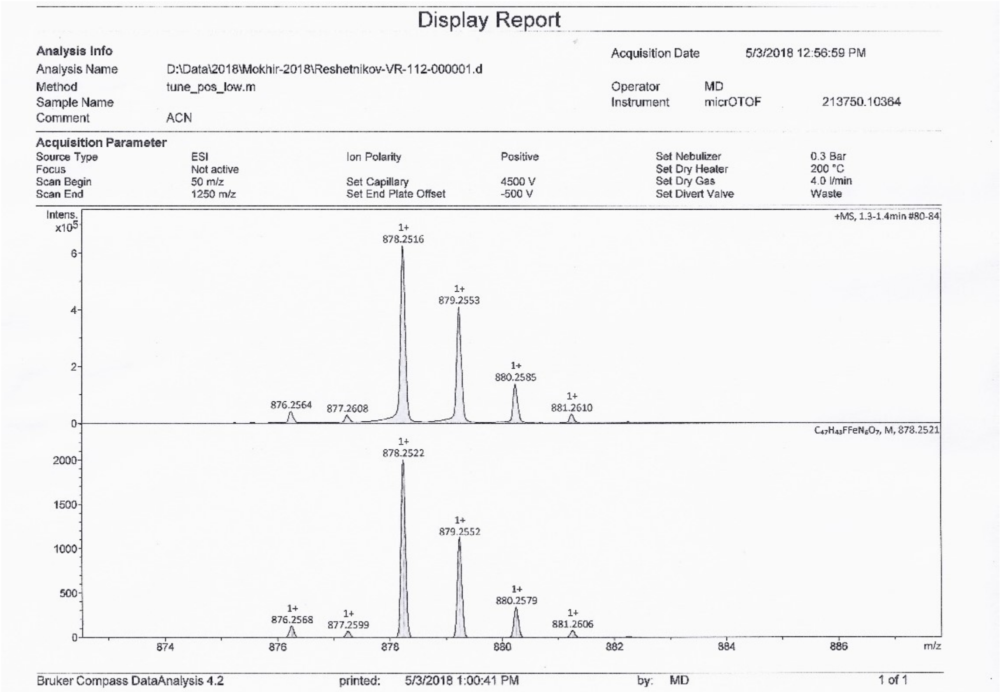

Figure **S17**. Experimental high resolution mass spectrum of intermediate **3** (upper plot) as well as its calculated mass spectrum (lower plot).

Synthesis of intermediate **S7**

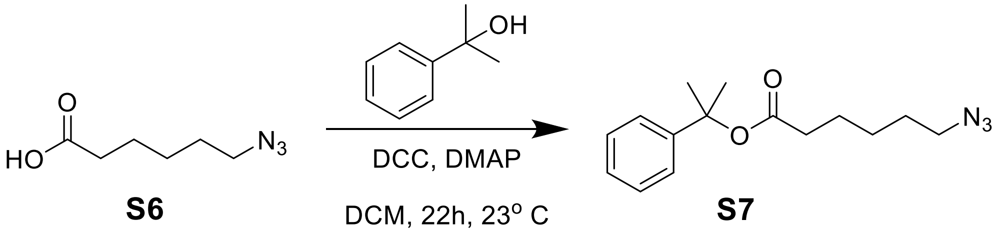

Intermediate **S6**[S4] (1.37 g, 8.81 mmol) and 1-methyl-1-phenylethyl alcohol (800 mg, 5.87 mmol) were dissolved in DCM (10 mL) and 4-(dimethylamino)pyridine (DMAP, 72 mg, 590 µmol) was added. The reaction solution was cooled to 0°C and N,N′-dicyclohexylcarbodiimide (DCC, 1.33 g, 6.46 mmol) was then added while stirring. The reaction mixture was stirred for 22h at 23°C. After completion of the reaction, 1,3-dicyclohexylurea (DCU, white precipitate) was filtered off and washed with DCM (30 mL). The filtrate was then washed with aqueous HCl (15 ml, 0.5 M) and with saturated sodium hydrogen sulfate solution (15 ml). The organic phase was finally dried over $MgSO_4$ and the solvent was removed *in vacuo* (0.01 mbar). The crude product was then purified by column chromatography (silica gel, cyclohexane / ethyl acetate 4/1, v/v, R_f = 0.42). **S7** was obtained as colorless oil (660 mg, 2.40 mmol, 41 %). ^{1}H NMR (400 MHz, $CDCl_3$) δ 7.37 – 7.30 (m, 4H), 7.27 – 7.22 (m, 1H), 3.26 (t, *J* = 6.9 Hz, 2H), 2.31 (t, *J* = 7.4 Hz, 2H), 1.77 (s, 6H), 1.67 – 1.56 (m, 4H), 1.45 – 1.33 (m, 2H). ^{1}H NMR spectrum as well as assignments of all hydrogen signals are provided in Figure S18. ^{13}C NMR (101 MHz, $CDCl_3$) δ 172.21, 146.02, 128.40, 127.11, 124.37, 81.57, 51.39, 35.25, 28.77, 26.35, 24.58. ^{13}C NMR spectrum as well as assignments of all carbon signals are provided in Figure S19. HR-ESI-MS (positive mode), *m/z*: calcd. 298.1526 for $C_{15}H_{21}N_3NaO_2$ [M+Na]+, found 298.1532.

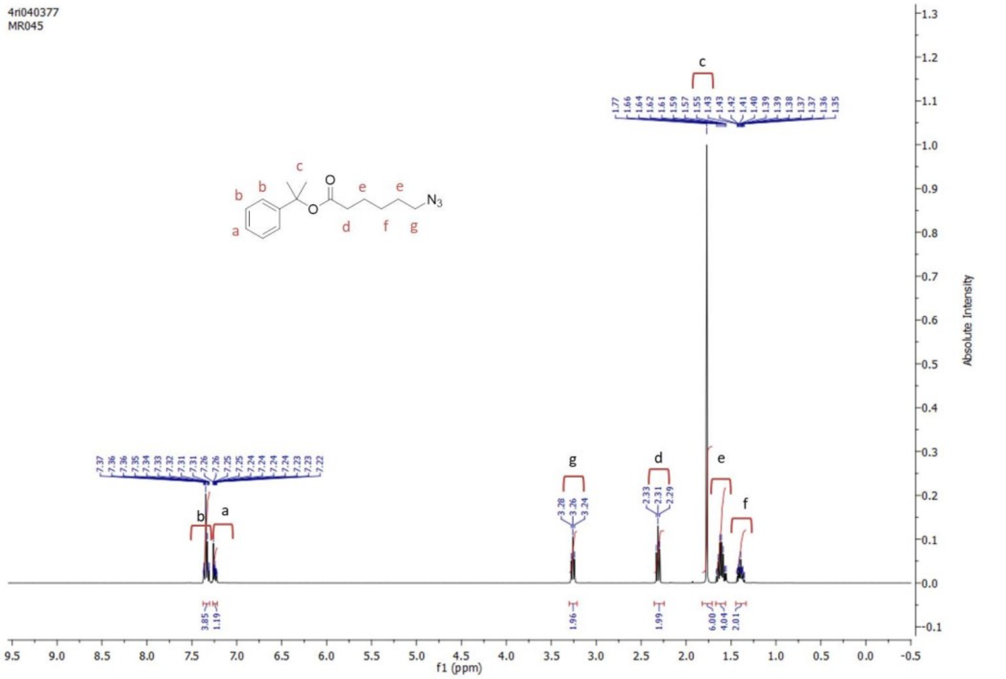

Figure **S18**. ^{1}H NMR spectrum of intermediate **S7**. Solvent: $CDCl_3$.

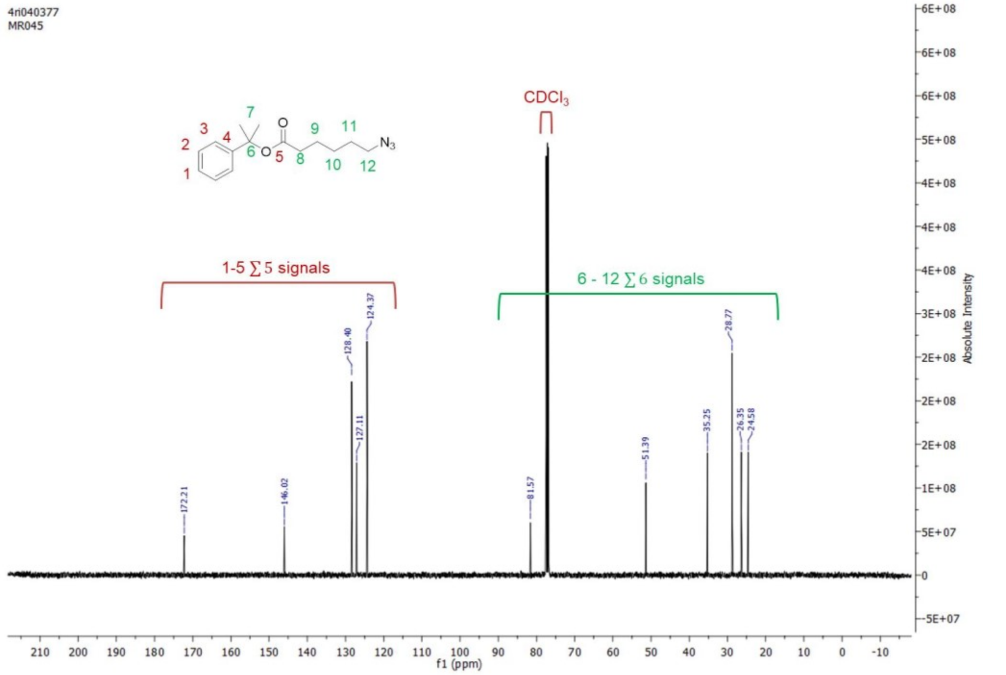

Figure **S19**. ^{13}C NMR spectrum of intermediate **S7**. Solvent: $CDCl_3$.

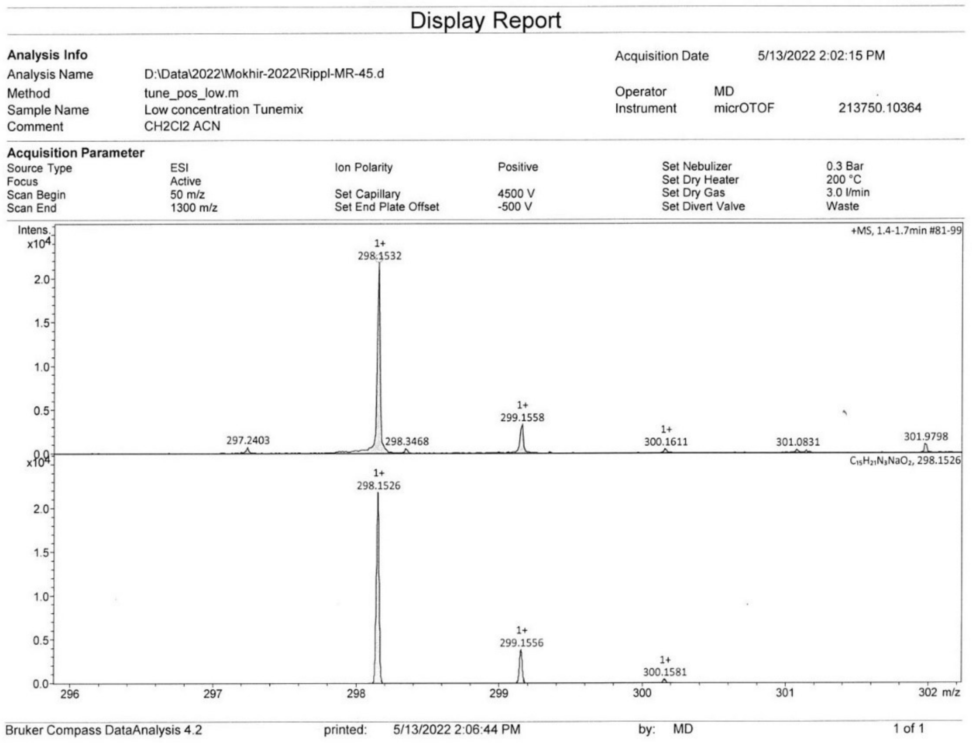

Figure **S20**. Experimental high resolution mass spectrum of intermediate **S7** (upper plot) as well as its calculated mass spectrum (lower plot).

<u>Synthesis of control **4**</u>

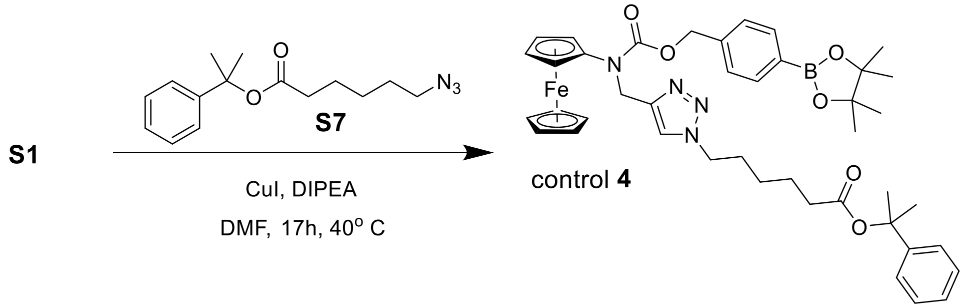

Copper(I) iodide (19 mg, 100 µmol) was added to a solution of intermediates **S1** (100 mg, 200 µmol) and **S7** (61 mg, 220 µmol) in anhydrous DMF (400 µL). Subsequently, N,N-diisopropylethylamine (DIPEA, 105 µL, 600 µmol) was added and the resulting solution was stirred at 40° C for 17 h under nitrogen atmosphere. The solvent was removed *in vacuo* (0.01 mbar) and the crude product was purified by column chromatography (silica gel, cyclohexane / acetone 80/20, v/v, R_f = 3.23). Product **4** was obtained as orange solid (120 mg, 155 µmol, 78 %). ^{1}H NMR (400 MHz, Acetone-d6) δ 7.76 (d, *J* = 8.1 Hz, 2H),

7.74 – 7.65 (m, 1H), 7.42 (dd, *J* = 16.0, 4.7 Hz, 2H), 7.39 – 7.33 (m, 2H), 7.34 – 7.26 (m, 2H), 7.25 – 7.18 (m, 1H), 5.24 (s, 2H), 4.99 (s, 2H), 4.68 (s, 2H), 4.36 (s, 2H), 4.13 – 4.06 (m, 5H), 4.01 – 3.95 (m, 2H), 2.29 (t, *J* = 7.4 Hz, 2H), 1.92 – 1.80 (m, 2H), 1.71 (s, 6H), 1.60 (dt, *J* = 15.1, 7.4 Hz, 2H), 1.33 (s, 12H), 1.28 (s, 2H). ^{1}H NMR spectrum as well as assignments of all hydrogen signals are provided in Figure S21. ^{13}C NMR (101 MHz, Acetone-d6) δ 172.15, 147.22, 145.59, 140.82, 135.65, 135.60, 128.94, 128.15, 127.47, 125.12, 123.73, 84.59, 81.71, 69.65, 67.82, 65.07, 50.39, 35.45, 30.72, 29.05, 26.59, 25.20, 25.02. ^{13}C NMR spectrum as well as assignments of all carbon signals are provided in Figure S22. HR-ESI-MS (positive mode), *m/z*: calcd. 774.3254 for $C_{42}H_{51}BFeN_4O_2$ $[M-e^-]^+$, found 774.3240. Elemental analysis: calcd (%) for $C_{42}H_{51}BFeN_4O_2$: C 65.13, H 6.64, N 7.23; found: C 64.98, H 6.76, N 7.18.

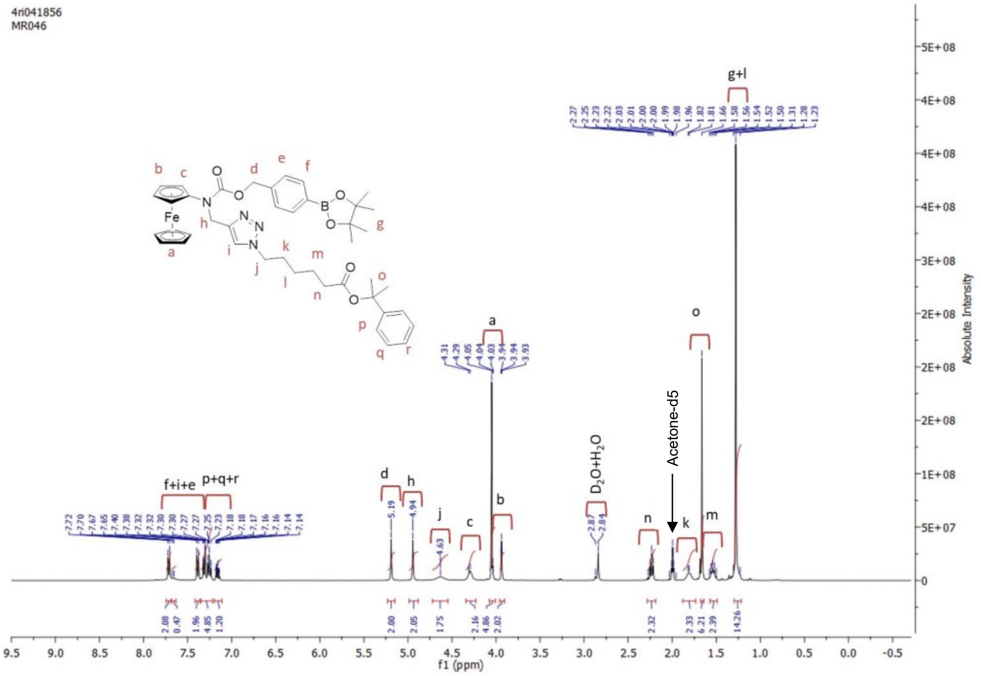

Figure **S21**. ^{1}H NMR spectrum of prodrug **4**. Solvent: acetone-d6.

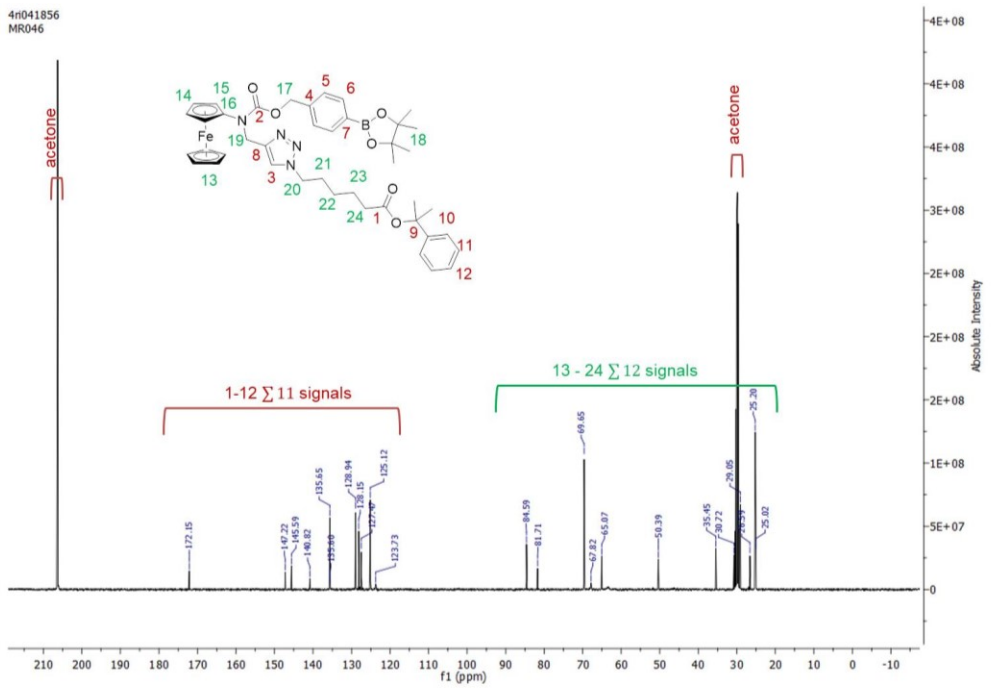

Figure **S22**. ^{13}C NMR spectrum of prodrug **4**. Solvent: acetone-d6.

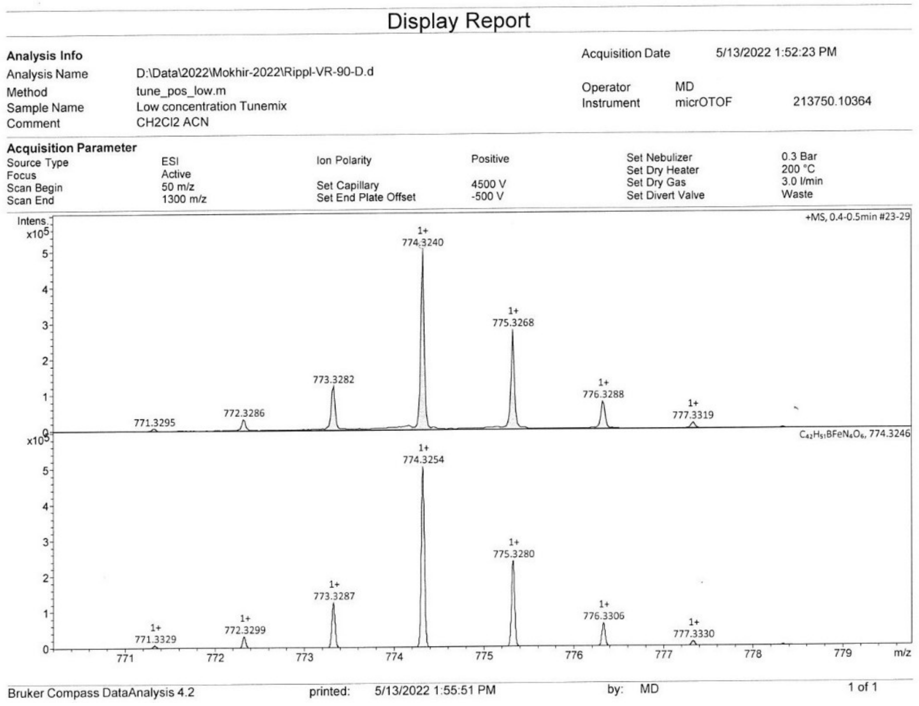

Figure **S23**. Experimental high resolution mass spectrum of prodrug **4** (upper plot) as well as its calculated mass spectrum (lower plot).

Determination of stability of the prodrug 2 in solid state and in DMSO solution

For cellular as well as in vivo experiments running over several days, it is common to first prepare stock solutions of (pro)drugs, keep them at low temperature for the duration of the experiment and use when needed. For compounds, which are unstable in DMSO, the alternative way would be to prepare fresh stock solutions and use them up immediately. To find out which of these possibilities is better suitable for studies of prodrug **2**, we evaluated the stability of its concentrated DMSO solution (10 mM) as well as the corresponding solid samples stored at different conditions including (a) at -20 °C in dark, (b) at 6 °C in dark, (c) at 22 °C in dark, (d) at 22 °C and under daylight. We used thin layer chromatography (TLC) and for fluorescence spectroscopy in these studies.

TLC

A small portions of solid **2** stored under conditions (a)-(d) for definite time periods were dissolved in CH_2Cl_2 (DCM) and spotted on the TLC plate with silica containing a fluorescent indicator as a stationary phase. The TLC was performed using the solvent mixture Et_2O/DCM/EtOAc (2/2/6; v/v/v) as an eluent. The DMSO solutions were spotted directly on the TLC plate and ran first with pure Et_2O as an eluent to eliminate DMSO. After drying, the TLC was run for the second time using the solvent mixture Et_2O/DCM/EtOAc (2/2/6; v/v/v). The resolved in this way mixtures were visualized by the observation of fluorescence quenching by mixture components (dark spots) under UV light (254 nm) (Figure S24A, B).

Fluorescence spectroscopy

DMSO solutions of **2** (10 mM) stored under conditions (a)-(d) for definite time periods were diluted with phosphate-saline buffer (10 mM phosphate, pH 7, 150 mM NaCl) to the concentration of 10 μM and mixed thoroughly. Emission of the resulting solutions at 445 nm ($\lambda_{excitation}$= 300 nm) was measured by using Cary Eclipse fluorescence spectrophotometer (Figure S24C).

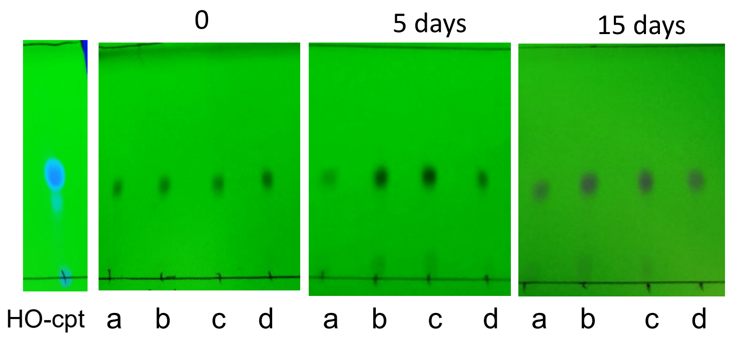

Figure **S24A**: TLC analysis of samples of solid prodrug **2** stored at different conditions (a-d) for 0-15 days. Conditions: (a) -20 °C, in dark; (b) 6 °C, in dark; (c) 22 °C, in dark; (d) 22 °C, under daylight.

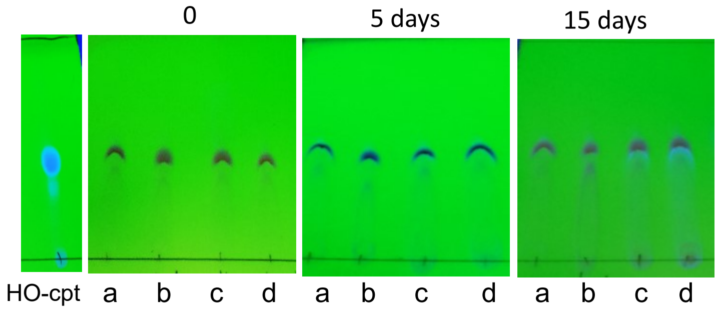

Figure **S24B**: TLC analysis of samples of solution of **2** in DMSO (10 mM) stored at different conditions (a-d) for 0-15 days. Conditions: (a) -20 °C, in dark; (b) 6 °C, in dark; (c) 22 °C, in dark; (d) 22 °C, under daylight.

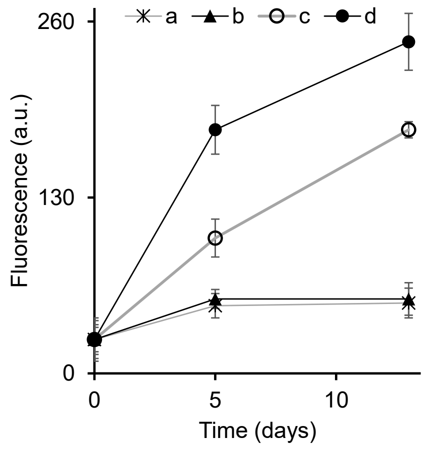

Figure **S24C**. Analysis of samples of solution of **2** in DMSO (10 mM) by using fluorescence spectroscopy: λ_{exc} = 300 nm, λ_{em} = 445 nm. The solutions were stored at different conditions (a-d) for 0-15 days. Conditions: (a) -20 °C, in dark; (b) 6 °C, in dark; (c) 22 °C, in dark; (d) 22 °C, under daylight.

Discussion of the results and conclusions: TLC of freshly prepared DCM solutions of the prodrug (samples *a*-*d* labelled “0”, Figure S24A) are practically not distinguishable from TLC’s of the solid samples kept at -20 °C for up to 15 days (samples *a* labelled “5 days” and “15 days”, Figure 24A) as well as TLC’s of the solid samples kept at 22 °C without protection from day light (samples *d* labelled “5 days” and “15 days”, Figure 24A). Also samples kept at 6 °C and 22 °C in dark are >90 % intact after their storage for 15 days (samples *b* and *c* labelled “5 days” and “15 days”, Figure 24A). However, in the latter case an additional more polar compound at the short retention time was observed. According to mass spectrometric analysis, this compound corresponds to the prodrug, which has lost pinacol: **2**-pinacol. Importantly, in none of the sample the fluorescent spot of HO-cpt is observed (the reference HO-cpt is shown in Figure 24A, left TLC plate) indicating that the ester E group in the prodrug is stable in the solid state.

DMSO solutions of the prodrug (10 mM) exhibit somewhat lower stability than the solid samples (Figure S24B). In particular, only freshly prepared samples (labelled “0”) do not contain the fluorescent spot. In contrast, the samples stored at all selected conditions (*a*-*d*) contain some limited amounts of HO-cpt, which is especially pronounced for the sample *d* stored for 15 days at 22 °C under daylight. Since the spots of HO-cpt and the prodrug overlap with each other, exact quantification of the degree of prodrug decomposition was not possible by TLC. Therefore, we used fluorescence spectroscopy to estimate the stability of the DMSO solutions (Figure 24C). The fluorescence spectroscopy is suitable for this purpose, since the prodrug is not fluorescent, where its expected decomposition products (e.g. HO-cpt) are fluorescent. Therefore, the fluorescence increase should correlate with decomposition degree of the prodrug. We confirmed that the DMSO solution of the prodrug (10 mM) is rather stable when stored at -20 °C in dark (*a*) and at 6 °C in dark (*b*), whereas storage at 22 °C in dark (*c*) and under daylight (*d*) lead to extensive decomposition (Figure 24C). Since even at the best storage conditions *a* and *b* some amount of the fluorescent products is formed after 5 and 15 days storage, we have used only freshly prepared DMSO solutions of the prodrug for all experiments described in this paper.

n-Octanol/water partition coefficient (LogP) of the prodrug and controls

Reversed-phase (RP)-TLC plates from Macherey-Nagel (Germany, Alugram Aluminiumfolien RP-18 W/UV_{254}, stationary phase thickness: 0.15 mm) were used for determination of LogP values. Mixtures of aqueous 3-(N-morpholino)propanesulfonic acid buffer (MOPS, 100 mM, pH 7.4) and acetonitrile (3/2 and 1/1, in both cases: v/v) were used as eluents. Benzyl alcohol (LogP= 1.1), 1-naphthol (LogP= 2.85), benzophenone (LogP= 3.18), anthracene (LogP= 4.45) and perylene (LogP= 6.25) were used as reference compounds with known LogP values. Freshly prepared solutions of the samples and references (in acetone) were spotted on the RP-TLC plates. The solvent migration distance was L= 4.5 cm. The spots of the compounds were monitored by using UV-imaging. Each spot was marked and its position relative to the start line (L_c) was measured. R_f values were calculated as ratio L_c / L. Calibration plots of R_f *versus* logP's for the known compounds were used for determination of logP values of new compounds. For boronic ester derivatives, two spots were observed. The upper spot corresponds to boronic acid pinacol ester and the lower spot – to boronic acid. LogP values of both species were calculated. All measurements were done in triplicates (Table S1).

Table **S1**. LogP values of prodrug **2** and controls.

Prodrug / control	logP
**2**	4.27 ± 0.04
**2**-pinacol	2.53 ± 0.06
**3**	5.39 ± 0.07
**4**	5.62 ± 0.07
**4**-pinacol	3.56 ± 0.06
HO-cpt	2.33 ± 0.07[i]

[i] Previously determined logP value for camptothecin is 1.82.[S5]

Determination of solubility of the prodrug 2 and controls in aqueous solutions

Prodrug **2** (a control) was dissolved in DMSO at concentrations of 10 mM, 5 mM, 2 mM, 1.5 mM, 1 mM and 0.2 mM. These solutions (10 µL) were transferred to quartz cuvettes containing either Dulbecco's phosphate-buffered saline (DPBS, 990 µL) or Roswell Park

Memorial Institute (RPMI) 1640 Medium and mixed thoroughly at 22 °C to obtain solutions/suspensions of compounds at final concentrations of 100 µM, 50 µM, 20 µM, 15 µM, 10 and 2 µM. The absorbance at 789 nm ($A_{789\ nm}$) of the resulting mixtures was measured at ambient temperature (22 ± 2 ^oC) and plotted as a function of the concentration of the compounds. Mixtures exhibiting $A_{789\ nm}$ values less than 2 times of $A_{789\ nm}$ observed for the control (true) solution containing DMSO (10 µL) and the above defined medium (990 µL) appeared transparent and were considered as true solutions.

Table **S2**. Solubility of compounds in DPBS and RPMI 1640 medium.

Prodrug / control	Solubility (µM)	
	DPBS	RPMI 1640 medium
**2**	at least 30	at least 30
**3**	at least 30	at least 30
**4**	at least 30	at least 30
**5**	at least 50	at least 50
HO-cpt	at least 5	at least 5

UV-visible spectra of the prodrug 2 and intermediate 2c*H⁺

UV-visible spectra of the compounds (5 µM, 10 µM, 15 µM, 20 µM, 25 µM) in triethylammonium acetate (TEAA) buffer (150 mM, pH 8) containing acetonitrile (15 % v/v) and DMSO (1 %, v/v) were recorded (Figure 3A-D, main text).

Dynamic light scattering (DLS) of solutions of the prodrug 2 and 2c*H⁺

DLS of solutions of the compounds (5 µM, 10 µM, 15 µM, 20 µM, 25 µM) in TEAA buffer (150 mM, pH 8) containing acetonitrile (15 % v/v) and DMSO (1 %, v/v) were determined under the following settings: 25 °C, solvent: ACN/water (RI: 1.330; viscosity (cP): 0.9200); analysis: (RI: 1.45; material absorption: 0.220); backward scatter; 3 measurements per sample). The data obtained are provided in Figures S25 and 3D, main text.

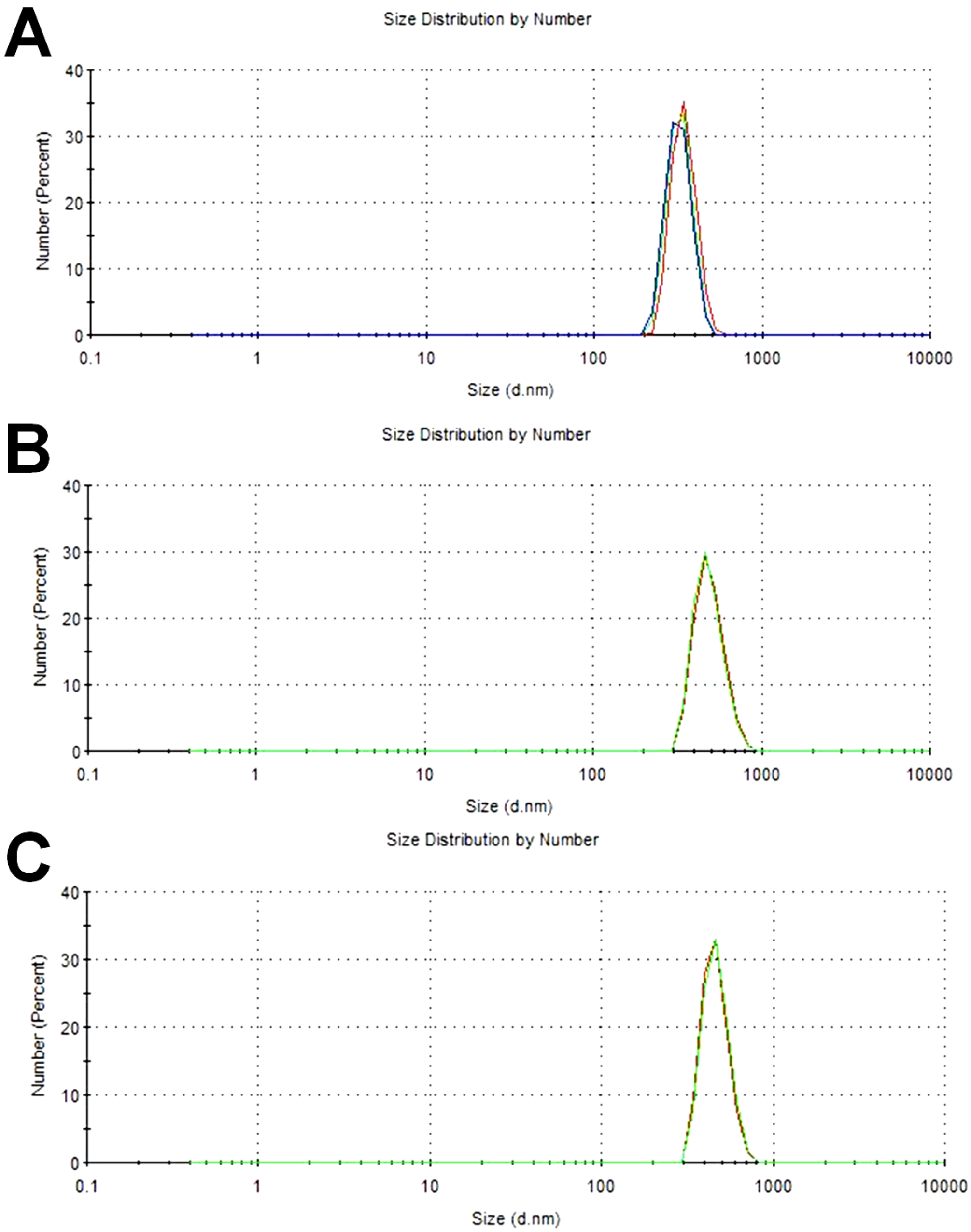

Figure **S25**. Exemplifying DLS raw data of the prodrug **2** in triethylammonium acetate (TEAA) buffer (150 mM, pH 8, CH_3CN 15 % v/v, DMSO 1%, v/v) at concentrations 5 (**A**), 10 (**B**), 15 μM (**C**). 2 or 3 measurements were performed for each experiment. Two independent experiments were performed. The mean size of the aggregates was used to calculate plot shown in Figure 3D (main text).

Stability of the prodrug 2 and control 3 at different pH's in the presence and absence of H_2O_2 monitored by fluorescence spectroscopy

Prodrug **2** and control **3** (20 μM) were dissolved in TEAA buffer (pH= 6; 7; 8; 10, 2 % DMSO (v/v), 200 μL/sample) and fluorescence spectra were acquired ($\lambda_{excitation}$= 365 nm, $\lambda_{emision}$= 400 nm) with a Thermo Scientific™ Varioskan™ LUX in black 96-well plates every 5 min. H_2O_2 (10 mM) was added after 15 min and the fluorescence intensity measurements continued for 4 h (Figure 4, main text, Figure S26A).

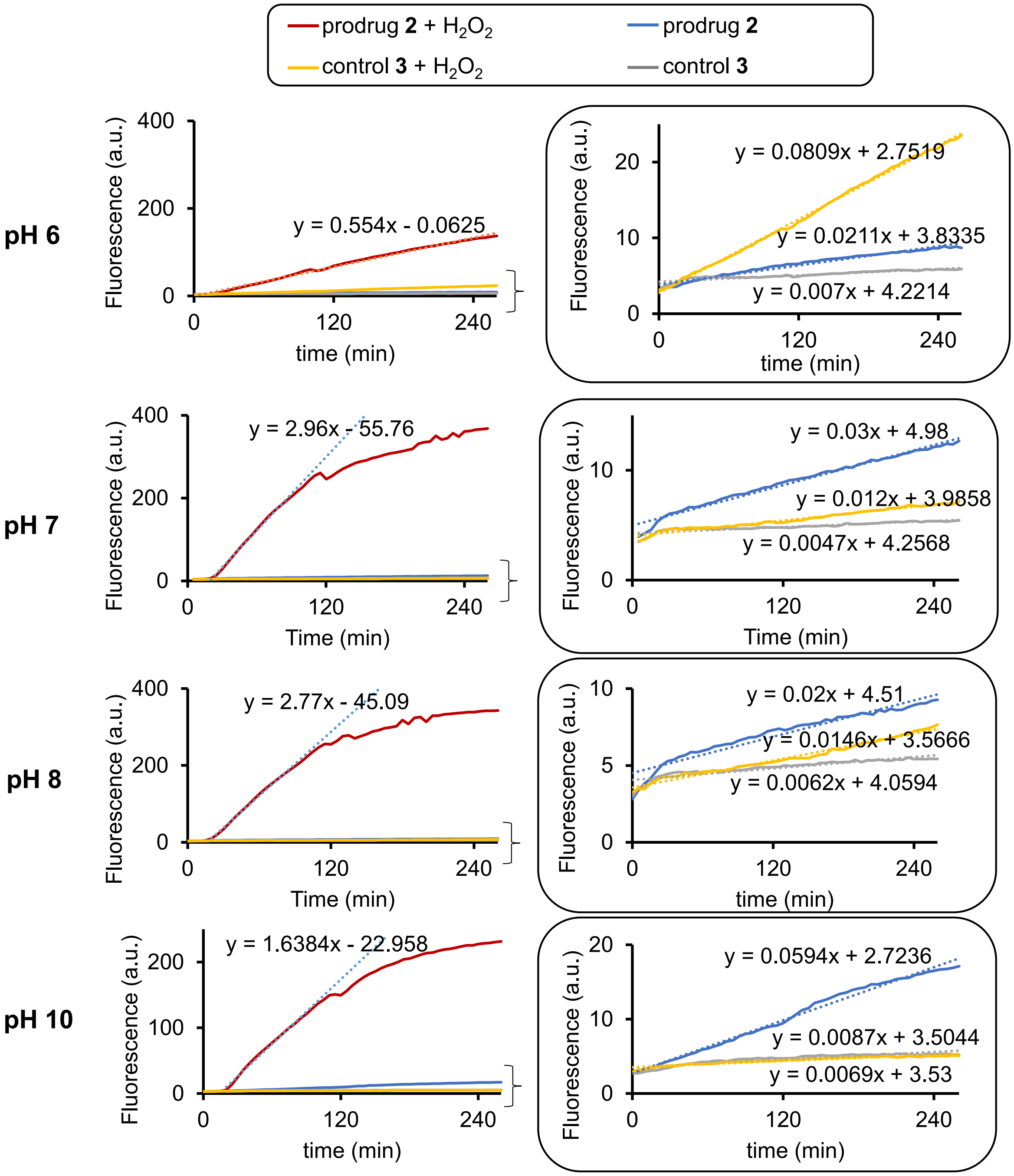

Figure **S26A**: Fluorescence increase ($\lambda_{excitation}$= 365 nm, $\lambda_{emision}$= 400 nm) observed in aqueous solutions of prodrug **2** and control **3** (20 µM) under different conditions. Linear fits of the initial (quickest) increase of the fluorescence is indicated with blue, dotted lines. Formula of the fitted curves are provided above these lines. These data were used to compile Tables S3 and S4.

The fluorescence of HO-cpt ($\lambda_{excitation}$= 365 nm, $\lambda_{emision}$= 400 nm) is decreased at higher pH's: F(pH 6) / F(pH 7) / F(pH 8) / F(pH 10) = 1 / 1 / 0.63 / 0.31 (Figure S26B).

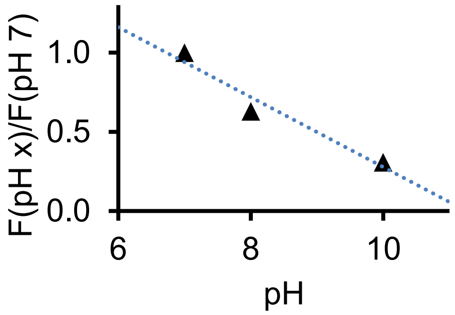

Figure **S26B**: Dependence of the normalized fluorescence (F(pH x)/F(pH 7)), where F(pH x) is the fluorescence of HO-cpt solutions (10 μM, $\lambda_{excitation}$= 365 nm, $\lambda_{emision}$= 400 nm) dissolved in TEAA buffers adjusted to different pH values (pH= 6, 7; 8 or 10) and containing 1 % DMSO, v/v).

To account for this difference, the values of $(dF/dt)_0$, corresponding to the linear fit of the initial (quickest) increase of the dependence of the fluorescence from time (expressed in min) were normalized by the corresponding factors: $f_{pH\ 6}$=1, $f_{pH\ 7}$=1, $f_{pH\ 8}$= 0.63, $f_{pH\ 10}$= 0.31 (Tables S3, S4).

Table **S3**. Normalized rates of the fluorescence increase of aqueous solutions of prodrug $FcN^{PG2/PG1}$-L-E-cpt in aqueous solutions at different conditions.[i]

Prodrug / control	$(dF/dt)_0$ / f_{pH} (a.u./min)[ii]			
	pH 6	pH 7	pH 8	pH 10
prodrug / - H_2O_2	0.021	0.030	0.032	0.190
prodrug / + H_2O_2	0.550	2.960	4.397	5.290
**Ratio of "prodrug / + H_2O_2 " / "prodrug - H_2O_2"**	**26.1**	**98.7**	**138.5**	**27.6**

[i] Conditions are provided in the description above.

[ii] The normalization is explained in the description above.

Table **S4**. Normalized rates of the fluorescence increase of aqueous solutions of control FcN^F-L-E-cpt in aqueous solutions at different conditions.[i]

Prodrug / control	$(dF/dt)_0$ / f_{pH} (a.u./min)[ii]			
	pH 6	pH 7	pH 8	pH 10
control / - H_2O_2	0.007	0.005	0.010	0.0022
control / + H_2O_2	0.081	0.012	0.024	0.028
**Ratio of "control / + H_2O_2 " / "control - H_2O_2"**	**11.6**	**2.6**	**2.4**	**1.3**

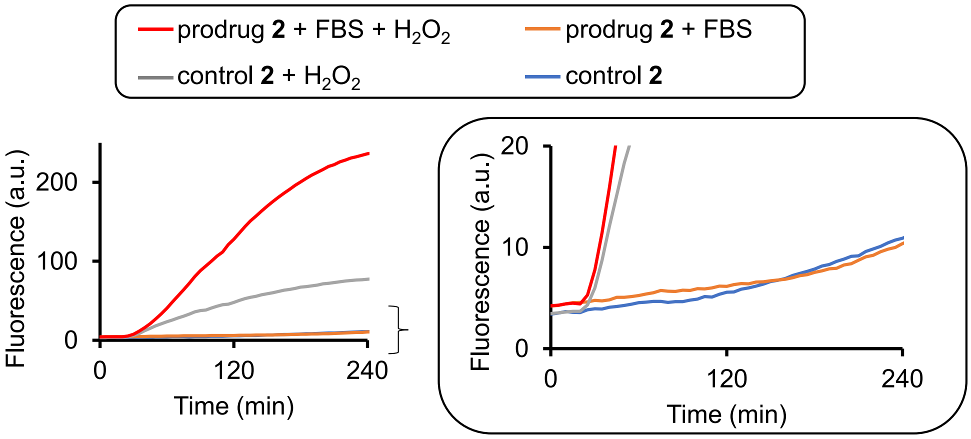

Figure **S26C**: Fluorescence increase ($\lambda_{excitation}$= 365 nm, $\lambda_{emision}$= 400 nm) observed in solutions of prodrug **2** (20 µM) in RPMI 1640 medium containing either H_2O_2 (10 mM, grey trace) or fetal bovine serum (FBS, 5 %, orange trace) or H_2O_2 and FBS together (red trace). A control containing only RPMI 1640 and 2 is shown as a blue trace.

Study of activation of the prodrug 2 and control 3 in the presence of different concentrations of H_2O_2 (0-10 mM)

Prodrug **2** and **3** (10 µM) were dissolved in PBS (10 mM, 150 mM NaCl pH=7.4 1 % DMSO (v/v), 200 µL/sample) and fluorescence spectra were acquired ($\lambda_{excitation}$= 365 nm, $\lambda_{emision}$= 400 nm) using a Thermo Scientrific™ Varioskan™ LUX in black 96-well plates every 5 min. After 50 min, H_2O_2 (10 mM, 5 mM, 1 mM, 500 µM, 100 µM, 50 µM, 10 µM, 0 µM) was added and the fluorescence intensity measurements continued every 5 min for 4 h (Figures 4A, B, main text of the paper).

Monitoring release of iron ions from the prodrug 2

Prodrug **2** (20 µM) was dissolved in TEAA buffer (150 mM, pH 7, 8) containing DMSO (1 %, v/v) and H_2O_2 (10 mM) and incubated for 2 h at 22 °C. Subsequently, all lypophilic intermediates were extracted 3 times using ethylacetate. Ferrozine (10 mM, in 0.1 M ammonium acetate, 100 µL) was added to the aqueous phase (1 mL) and the absorption at 450-650 nm measured to determine the Fe^{2+} concentration. To reduce Fe^{3+} to Fe^{2+} hydroxylamine solution (1.4 M hydroxylamine hydrochloride in 2 M HCl, 150 µL) was added. After 10 min incubation, ammonium acetate buffer (10 M in water, pH 9.5, 50 µL) was added and the absorption re-measured (Figure 4D, main text of the paper).

Monitoring the reaction of the prodrug 2 with H_2O_2 by using HPLC

Prodrug **2** (20 µM) was dissolved in TEAA buffer (150 mM, pH 7, 8 or 10) containing acetonitrile (50 %, v/v), DMSO (1 %, v/v) and either H_2O_2 (10 mM) or without it. The resulting mixture was gently shaken for 0, 1 and 2 h. The samples (20 µL) were analyzed by HPLC. Separation conditions: column - Macherey–Nagel EC HPLC NUCLEODUR C18 HTec, particle size 5 µm of the stationary phase, column size: 50 x 2 mm, the column was coupled to a UV-visible detector (detection of absorbance at 370 nm: for **2**) and to a quadrupole MS detector (detection of total positive ion current); gradient of solvent B in solvent A was used (solvent A: ammonium formiate in water (10 mM) containing formic acid (0.1 %, v/v); solvent B: CH_3CN containing formic acid (0.1 %, v/v)); starting conditions – 15 % B, then from 15 to 95 % B in 11 min, followed by 4 min at 95 % B. The data obtained are provided in Figures 5A-C. Structures of intermediates and products identified in these experiments are provided in Figure 27.

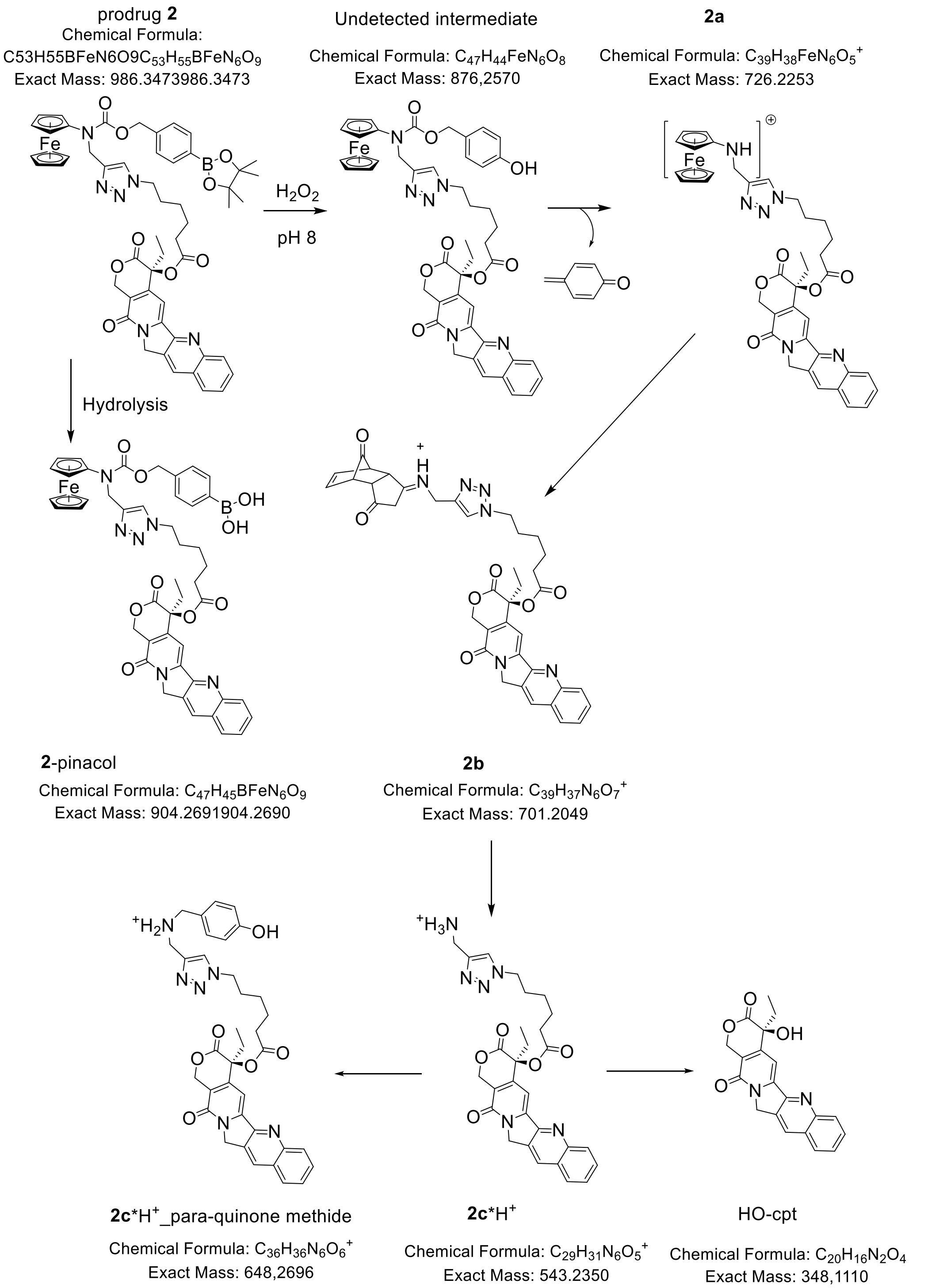

Figure **S27**. Structures, brutto formulas and exact masses of all identified and assumed intermediates released from the prodrug **2**.

Preparation of intermediate 2c*H+

To confirm the release of **2c***H+ from the prodrug, we prepared the former compound by chemical synthesis. Next, we compared retention times of both chemically prepared compound and the compound released from the prodrug in the presence of H_2O_2. We found that both compounds exhibit the same retention factors, thereby confirming the chemical identity of the stable intermediate.

2c*H+ Intermediate **S2** (1 eq) was mixed with 3-N-(t-butyloxycarbonyl)aminopropin[S3] (1 eq, 0.02 M in DMF). To the reaction mixture DIPEA (3 eq), CuI (0.1 eq) and TBTA (0.12 eq) were added. The reaction was stirred overnight. The solvent was removed *in vacuo* (0.01 mbar) and the product (**S8**) was purified by column chromatography (silica gel, DCM/EtOAc-gradient from 1/1 (v/v) to 3/7 (v/v)). Next, **S8** was dissolved in EtOAc (1 mL), HCl (1 mL, 4 M in dioxane) was added and the mixture was stirred for 15 min. The solvent was removed *in vacuo* (0.01 mbar). The product was washed twice with DCM to yield a yellow solid at 23 % yield. The purity of the compound was confirmed by HPLC (Figure S28) and its identity by mass spectrometry: found *m/z* 543 Da, calcld $C_{29}H_{31}N_6O_5^+$ ([M]+) 543 Da.

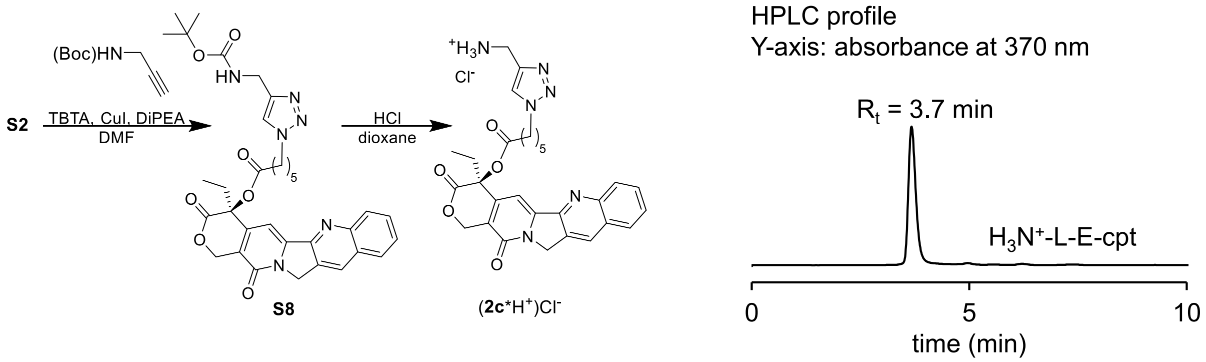

Figure **S28**. An approach used for synthesis of (**2c***H+)Cl and an HPLC profile of pure (**2c***H+)Cl.

Monitoring HO-cpt release from the prodrug 2 by using HPLC coupled to the mass detector

The samples obtained after incubation of the prodrug with H_2O_2, as described above, were analyzed by liquid chromatography hyphenated quadrupole−time-of-flight mass

spectrometry (LC-QTOF). For this, an UltiMate 3000 RS liquid chromatography system (degasser, binary nano flow pump, autosampler, column compartment; ThermoFisher, Dreiech, Germany) was coupled to a timsTOF Pro mass spectrometer (Bruker Daltonik, Bremen, Germany) equipped with an electrospray ionization source. The source parameters were set as follows: capillary voltage 4200V, dry gas 3.5 bar, and temperature 200 °C. The mass spectrometer was run in positive operation mode. An HSS T3 column (50 mm length, 300 µm inner diameter, 1.8 µm particle size; Waters, Eschborn, Germany) was operated at 35 °C and a flow rate of 15 µl/min. Gradient elution (eluent A: 10 mM ammonium formate pH 7, eluent B: 1% formic acid in acetonitrile) was applied. The eluent composition was changed as follows: time [min]/%B: 0.0/15, 0.5/15, 11.0/95, 15.0/95, 15.5/15, 25.0/15. Data evaluation was carried out at *m/z* 349 Da (protonated HO-cpt: $[M+H^+]^+$)) and *m/z* 543 Da (intermediate **2c***H^+: $[M]^+$). Under these conditions HO-cpt (R_t = 4.4 min) and **2c***H^+ (R_t = 4.5 min) have distinguishable mobility (Figure S29). The data obtained are provided in Figure 5D, E in the main text.

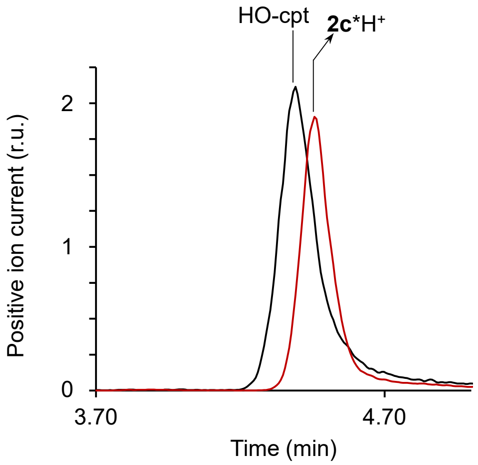

Figure **S29**. HPLC-MS traces of HO-cpt (black line, monitoring *m/z* 349 Da: $[M+H^+]^+$) and H_3N^+-L-E-cpt (red line, monitoring *m/z* 543 Da: $[M]^+$).

Monitoring the reaction of 4 with H_2O_2 by using HPLC

Prodrug **4** (20 µM) was analyzed by HPLC-MS analogously to **2** (see the protocol above, Figure 30A).

Sensitivity of detection of HO-dmb by HPLC-MS was not sufficient as confirmed by experimenting with pure HO-dmb. We observed that GC-MS system can detect HO-dmb accurately. Therefore, GC-MS was selected for monitoring the release of this product from prodrug **4** (Figure 30B).

4 (20 µM) was dissolved TEAA buffer: 1 mL, 150 mM, pH= 7 or 8, 15% CH_3CN, v/v, with or without H_2O_2 (10 mM). The samples were incubated for 1 h, 8 h or 24 h. Subsequently, GC/MS measurements were performed with a GC-2010 Plus gas chromatography system (Shimadzu) with helium as carrier gas and detection by a QP2010 SE single quadrupole mass spectrometer (Shimadzu). A Supelco® EquityTM-5 Capillary Column with the dimensions 30 m x 0.25 mm and a film thickness of 0.25 µm was used. A temperature gradient of 40 °C for 1 min, to 220 °C in 5.5 min and subsequently for 2 min at 220 °C was used, with a total flow of 27.6 mL/min and a column flow of 1.96 mL/min. The total ion current was detected starting from 3.75 min. The data obtained are provided in Figures 29A and B. Structures of intermediates and products identified in these experiments are provided in Figure 30C.

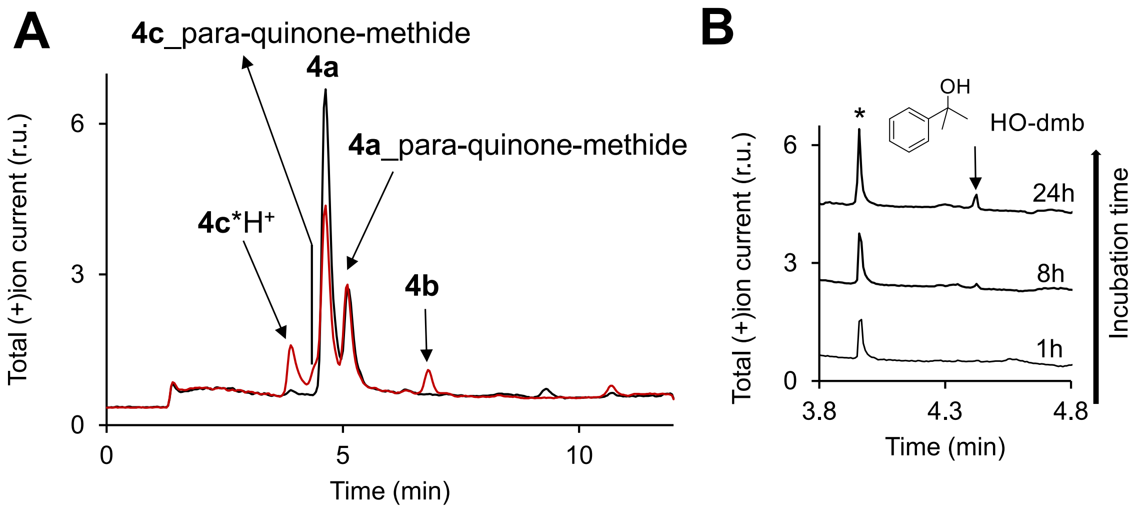

Figure **S30**. **A**: HPLC-MS profiles of prodrug **4** (20 µM) incubated with H_2O_2 (10 mM) for 60 min either at pH 7 (black colored trace) or pH 8 (red colored trace). The MS detector was turned on at 2 min (indicated on the plot by the signal increase) after all buffer components were eluted. **B**: GS-MS profiles of **4** (20 µM) incubated with H_2O_2 (10 mM) for 1-24 h. The peak indicated with * is a non-identified ion derived from **4**. For both **A** and **B**: Other experimental conditions are given in the experimental descriptions provided

above. Intensities of the peaks (Y-axis) correspond to the total current of positively charged ions. “r.u.”= relative units.

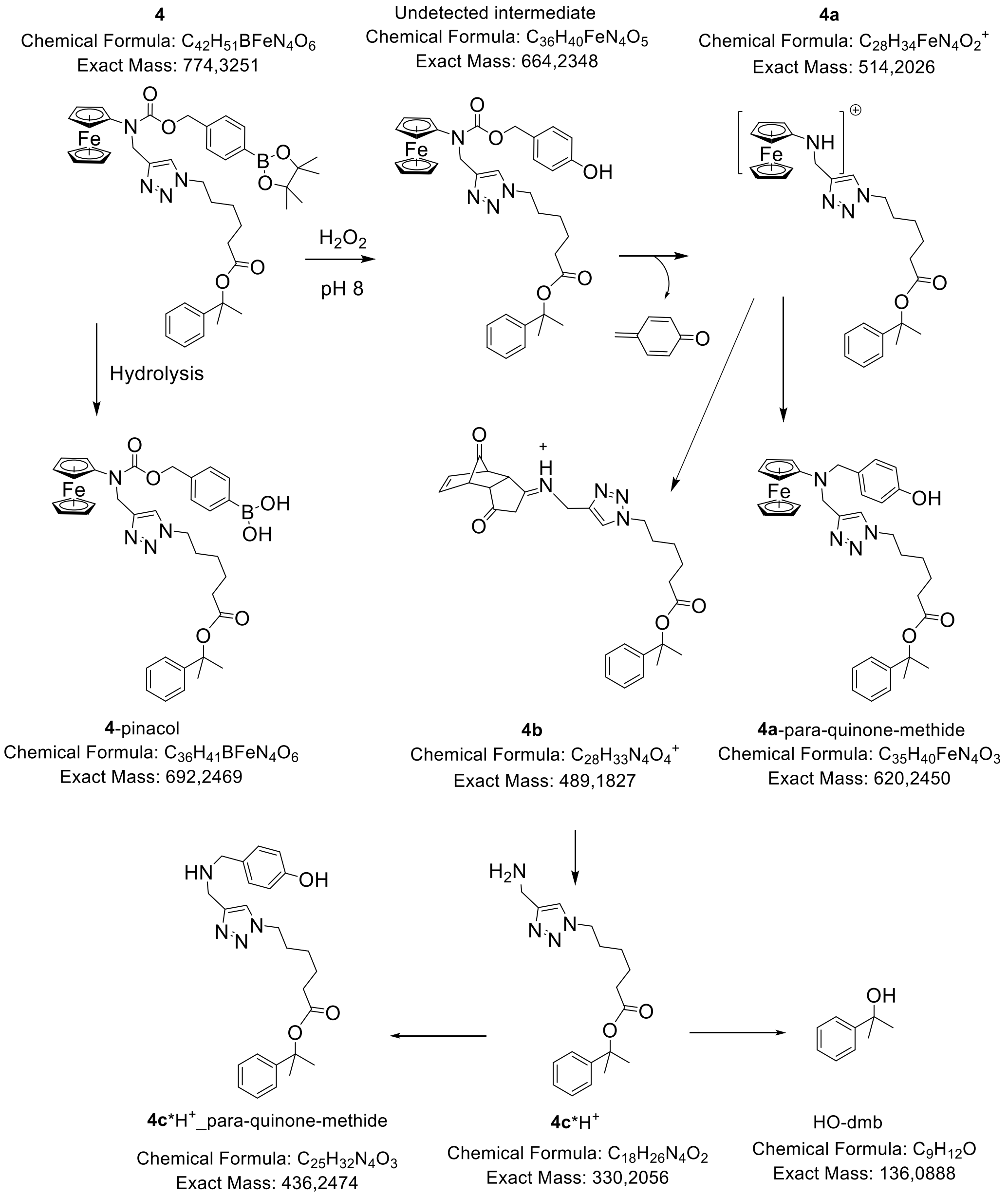

Figure **S30C**. Structures, brutto formulas and exact masses of all identified and assumed intermediates released from **4**.

Theoretical studies

Modelling of intermediate **2c***H$^+$ in aqueous solution by using molecular dynamics (MD)

We conducted molecular dynamics (MD) simulations to find out whether any of the intramolecular interactions will play any role.

MD simulations were performed with Amber 20[S6] pmemd.cuda, using Gaff[S7,S8] 1.81 with RESP[S9,S10] charges based on calculations with Gaussian 16[S11] (HF/6-31G*//B3LYP/6-31G*;[S12-S22] optimizations were performed in polarizable continuum model (PCM) water),[S23,S24] in agreement with the Amber force fields.[S9,S25] The solute was solvated with TIP3P[S26] water (truncated octahedral boxes exceeding the molecule dimensions by 20 Å in either direction)and one Cl^- was added to neutralize the system.[S27] After initial geometry optimization (first 5,000 steps with restraints (50 kcal mol^{-1} Å^{-2}) on the solute, then 5,000 optimization steps without restraints, switch from steepest descent to conjugate gradients after 500 steps in either case), the solvated system was heated to 310 K during a 500 ps simulation with weak restraints (10 kcal mol^{-1} Å^{-2}) on the solute in the NVT ensemble. After that, 1000 ns unrestrained NPT Langevin dynamics were performed at 310 K and 1 bar (weak pressure coupling, isotropic position scaling, pressure relaxation time 2 ps, collision frequency 2 ps^{-1}). SHAKE constraints were applied to bonds involving hydrogen.[S28] Periodic boundary conditions were used throughout and the distance cutoff for all nonbonding interactions was set to 10 Å. Long-range electrostatics were described by the particle-mesh Ewald method.[S29,S30] For van der Waals interactions beyond those included in the direct sum, a continuum model correction for energy and pressure was used, as implemented in Amber. Coordinates were saved every 10 ps. Three independent runs were performed. Only the last 900 ns were used for analysis. Cpptraj[S31] from the AmberTools suite, vmd 1.9.3[S32] and custom-made Jupyter notebooks with Python and Matplotlib were used for further analyses.

As can be seen in the snapshots, the distribution of N1—C1 distances (N1: aliphatic amino group; C1: carbonyl carbon atom of the ester group) and the distribution of gyration radii (Figures S31A-C), **2c***H$^+$ exhibits two conformations, one - more stretched and one - more compact. It is interesting to note that even in the compact conformation, with a radius of gyration, R_{gyr}, below 7 Å, that is in a rather compact fold, there are no distances

between N1 and C1 atom below 4 Å. This renders interactions of the ammonium group at the carbonyl oxygen of the E unlikely. In the MD simulations, the ammonium group forms intramolecular hydrogen bonds with the carbonyl oxygen of E with a negligible probability.

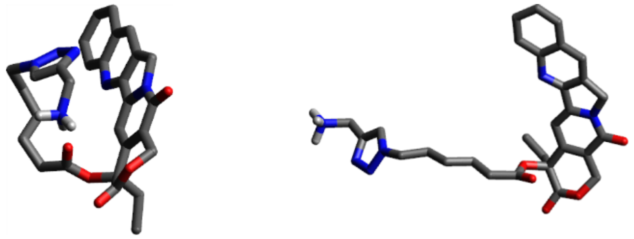

Figure **S31A**. Snapshots of the MD simulations of **2c***H$^+$.

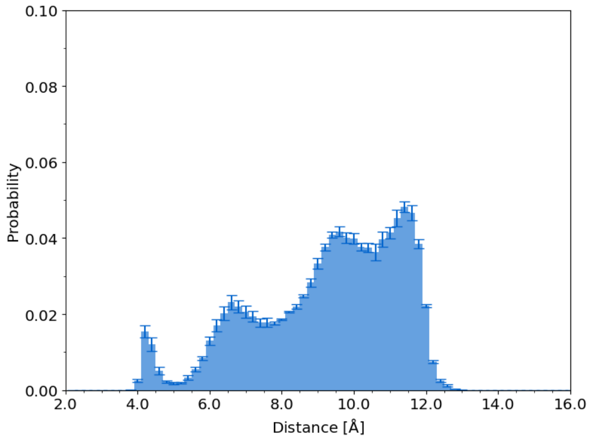

Figure **S31B**. Distribution of distances between the N-atom of the amino group (N1) and the ester carbon atom (C1) of **2c***H$^+$.

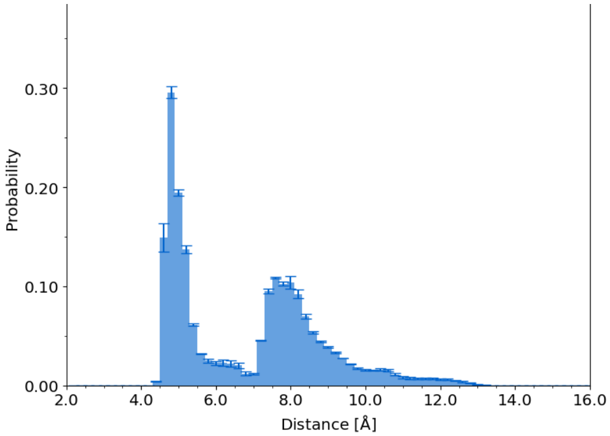

Figure **S31C**. Radius of gyration of **2c***H$^+$ as a measure of compactness.

The radial distribution function of water molecules around C1 shows a peak at around 4 Å, similar to the observed N1-C1 distances (Figure S31D). These data suggest attack by one of those water molecules (or OH^- ions) on the carbonyl group (C1=O1) to be feasible.

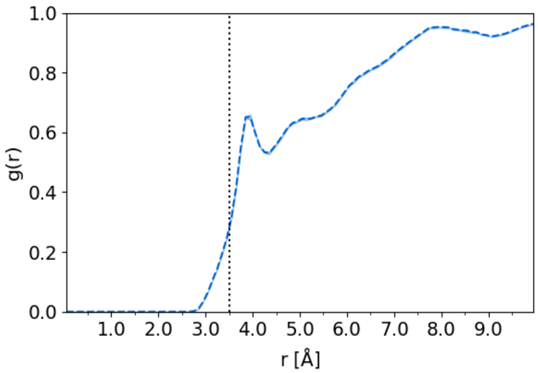

Figure **31D**. Radial distribution function, g(r) of water molecules around the ester carbon atom (C1) of H_3N^+-L-E-cpt. The dashed line indicates a hydrogen-bond distance.

Reaction calculations in explicit water (Figures 31E, F)

A snapshot of the molecular dynamics simulations of **2c***H^+ in its folded conformation, that is with the amino group close to the carbonyl oxygen atom (O1) of the ester group, including all water molecules, was taken to model the hydrolysis reactions. The water molecule closest to the ester group was changed into an OH^- ion by deleting one hydrogen atom. To preserve charge neutrality of the total system, the Cl^- ion far from the solute was deleted as well. This structure was then optimized using a combined quantum mechanical/molecular mechanical energy function in which the **2c***H^+ and the OH^--ion were treated quantum mechanically (with PM3[33]) and the water molecules with the TIP3 force field also used in the classical MD simulations (see above). Only the water molecules which were within 15 Å of the ester carbon atom (C1) were allowed to move, all other water molecules were kept stationary.

From the optimized reactant, we generated a tetrahedral intermediate by restraining the C1-OH distance from 3.7 to 1.4 Å in 0.1 Å steps and relaxing all other coordinate. The structure with the shortest C1-OH distance was optimized with the same criteria as the reactant state.

From this optimized tetrahedral intermediate, we generated a second intermediate and the final product by a two-dimensional scan, along the C1-O2 and H-O2 distances, respectively. The C1-O2 ester bond was varied in steps of 0.1 Å from 1.4 to 3.4 Å and the distance between the former hydroxyl H-atom and the O2 atom of the leaving HO-cpt was changed from 1.85 to 0.9 Å, also in steps of 0.1 Å. The minima on the corresponding potential energy surface were further optimized to verify that they correspond to the second intermediate after C1-O2 bond dissociation but before proton transfer and final product after proton transfer, respectively.

We then performed umbrella sampling simulations using the same reaction coordinates as for the scans and the structures from the constrained optimization therefrom. Accordingly, the nucleophilic attack was explored with 1-dimensional and the subsequent steps with 2-dimensional umbrella sampling. We used a harmonic biasing potential with a force constant of 1000 kcal/mol/Å ^{2} in the equilibration phase of the simulations. The equilibration phase consisted of heating from 0 to 300K over 3000 steps with velocitiy rescaling, 10ps of equilibration at 300K with a Nosé -Hoover Langevin thermostat and further 10 ps of equilibration in an NVT ensemble. The production phase was 100ps per window in the one-dimensional and 50ps in the two-dimensional umbrella sampling simulations, respectively. We used the leap-frog integrator with a 1fs time step and the values of the reaction coordinates were monitored for all time steps.

We performed an additional set (three runs) of umbrella sampling simulations for the ester hydrolysis in which the distance between the amino group N-atom (N1) and the ester carbonyl oxygen atom (O1) was restrained with a harmonic potential (distance 3.3 Å and force constant 1000 kcal/mol/Å^{2}).

All QM/MM calculations were performed with the CHARMM[34] programme. The one- and two-dimensional free energy profiles were computed using WAHM.[35]

All umbrella sampling simulations were repeated three times. The reported free energies are the mean of the three independent simulation runs and errors are estimated as the standard error from this mean.

On the two-dimensional free energy surface, the minimum free energy path was determined by converting the discretized free energy surface into a weighted graph with the nodes representing the grid points on the free energy surface and the edge weights are the larger of the two energy values associated with the two nodes connected by an edge. On this graph, the shortest path between the free energy minima (tetrahedral intermediate, second intermediate, and final product), were computed using the Dijkstra algorithm.[36] These calculations were done using python[37] and the corresponding plots were generated using matplotlib.[38]

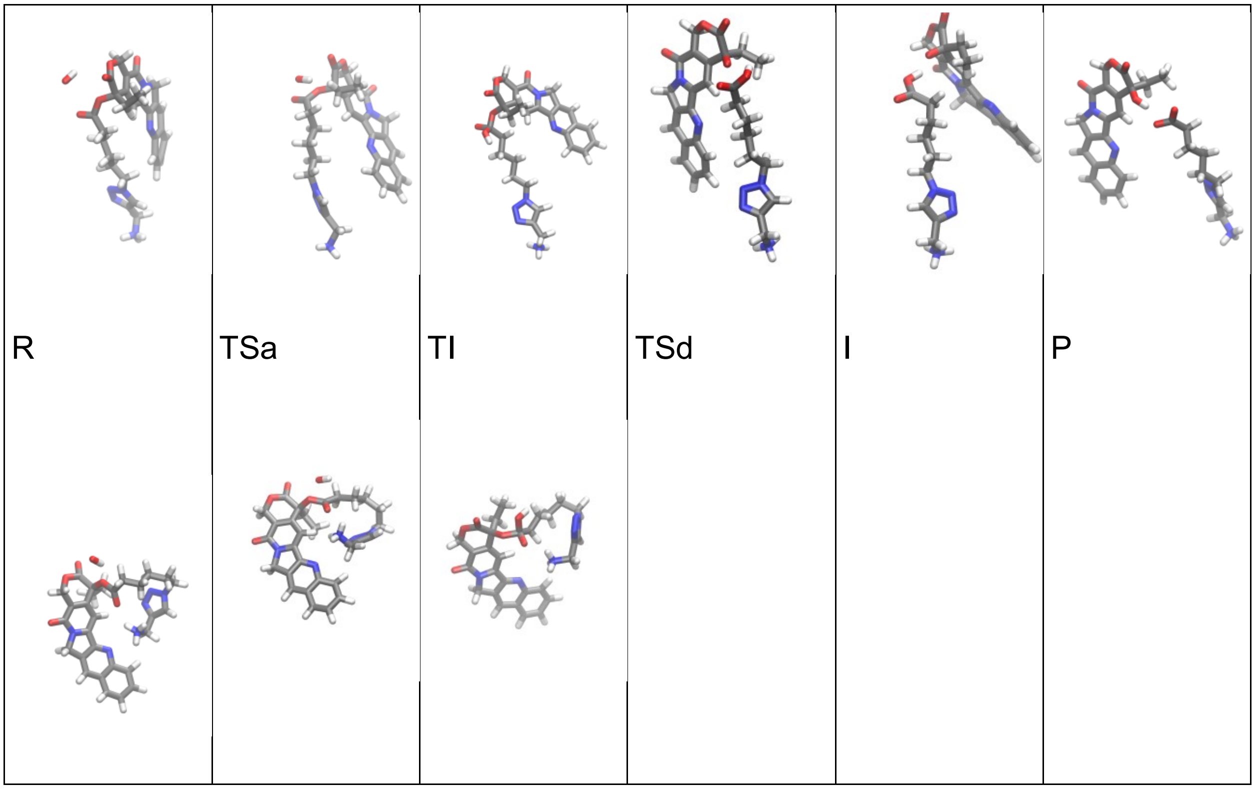

Figure **S31E**: Snapshots of the umbrella sampling simulations, representing (top) reactant (R), transition state of nucleophilic attack (TSa), tetrahedral intermediate (TI), cleaved intermediate (I), transition state of the leaving group departure (TSd), and product (P) of the ester hydrolysis and (bottom) reactant, TSa, and TI with constraint of the C1-N1 distance (see methods). For clarity, the solvent water is not shown.

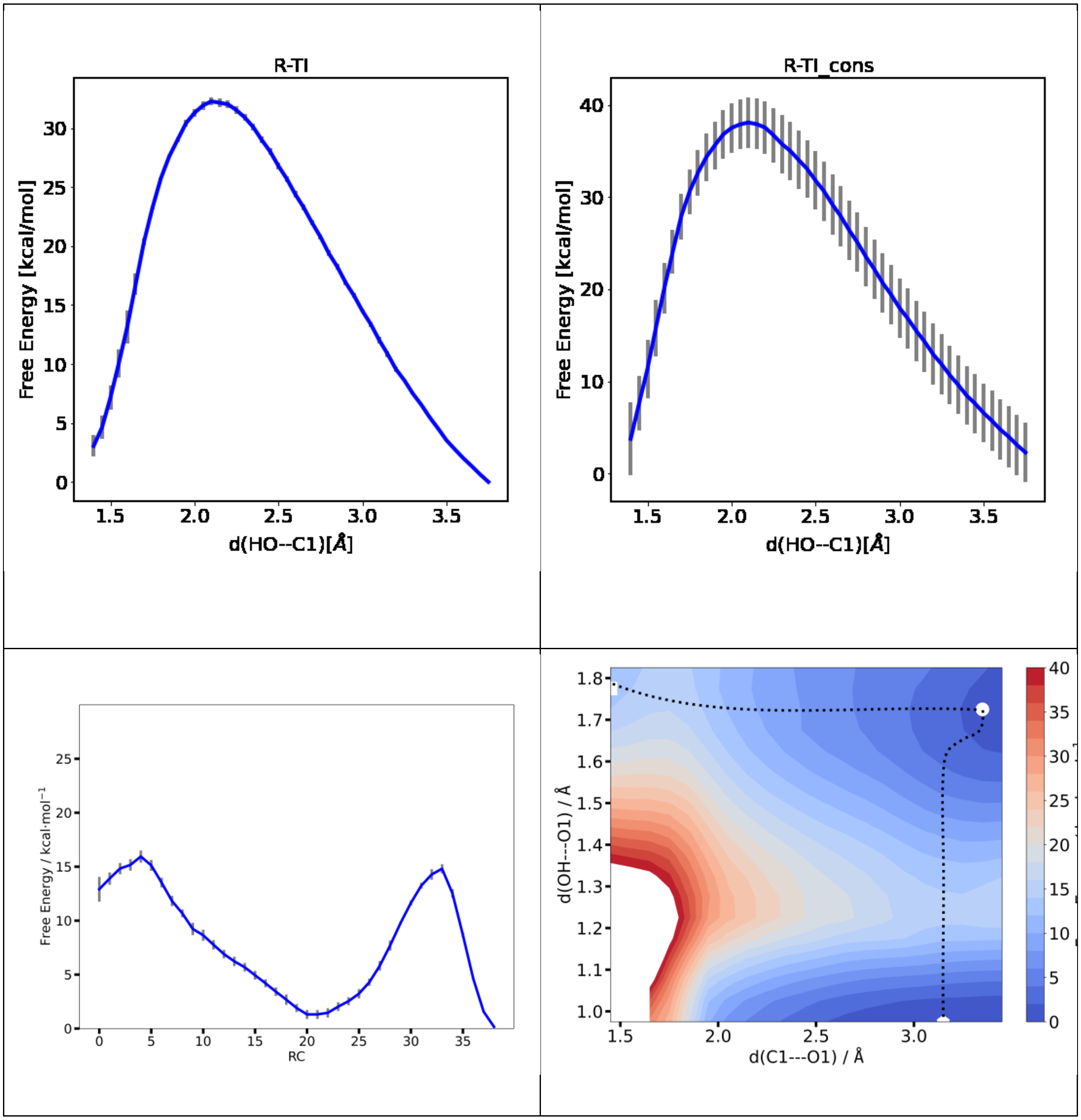

Figure **S31F**: Free energy profiles of the ester hydrolysis. Formation of the tetrahedral intermediate has been computed by one-dimensional (top), final product formation by two-dimensional umbrella sampling simulations (bottom) along the reaction coordinates indicated. Formation of the tetrahedral intermediate has, moreover been calculated with a constrained between C1 and N1 atoms, so as to force a hydrogen bond between the terminal amino group and the carbonyl oxygen atom (top right).

Cell culture experiments

Cells and cell culture

Cancer cells were obtained from the following sources: BL-2 Burkitt lymphoma cells - DSMZ (Germany); A2780 human ovarian and DU-145 human prostate cancer cell lines - Sigma-Aldrich; SAS human head and neck squamous cell carcinoma (HNSCC) cell line - HSRRB (Japan); FaDu HNSCC cell line representing a Dresden subline of FaDu-ATCC HTB-43 cells as emphasized earlier.[S39] Furthermore, the following normal cell types were included in our study: ARPE-19 human retinal epithelial cell line – ATCC (USA); commercial normal human dermal fibroblasts (NHDF) – PromoCell, kindly provided by the collaborating laboratory of Prof. Dr. C. Alexiou, SEON, Friedrich-Alexander-University Hospital Erlangen), normal human foreskin fibroblast (HF) - established by the group of Prof. Dr. L. Kunz-Schughart and used at low passage as previously described,[S40] normal human skin fibroblasts (SBLF9) – courtesy of the group of PD Dr. B. Frey, Chair of Radiation Therapy, University Hospital Erlangen; Germany.

The cells were cultured according to recommendations of the Deutsche Sammlung von Mikroorganismen und Zellkulturen GmbH (DSMZ, ACC 625), ATCC (HT-81) and ECACC (93112519) and/or adapted to low glucose (1 g/l) Dulbecco's Modified Eagle's Medium containing 20 mM HEPES and supplemented with 10 % FBS and antibiotics as described elsewhere.[S39] Suspension BL-2 cells were grown to (0.5−1.5) × 10^6 cells/mL and diluted as required. Adherent A2780, DU-145, SAS, FaDu, ARPE-19, NHDF, HF and SBLF9 cells were cultured to 70−80% confluence. For passaging and experimental setup, the adherent cells were detached from culture flasks by using a mixture of trypsin (0.025 % (w/v) for A2780 cells and 0.05 % for all other cell lines) and 0.02 % (w/v) N,N,N’,N’-ethylenediamine tetracetic acid (EDTA) in PBS buffer.

Determination of the viability of A2780, BL-2, DU-145 and NHDF cells

Suspension BL-2 cells were centrifuged, the medium was removed and the cells were washed two times with PBS and resuspended in RPMI 1640 medium containing 10% FBS, 1% L-glutamine, and 1% penicillin/ streptomycin. Adherent A2780, DU-145 and

NHDF cells were washed two times with PBS buffer, trypsinated, and re-suspended either in the RPMI 1640 medium or DMEM (for NHDF cell line) containing 10% FBS, 1% L-glutamine and 1% penicillin/streptomycin. The suspensions were spread in the wells of a 96-well microtiter plate containing typically 25.000 cells for 24 and 48 h and 12.500 cells for 72 and 96 h assays or 10.000 cells for experiments with NHDF cells per well per 100 µL. The suspensions were left standing at 37 °C in the chamber filled with CO_2 (5 %) overnight for adherent cell lines or immediately used for suspension cell line. Stock solutions of prodrugs of different concentrations (1 µL, solvent DMSO) were added to the wells and incubated for 24, 48 or 96h. Three experiments were conducted for each concentration of the prodrug. Finally, 3-(4,5-dimethylthiazol-2-yl)-2,5-diphenyltetrazolium bromide (MTT; 20 µL of the solution prepared by dissolving MTT (5 mg) in PBS buffer (1 mL)) was added to each well, incubated for 3 h, treated with sodium dodecyl sulfate (SDS) solution (90 µL, 10% solution in 0.01 M aqueous HCl), and incubated overnight. Then the intensity of absorbance at 590 nm was measured (MTT is converted to blue dye (λ_{max} = 590 nm) in live cells). The absorbance at 690 nm was taken as a baseline value. These data were applied to calculate the relative number of viable cells (Tables S5 and S6).

Table **S5**. Effects of prodrugs and controls on the viability of human Burkitt's lymphoma BL-2 cells and human ovarian cancer A2780 cells.

Compound	Cells					
	BL-2			A2780		
Incubation time / h →	24	48	96	24	48	96
**2**	3 ± 1	2 ± 1	0.3 ± 0.1	>20	9 ± 3	1.0 ± 0.2
**3**	>20	7 ± 1	1.3 ± 0.2	>20	>20	8 ± 2
**5**	40 ± 4	32 ± 6	28 ± 5	43 ± 6	38 ± 4	34 ± 5
HO-cpt	0.07 ± 0.02	0.02 ± 0.01	0.02 ± 0.01	3 ± 1	0.08 ± 0.02	0.08 ± 0.02

Table **S6**. Effects of prodrugs and controls on the viability of human prostate cancer DU-145 cells and normal human dermal fibroblasts NHDF.

Compound	Cells

	DU-145			NHDF
Incubation time / h →	24	48	96	48
**2**	>20	>20	9 ± 2	>20
**3**	>20	>20	>20	>20
**5**	>50	>50	>50	>50
HO-cpt	-	-	0.03 ± 0.01	15 ± 3

Determination of the viability of SAS, FaDu, ARPE-19 and HF cells

The MTT assay was also applied to assess the impact of the prodrug and the control on the viability of the two human HNSCC cell lines SAS and FaDu compared to normal retinal pigment epithelial ARPE-19 cells and human fibroblasts (HF). Cells were seeded into 96-well plates at concentrations of 4000 and 2000 cells per well, respectively, for the 24 h and 48 h treatment intervals, and were pre-cultured for 24 h before exposure to the drugs. All drug dilutions were prepared from 20 mM stock solutions in DMSO. The final DMSO concentration was set to 0.5% in all samples including control. After completion of the treatment, the wells were processed according to the routine MTT protocol as described above. The formazan absorbance was measured on a Biotek Epoch Microplate Spectrophotometer at 590 nm and corrected for the background signal at 620 nm. N≥3 independent experiments were performed for each prodrug or control, cell type and treatment interval. Cell survival was recorded as function of the drug concentration, and dose-response curves were fitted with the Boltzmann equation where appropriate. Figure S32 documents the finding of the prodrug but not the control substance to reduce the survival of the tumor cells in a dose-dependent manner (middle vs. right panels) while not affecting the normal cell in the same concentration range (middle vs. left panels).

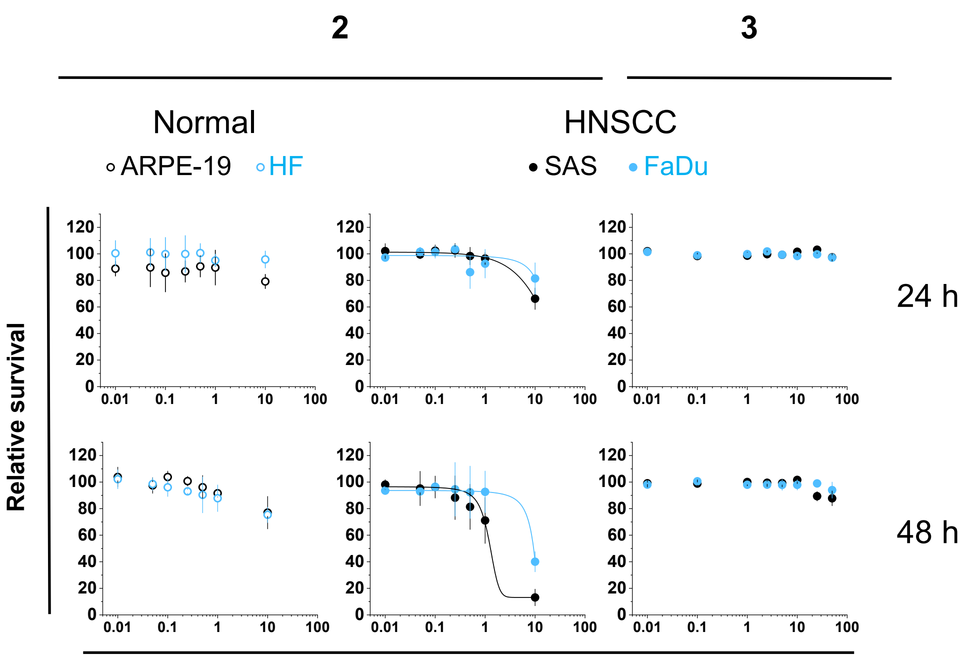

Figure **S32**. Effects of the ROS-responsive prodrug **2** on the viability of representative cancer (SAS, FaDu - middle panels) and normal cell types (ARPE-19, HF - left panels), compared to the impact of the ROS-resistant control **3** on SAS and FaDu cancer cell viability (right panels); the exposure times (24 or 48 h) are indicated on the right hand side of the diagrams.

<u>Clonogenic assay: testing effects of the prodrug and control on single cell colony formation capacity of cancer cells</u>

Exponentially growing SAS and FaDu cells were dissociated to prepare single cell suspensions in standard DMEM for seeding defined low cell numbers into 6-well plates (150-300 cells in 1 ml per well). After 8 h of incubation at 37” C in an 8% CO_2, humidified atmosphere to allow cell adherence, 50% of the supernatant was replaced by media containing the prodrug/control at 2x concentration. Treatment was terminated by medium exchange after 48 h of drug exposure under culture conditions. Since stock solutions of produgs/control substances in DMSO were diluted in supplemented medium for cell culture experiments, control cells were always handled accordingly by applying equivalent diluted DMSO concentration(s) for the same time period. Subsequently. SAS and FaDu cells were allowed to grow for an additional period of 7 and 10 days, respectively, allowing

for ≥5-6 cell divisions according to the cell line-specific culture doubling times, and were then fixed and stained to identify and count colonies of >50 cells. Plating efficiencies were caculated as the number of counted colonies relative to the number of seeded cells, and drug effects were assessed as clonogenic survival relative to the colony formation of control cells set to 1 (Table S7).

Table **S7.** Effects of the prodrug and the control on the capacity of SAS and FaDu cells to form colonies; data are given relative to untreated control cells (= relative clonogenic survival) and are documented as means ± SD from N=3-4 independent experiments.

Compound	Concentration (µM)	Relative clonogenic survival	
		SAS	FaDu
**2**	0.2	0.81 ± 0.07	0.77 ± 0.11
	0.5	0.65 ± 0.08	0.30 ± 0.09
**3**	0.2	0.90 ± 0.10	0.94 ± 0.16
	0.5	0.92 ± 0.13	0.76 ± 0.15

N≥3; Σ n≥9

Monitoring the uptake of the prodrug by BL-cells by quantification of intracellular fluorescence signal characteristic for camptothecin derivatives

BL-2 cells, grown in RPMI 1640 medium supplemented with 20% FCS, 1% glutamine, and 1% penicillin/streptomycin, were washed with phosphate buffered saline (PBS) 2 times and centrifuged. The medium was replaced with RPMI 1640 medium (5% FCS, 1% L-glutamine, 1% penicillin/streptomycin) to obtain a suspension containing 10^6 cells/mL. This suspension was spread in T75 cm^2 flasks (12 mL of cell suspension per each flask, 3 flasks for every condition). Solutions of drugs/prodrugs (120 µL in DMSO) were added to the suspensions and incubated for 1 h. The final concentration of the prodrugs in the suspensions was 1 µM. Incubation of BL-2 cells with these concentrations of drugs/prodrugs did not result in morphological changes of the cells. At the end of the treatment, the uptake was stopped by adding HCl solution to achieve the final HCl concentration of 0.01 M and the cells were centrifuged into pellets. The cell pellets were washed with ice-cold PBS (2 times) containing 0.01 M HCl and again centrifuged into

pellets. The pellets were vortexed in mixture (40 mL) of biograde water (32 mL), aqueous sodium hydroxide (2 mL, 10 M) and hydrogen peroxide (6 mL, 10 M) and incubated overnight at 37°C before counting. The fluorescence spectra (λ_{ex}= 365 and λ_{em}= 380 - 650 nm) of the obtained solutions were measured. The emission maximum (460 nm) was used to determine the concentration of HO-cpt in the mixture. Under these conditions ester groups in both the prodrug **2** and the control **3** are cleaved due to the hydrolysis by NaOH. Therefore, this method allows quantifying the total amount of the uptaken cpt esters, rather than only the proportion of the prodrugs activated in cells (Table S8). Therefore, the data should correlate with those obtained based on the quantification of intracellular boronic acid (described in the next section).

Table **S8**. Efficiency of uptake of prodrugs, reference and control compounds by BL-2 cells (monitored by detecting intracellular fluorescence of HO-cpt).

Prodrug / control	Fluorescence (a.u.= arbitrary units)
HO-cpt	62 ± 2
**2**	111 ± 12
**3**	148 ± 27
Carrier (negative control)	3 ± 2

<u>Assessing the uptake of the prodrug by BL-2 cells via monitoring the intracellular concentration of boronic acid</u>

BL-2 cell suspension containing 10^6 cells/mL, prepared as described above in the section "**Monitoring the uptake of the prodrug by BL-cells by quantification of intracellular fluorescence signal characteristic for camptothecin derivatives**", was spread in the wells of a 6 well microtiter plates (12 wells for each compound, volume of a well was 3 mL and total volume for every drug/prodrug was 36 mL). Solutions of the prodrug (30 µL per well, solvent DMSO) were added to the suspensions and incubated for 1 h. The final concentration of the prodrug in the suspensions was 1 µM. Incubation of BL-2 cells with these concentrations drugs/prodrugs did not result in morphological changes of the cells.

The suspensions from 12 wells treated at the same condition were combined, the combined suspensions were washed three times with ice-cold PBS (3 × 5 mL), treated with concentrated H_2O_2 solution (200 μL, 1 M, the final concentration 6 mM) for 30 min, and all volatiles were removed by lyophilization. Dry, lysed cells were washed with water (200 μL), and aqueous solution obtained was acidified with HCl (400 μL, 0.1 M). Then this solution was extracted with 2-ethyl-1,3-hexanediol (160 μL, 10% in $CHCl_3$, v/v), and a portion of the organic phase obtained (90 μL) was mixed with H_2SO_4/CH_3CO_2H (400 μL, 1/1, v/v). Curcumin solution in methyl isobutyl ketone (250 μL, 2 mg in 1 mL of the solvent) was added and allowed to react for 24 h. The reaction was quenched by addition of water (1 mL). The light absorbance at 550 and 780 nm of the organic phase was measured. The former value corresponds to absorbance of curcumin−boron complex, whereas the latter one is taken as a baseline. The baseline corrected absorbance at 550 nm (A(550 nm) − A(780 nm)) was proportional to the concentration of boron in the mixture (Table S9).

Table **S9**. Efficiency of uptake of prodrugs, reference and control compounds by BL-2 cells (monitored by detecting intracellular boronic acid).

Prodrug / control	ΔA= A(550 nm) – A(780 nm) Absolute values / Relative uptake[i]
**2**	1.23 ± 0.03 / 56 %
**5**	0.98 ± 0.03 / 41 %
Carrier (negative control)	0.35 ± 0.01 / 0 %

[i] Relative uptake of a prodrug, $RU_{prodrug}$= 100 % * ($\Delta A_{prodrug}$ - $\Delta A_{negative\ control}$)/($\Delta A_{reference}$ - $\Delta A_{negative\ control}$).

Release of ROS in FaDu cells treated with IR

The day before the experiment, FaDu cells were seeded in 48-well plates at a concentration of 250 cells/µL (200 µL/well). Before the experiment, the cells were irradiated with 1x10 Gy ionizing radiation (IR) by an ISOVOLT Titan X-ray generator (GE, Ahrensburg, Germany). Subsequently, the cells were either immediately (0 Min post-RT) washed twice and MitoSOX™ Red ROS indicator in DPBS was added for 20 min: final concentration – 2.5 µM, or incubated for 30 minutes post-RT and washed and stained with MitoSOX according to the previous description. Finally, the cells were washed twice with DPBS, detached from the microtiter plate by using trypsin/EDTA (0.25% trypsin, v/v, 100 µL), and resuspended in the culture medium (200 µL). The cells were transferred to a 96-well-plate and centrifuged at 400 x g for 6 minutes. Afterwards, supernatant was discarded and cells were resuspended in 50 µL of DPBS (2 % FCS). The fluorescence of all viable cells was determined using flow cytometry (Excitation at 488 nm, Emission at 610/20 nm) with a CytoFLEX S Flow Cytometer (N = 3) (Figure S33).

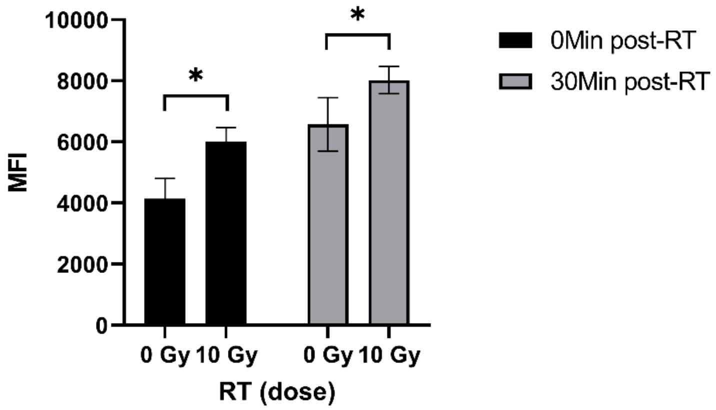

* Each value represents mean ± SD (n = 3).
Significance was determined by one-tailed Mann Whitney U test * $p \leq 0.05$.

MFI = mean fluorescence intensity

Figure **S33**. Effects of ionizing radiation (IR) in intracellular ROS level in FaDu cells.

Radiosensitizing effect of the prodrug and the control

Colony formation assays were performed for monitoring radioresponse as previously described.[S41] Here, increasing numbers of single cells relative to the irradiation dose (0-

10 Gy) were seeded into 6-well plates (150-2400 SAS cells/well and 300-4800 FaDu cells/well). Cells were treated with the drugs upon attachment for a total of 48 h as depicted in a previous section and single dose irradiation was carried out 24 h after addition of the compounds. X-ray was performed at room temperature with a YxlonY.TU 320 system (Yxlon.international, 200 kV, 0.5 mm Cu filter) and a dose rate of ~1.3 Gy/min. Drug exposure was terminated by medium renewal, and cultures were then incubated for an additional period of 7 days (SAS) and 10 days (FaDu). Plating efficiencies were assessed and normalized for each treatment arm to the respective non-irradiated control. Data were reproduced (N=3 each with n=3 wells per experiment and treatment condition). The relative clonogenic cell survival (= surviving fraction, SF) was then plotted as function of the irradiation dose, and clonogenic survival curves were fitted employing the linear quadratic model as described elsewhere.[S42] The results documented in Figure S34 indicate a radiosensitizing potential of the prodrug **2** but not of the control substance **3**.

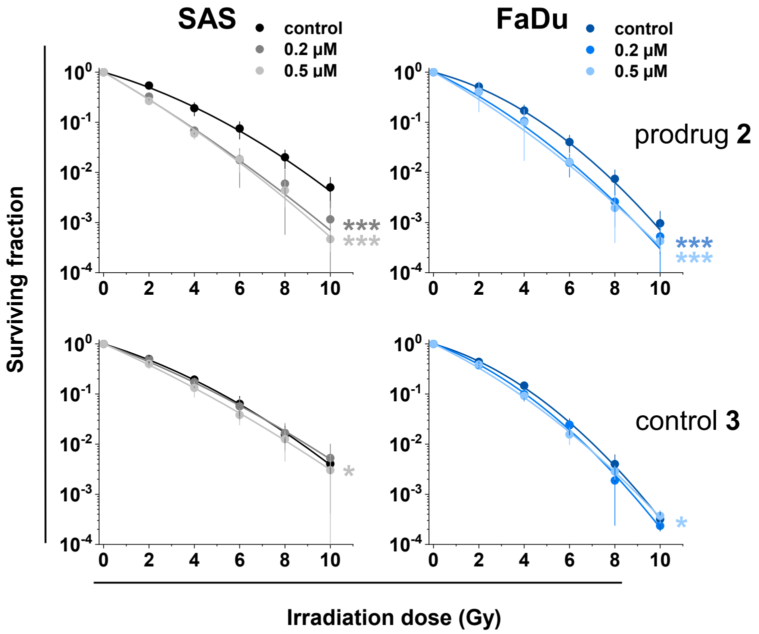

Figure **S34**. Data illustrating the synergy of the prodrug **2** with radiotherapy in SAS and FaDu cells. The graphs show clonogenic survival as a function of the irradiation dose. The data points represent mean values (±SD) from N≥3 independent experiments and the lines display the clonogenic survival curve fits. * $p<0.05$; *** $p<0.001$.

Reaction of the prodrug **2** with H_2O_2 in cells

Fluorescence microscopy

Probing localization of the fluorescent intermediates derived from the prodrug in mitochondria:

A2780 cells (80 cells/μL, in 500 μL culture medium containing 5% FBS) were seeded in imaging dishes and incubated overnight. The next day, the cells were washed and pre-incubated incubated with the known mitochondria labeling dye rhodamine 123 (R123, 1 μM, in Hank's buffered salt solution, HBSS, 2 mL/dish) and incubated for 20 min. The samples were washed twice with PBS (2 mL) and the prodrug **2** (20 μM) in culture medium (RPMI 1640, 5% FBS, 1% DMSO, 2 mL/dish) was added. After 2 h incubation, the samples were again washed twice with PBS and a fresh portion of HBSS was added before imaging with a Zeiss AX10 Lab A1. ***P**rodrug-**d**erived **f**luorescent **p**roducts* (PDFPs) were detected at λ_{ex} = 365 nm, λ_{em} = 420–470 nm (channel 1: Ch1). R123 was detected at λ_{ex} = 430–510 nm, λ_{em} = 475-575 nm (channel 2: Ch2) (Figure 6A, main part of the paper).

In a control experiment, A2780 cells were incubated either only with the prodrug **2** (20 μM) or only with R123 (1 μM) and images were acquired by detecting signals in Ch1 and Ch2. The obtained data confirmed that the selected detection conditions are specific for PDFPs and R123 dye (Figure S35).

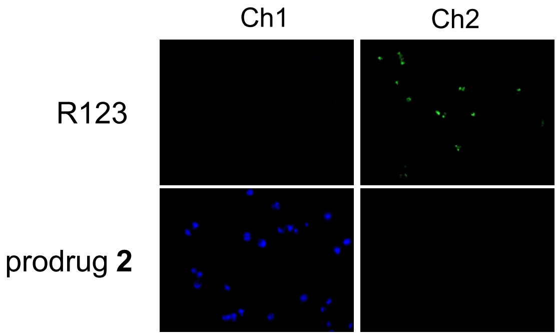

Figure **S35**. Fluorescence microscopic images of A2780 cells incubated either with the prodrug **2** (20 μM) or mitochondria-labelling dye R123 (1 μM). Two channels were used for emission monitoring: Ch1 - λ_{ex} = 365 nm, λ_{em} = 420–470 nm and Ch2 - λ_{ex} = 430–510 nm, λ_{em} = 475-575 nm.

Probing localization of the fluorescent intermediates derived from the prodrug in nuclei: A2780 cells (80 cells/μL, in 500 μL culture medium containing 5% FBS) or SBLF9 cells (40 cells/μL, in 500 μL culture medium containing 15% FBS) were seeded in imaging dishes and incubated overnight. The next day, the cells were washed and incubated with the prodrug **2** (20 μM) in culture medium (RPMI 1640, 5% FBS, 1% DMSO, 2 mL/dish). After 2 h incubation, the samples were again washed twice with PBS and a fresh portion of HBSS containing NUCLEAR-ID® Red dye (1:2000 dilution, 2 mL) or just HBSS was added and incubated for 20 min at 37 °C. The samples were washed again twice and a fresh portion of HBSS (1 mL) was added before imaging with a Zeiss AX10 Lab A1. PDFPs were detected using channel 1 (Ch1) at λ_{ex} = 365 nm, λ_{em} = 420–470 nm. NUCLEAR-ID® Red dye (NIRD) was detected using channel 3 (Ch3) at λ_{ex} = 520–570 nm, λ_{em} = 535–675 nm. (Figure 6A, main part of the paper).
In a control experiment, A2780 cells were incubated either only with the prodrug **2** (20 μM) or only with NIRD and images were acquired by detecting signals in Ch1 and Ch3. The obtained data confirmed that the selected detection conditions are specific for PDFPs and NIRD (Figure S36).

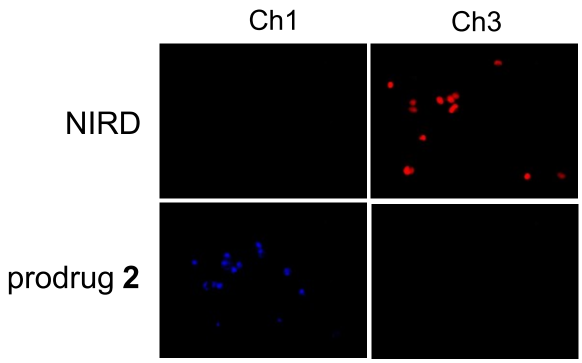

Figure **S36.** Fluorescence microscopic images of A2780 cells incubated either with the prodrug **2** (20 μM) or nuclei-labelling dye NIRD. Two channels were used for emission monitoring: Ch1 - λ_{ex} = 365 nm, λ_{em} = 420–470 nm, Ch3 - λ_{ex} = 520–570 nm, λ_{em} = 535–675 nm.

To investigate the effects of ROS scavenging compounds on the activation and the subcellular distribution of prodrug **2**, the same experiment was repeated but with pre-

treatment of the cells with N-acetyl-cysteine (NAC, 2 mM, 1h) before **2** was added. Imaging was performed as described above (main text, Figure 6A, compare middle and right images).

<u>Exploring the possibility that the prodrug induces the release of reactive oxygen species (ROS) in cells free settings and in cells</u>

Release of ROS in cell free settings

2′,7′-Dichlorofluorescin diacetate (DCFH-DA; 4.9 mg) was dissolved in N,N-dimethylformamide (DMF, 100 µL) and mixed with aqueous NaOH (0.1 M, 900 µL). The resulting mixture was incubated for 30 min at 22 °C in the dark. This solution was diluted to obtain a solution containing 2′,7′-dichlorofluorescin (DCFH, 10 µM), 3-(N-morpholino)propanesulfoic acid (MOPS) buffer (100 mM, pH 7.5), N,N,N',N'-ethylenediamine tetracetic acid (EDTA, 10 mM), glutathione (GSH, 5 mM), and H_2O_2 (10 mM). Monitoring of the fluorescence (λ_{ex}= 501 nm, λ_{em}= 531 nm) of this solution was started. After 5-10 minutes of the incubation prodrug **2** (2 µM) and control **5** (32 µM) were added at final concentrations equal to their IC_{50} values (BL-2 cells, 48 h incubation, Table S5) and measurement was continued for further 235 min (Figure S37).

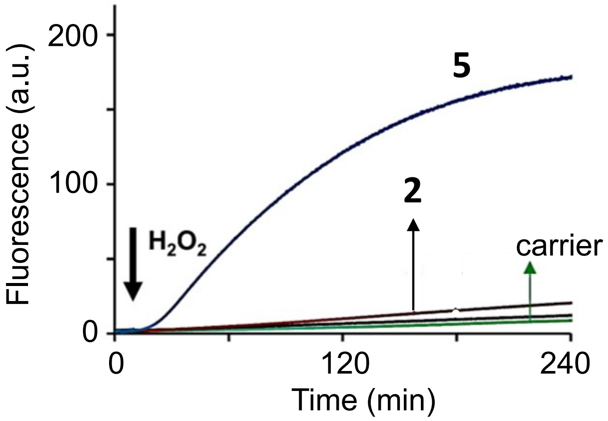

Figure **S37**. Monitoring oxidation of ROS probe 2′,7′-dichlorofluorescin in the presence of the prodrug **2** and the control **5** as well as H_2O_2 as a bulk oxidation agent. The time point of H_2O_2 addition is indicated with a black solid arrow. “Carrier” is DMSO, 1%, v/v. Other conditions are described in the experimental procedure above.

Release of ROS in BL-2 cells

BL-2 cells were grown as mentioned before, centrifuged (5 min; 1000 rpm), washed with DPBS (2×10 mL) and the suspension at the cell density of 10^6 cells/mL in DPBS was prepared. 5(6-)chloromethyl-2′,7′-dichlorodihydrofluoresceindiacetate (CM-DCFH-DA) in DMSO (8.7 µL, stock solution: 10 mM) was added into the cell suspension (8.7 mL) and incubated in the dark chamber filled with CO_2 (5%) at 37 °C for 15 min. Afterwards the cells were washed with DPBS (10 mL), re-suspended in Opti-MEM medium (8.7 mL) and split to portions of 200 µL. The prodrug **2** (2 µM) and the control **5** (32 µM) were added at final concentrations equal to their IC_{50} values (BL-2 cells, 48 h incubation, Table S5). After 5 hours of incubation in the dark chamber filled with CO_2 (5%) at 37 °C, the mean fluorescence of live cells (λ_{ex} = 488 nm, λ_{em} = 530 nm) in the suspensions was determined by using the flow cytometry (Figure S38).

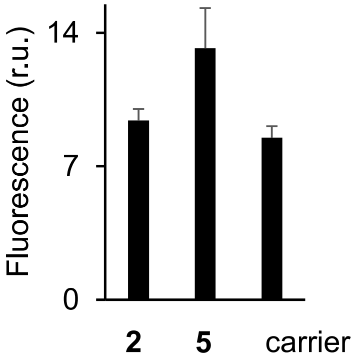

Figure **S38**. Monitoring generation of ROS in BL-2 cells in the presence of the prodrug **2** and the control **5**. “Carrier” is DMSO, 1%, v/v. The fluorescence (λ_{ex} = 488 nm, λ_{em} = 530 nm) correlates with the level of intracellular ROS. Other conditions are described in the experimental procedure above.

Release of ROS in A2780 cells

The day before the experiment, A2780 cells were seeded in 96-well plates at a concentration of 250 cells/µL (100 µL/well). Before the experiment, the cells were washed twice with PBS (100 µL) and incubated with the prodrug **2** (7 µM), reference 4-(N-

ferrocenyl-N-(3-piperdinylmethylphenylmethyl)carbonyloxymethyl)-fluorobenzol[S43] (10 μM) or carrier only (DMSO, 1 %, v/v) for 24 h. Subsequently, the cells were washed twice and MitoSOX™ Red ROS indicator in HBSS was added for 20 min: final concentration - 5 µM. Finally, the cells were washed twice with PBS, detached from the microtiter plate by using trypsin/EDTA (0.25% trypsin, v/v, 50 µL), and resuspended in the culture medium (100 µL). The fluorescence of all viable cells from a total cell count of 5000 was determined using flow cytometry (λ_{ex} = 480-496 nm, λ_{em} = 543-627 nm) with a CytoFLEX S Flow Cytometer or the Gallios cytofluorometer™ flow cytometer (N = 3) (Figure S39).

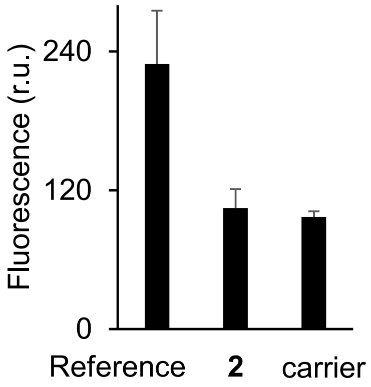

Figure **S39**. Release of ROS in A2780 cells in the presence of the prodrug **2** and the known reference compound ("Reference", 4-(N-ferrocenyl-N-(3-piperdinylmethylphenylmethyl)carbonyloxymethyl)fluorobenzol). Other conditions and experimental details are described above.

Effect of the prodrug on cell cycle of A2780 cells

A2780 cells were seeded in a 24-well microtiter plate at a density of 200 cells/µL in solution containing FBS (20%), GlutaMAX™ (1%), penicillin/streptomycin (1%) (total volume 500 µL). Solutions of the prodrug $FcN^{PG2/PG1}$-L-E-cpt (0.5 μM), drug HO-cpt (5 nM) (both dissolved in DMSO, 2.5 µL) and DMSO carrier (1 %, v/v, labelled "-") were added. After 72 h incubation, the cells were transferred to 1.5 mL Eppendorf tubes and centrifuged at 180 rcf for 5 min. The supernatants were discharged, and the cell pellets were washed with PBS (500 µL). Then propidium iodide (PI) staining solution (200 µL)

was added to each Eppendorf tube. The PI staining solution (10 mL) contains 1 mg RNAse, 0.5 mg of PI, 0.1mL of Triton X-100 and 0.1 mg sodium citrate. The cells were resuspended and incubated in the dark for 2 h at 4 °C. Then the cells were pipetted up and down to ensure homogeneity and minimize cell agglomeration before investigating them with a CytoFLEX S Flow Cytometer or the Gallios cytofluorometer™ flow cytometer at channel PE (λ_{ex}= 480-496 nm, λ_{em}= 543-627 nm). The data was processed using ModFit LT™ 5.0 software from Verity Software House (Figures 6B, C, main text of the paper).

Experiments with neutrophils

General

For each experimental day 30 mL blood were drawn from a healthy donor with lithium heparin as an anticoagulant and diluted with 10mL PBS (10 mM, 150 mM NaCl, pH 7.4). In two centrifugation tubes, 15 mL lymphophlot was added, overlayed with 20 mL of the blood and centrifuged (30 min, 18 °C, 350 rcf, slow acceleration, no break). The polymorphonuclear leukocytes (PMNs) were carefully isolated from the surface of the erythrocyte pellet. To lyse the erythrocytes, the cells were mixed with millipore water (36mL) for 20 s, before 10x PBS (100 mM, 1.5 M NaCl, pH 7.2, 4 mL) was added. The cells were collected by centrifugation (7 min, 20 °C, 180 rcf) and the supernatant discarded. The erythrolysis was repeated with 45 mL millipore water and 5 mL 10x PBS. The cell concentration was adjusted to 500 cell/µL in RPMI 1640 (5% FBS, 1% Pen/Strep, 1% GlutaMax).

Study of distribution of PDFPs

For fluorescence microscopy, freshly isolated neutrophiles (500 cells/µL) were incubated with **2** (20 µM) for 2 h, subsequently they were washed with PBS (centrifugation at 180 rcf, 7 min) and a fresh portion of HBSS containing NUCLEAR-ID® Red dye (1:2000 dilution, 2 mL) was added. Same operations were conducted with the cells pre-incubated

for 15 min with PMA (50 nM) in RPMI 1640 cultivation medium. The cells were washed once again before imaging with a Axio Observer Z1 with a Plan-Apochromat 63x/1.4 Oil Ph 3 M27 objective, channel 1 (Ch1) at λ_{ex} = 335-383 nm, λ_{em} = 420–470 nm. NUCLEAR-ID® Red dye (NIRD) was detected using channel 3 (Ch3) at λ_{ex} = 538–562 nm, λ_{em} = 570–640 nm. The data obtained are shown in Figure 6D, main text of the paper.

Study of cytotoxicity of the prodrug and drug HO-cpt in the presence of PMA

For the toxicity assay, freshly isolated neutrophiles (500 cells/µL) were pre-incubated with PMA (50 nM) for 15 min in RPMI 1640 cultivation medium. Subsequently, either **2** or HO-cpt (20 µM) was added and incubated for 2 h. Finally, the cells were mixed 1:1 with 0.1% (m/v) trypan blue staining solution and 10 µL of each sample was transferred to a Neubauer containing chamber in which viable (unstained) and dead cells (dark stained) cells were counted (4x4x4 fields per sample). The data obtained are shown in Figure 6D, main text of the paper.

Assessment of activation of **2** in blood and bone marrow in wild-type mice by monitoring PDFPs

Mice were treated with either DMSO as a vehicle or prodrug **2** (12 mg/kg, the dose optimized in the previous experiment) intraperitoneally (i.p.). The mice were analysed 2, 5, 24 and 48h later.

Preparation of single-cell suspensions from tissues

Bone marrow was isolated from the femur by centrifugation in a 0.5 mL Eppendorf tube for 30 seconds at maximum speed (13.200rpm). The cell pellet was lysed for red blood cells (RBC) in a hypotonic buffer for 5 minutes at 22 °C and washed with 2 mM EDTA, 2% FBS in PBS buffer.

Blood was extracted from the mouse cheek and collected in EDTA-coated tubes (Starstedt). Blood red blood cells (RBC's) were lysed in the hypotonic buffer for 10 minutes at 22 °C and washed with 2 mM EDTA, 2% FBS in PBS buffer. Peripheral blood counts were analysed in an automated haemocytometer (Pentra, Horiba) according to the manufacturer's instructions.

Flow Cytometry

Cytometric analyses were performed using a Canto 3L HTS flow cytometer (BD) equipped with DIVA software and data were analysed with FlowJo (FlowJo LLC, Ashland, OR) software.

For the analysis of LSK and MPs, cells were stained with biotinylated lineage antibody cocktail (CD3e, B220, CD11b, Gr1 and Ter119 (1:100), together with streptavidin conjugated to PE, FITC-conjugated Sca-1 (1:200) and PE-Cy7-conjugated c-Kit (1:200) for 30 min at 4ºC.

- LSK stands for Lineage^{NEG} Sca-1^{+} c-Kit^{+} and contains the most primitive fraction of hematopoietic stem cells.
- MP stands for myeloid progenitors

For assessment of the mean fluorescence intensity (MFI) in neutrophils, cells were stained with APC-conjugated Ly6G (1:200), BV510-conjugated CD11b (1:200), PerCP-Cy5.5-conjugated CXCR2 antibody (1:200), PE-conjugated CXCR4 (1:200) and FITC-conjugated CD62L (1:200). Cells were gated on Ly6G, CD11b-positive cells for neutrophils. Intracellular fluorescence of intermediated released from prodrug FcN$^{PG2/PG1}$-L-E-cpt was assessed using the DAPI channel.

Quantification of HSPC numbers in bone marrow and peripheral blood

To measure absolute numbers of cells present in the tissues, TruCount beads (BD) were prepared at a concentration of 10,000 beads per ml of PBS buffer. 300 μL of the bead suspension was added to the single cell suspensions stained for flow cytometry and the cell number values were calculated based on the number of beads per tube and corrected by the volume of tissue analysed.

Statistical analysis

Data from experiments are shown as the mean values ± standard error of the mean (SEM). For data with more than two data sets, one-way or two-way analysis of variance (ANOVA) with Bonferroni multi-group test was used. All statistical analyses were performed with GraphPad Prism software v8 (GraphPad software, California, USA). P values < 0.05 were considered statistically significant and non-significant differences (ns) are indicated accordingly.

Evaluation of effects of prodrug **2** and control HO-cpt on bone marrow and blood cells

Mice were injected with either prodrug **2**, drug HO-cpt (positive control) or the carrier only (DMSO, negative control) at day 0, 2 and 4 and analysed at day 7. Mice were weight every day except on day 3. The samples were injected i.p. at the following doses: prodrug **2** (12 mg/kg), drug HO-cpt (6 mg/kg). None of the conditions affected the weight and overall physical appearance of the mice except for drug HO-cpt (positive control) (Figures 6E, F, main text of the paper).

Preparation of single-cell suspensions from tissues, flow cytometry measurements, quantification of HSPC numbers in BM and peripheral blood and statistical analysis

These studies were conducted as described in the previous section. The data are provided in Figures S40A-C.

A:

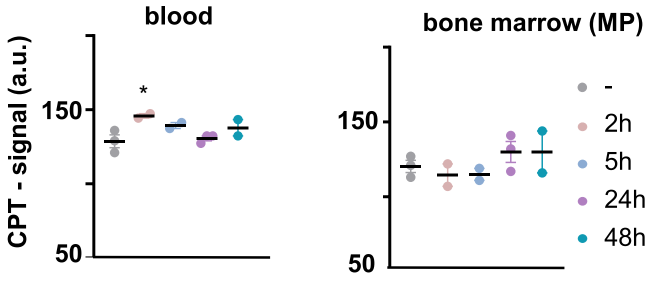

B:

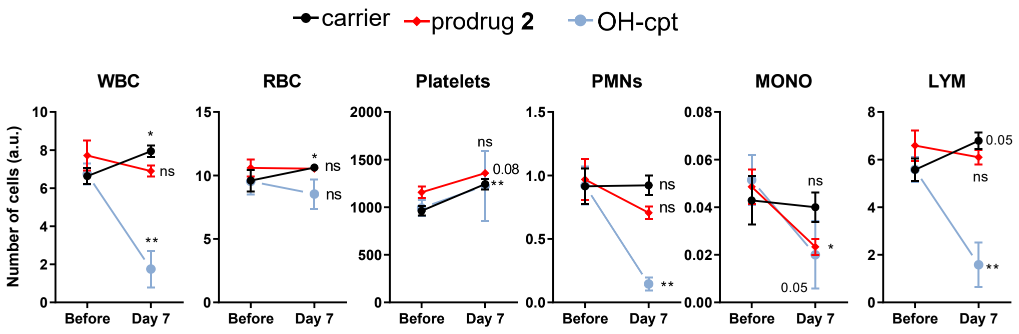

C:

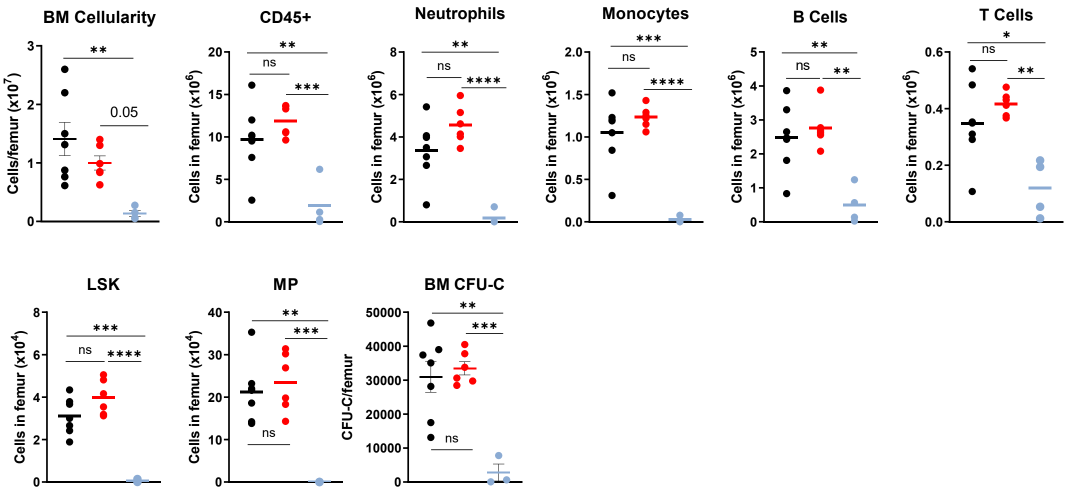

Figure **S40**. **A**: Monitoring the fluorescence signal characteristic for PDFPs (DAPI channel) in neutrophils from blood (left) and bone marrow (BM, right). **B**, **C**: Monitoring of the number of cells in blood (**B**) and bone marrow (**C**) of mice treated either with DMSO only (carrier: "-") or prodrug **2** (12 mg/kg) or drug HO-cpt (6 mg/kg). These reagents were injected i.p. on days 0, 2 and 4. The analysis was conducted on day 7. Paired t-test was conducted between days 0 and 7 (**B**). The data in **C** were analyzed by one-way ANOVA with multiple comparisons. Abbreviations – WBC: white blood cells; RBC: red blood cells; PMNs: polymorphonuclear neutrophils; MONO: monocytes; LYM: lymphocytes; BM: bone marrow; LSK: Lineage^{NEG} Sca-1^{+} c-Kit^{+}; MP: myeloid progenitors.

In vivo effects of the prodrug 2 on organs, tissues and cells in healthy animals

Mice

Black C57/BL6N mice and white mice Balb/c were bred at Animal house of Danylo Halytsky Lviv National Medical University (Lviv, Ukraine). The animal studies were approved by the local ethical committee (Permission to R. Bilyy; 20180226/P2 and 2014-2018/P6) and conducted according to the guidelines of the Federation of European Laboratory Animal Science Associations (FELASA). Animals were kept in clean environment, with water and food available ad libitum, under conditions of controlled temperature, humidity, luminosity and under professional supervision of veterinary doctors.

For tumor growth experiments mice weighting 30-35 g were used. For other experiments 12 week-old mice weighting 20-25 g were used.

Toxicity of the prodrug

The goal of this experiment was to estimate toxicity of the prodrug and to find the optimal dose for further in vivo experiments. We used 12 mice (Balb/c) grouped in 4 groups, each consisting of 3 animals. The animals were injected (*i.p.*, injection volume: 30 μL, the prodrug was dissolved in DMSO) to achieve the following final doses: 0, 3, 6, 12 and 24 mg/kg. The injections were made at days 0, 2 and 4. The animals were weighed before the experiment (day -1) and on days 2, 4 and 7. No changes were detected in animal behavior at day 7 of the experiment. All animals appeared active and healthy (see supplementary video file Exp1_Day7.3gp where littermates from different cages were merged for behavior testing). Furthermore, no statistically significant changes in weight of the animals were observed upon their treatment with the prodrug at doses between 3 and 24 mg/kg (Figure S41, Table S10).

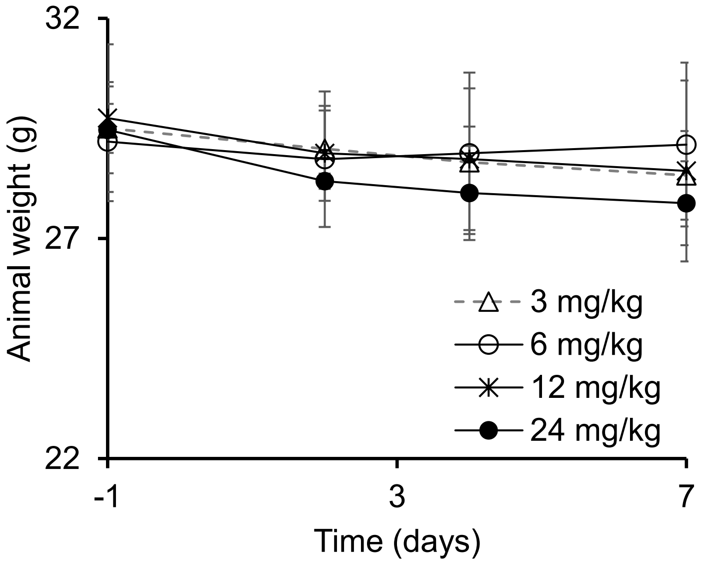

Figure **S41**. Change of weight of Balb/c mice caused by their treatment with the prodrug at the indicated doses. Other experimental conditions are described above.

Table **S10**. Comparison of weight of Balb/c mice before treatment (day -1) and 7 days after the treatment with the prodrug at different doses by using Student's t test (p-values).[a]

Dose, mg/kg	p-value
3	0.1835
6	0.9435
12	0.4774
24	0.1027

[a] Experimental values, for which $p < 0.05$, are considered different.

Selection of the optimal dose of the prodrug

No visible damages to internal organs were observed at all tested conditions. However, at the highest used prodrug dose of 24 mg/kg we observed substantial accumulations of intensely yellow colored compounds (resembling the appearance of the solid prodrug **2**)

on the surface of internal organs of peritoneal cavity and near the places of intraperitoneal injections (Figure S42).

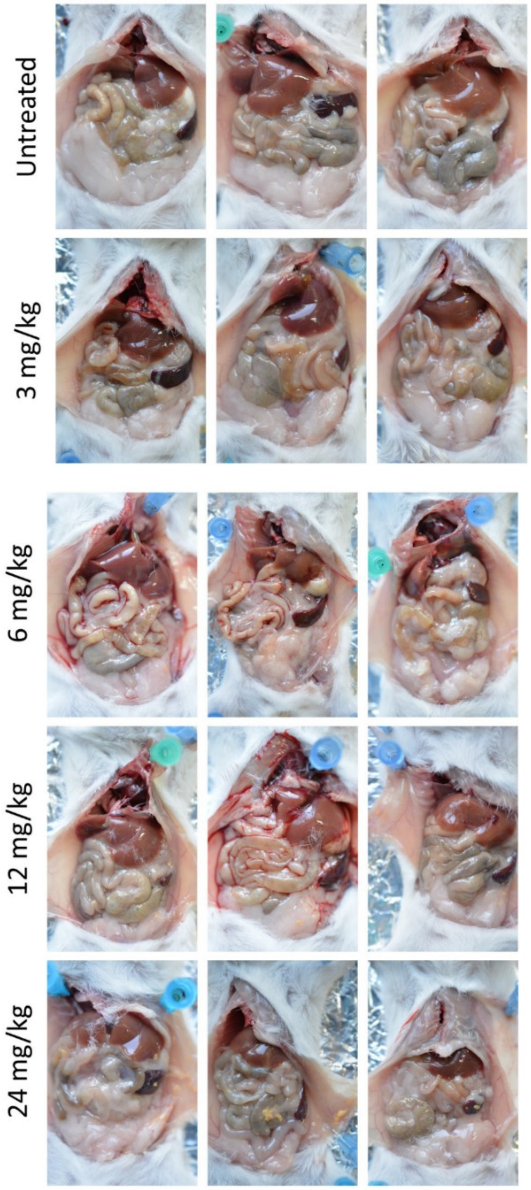

Figure **S42**. Photos of inner organs of animals treated with the prodrug. Other experimental details are provided above.

We assume that the prodrug precipitates upon the contact with water-based medium (tissue fluids). Based on these data we selected the dose of 12 mg/kg for further experiments.

Accumulation of the prodrug in organs

At day 7 the animals were sacrificed, dissections were made and organs were removed. Organs of three control mice (untreated) and three mice, which received with highest dose of 24 mg/kg, were analyzed. The following organs were obtained: blood; liver; spleen; kidneys; lung; heart; adipose tissue; muscle; peritoneum. The organs were weighed and flash-frozen in liquid nitrogen. Then they were homogenized in hypotonic buffer (10mM Tris-HCl, pH 7,5; 5 mM EDTA), ultra-sonicated and centrifuged. The obtained supernatants were split in two parts. The first part was treated with H_2O_2 (100 mM) for 2h at 37°C. The second part was treated with H_2O for 2h at 4°C. Then at least 1/10 of volume of butyl acetate was added for extraction to both parts. The samples were mixed and the organic phase was collected and transferred to multiwall plates. The fluorescence of solutions was measured (λ_{ex} = 365 nm, λ_{em} = 450 nm) using Perkin Elmer BioAssay reader HST7000. The prodrug (60 µM) pre-treated with H_2O_2 (100 mM) for 2h at 37°C was used as a positive control. Results of this analysis are presented in Figures S43 and S44.

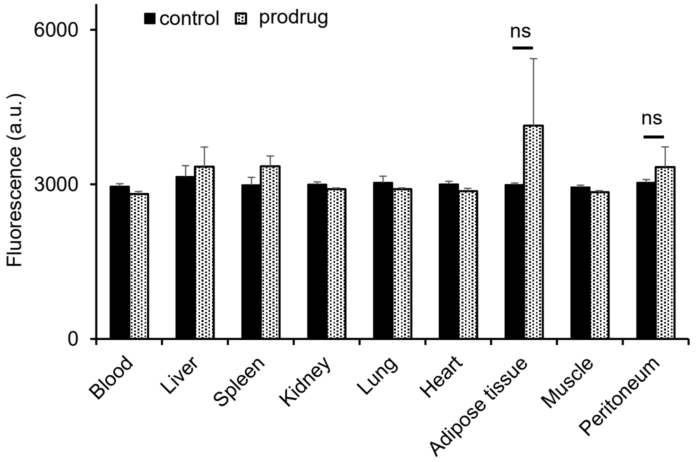

Figure **S43**. Fluorescence of butyl acetate extracts from organs of untreated and treated with the prodrug **2** (24 mg/kg) mice: λ_{ex} = 365 nm, λ_{em} = 450 nm. No statistically significant differences between the fluorescence signals obtained in the treated and untreated groups were observed by using Student's t-test. The label "ns" means $p \geq 0.05$.

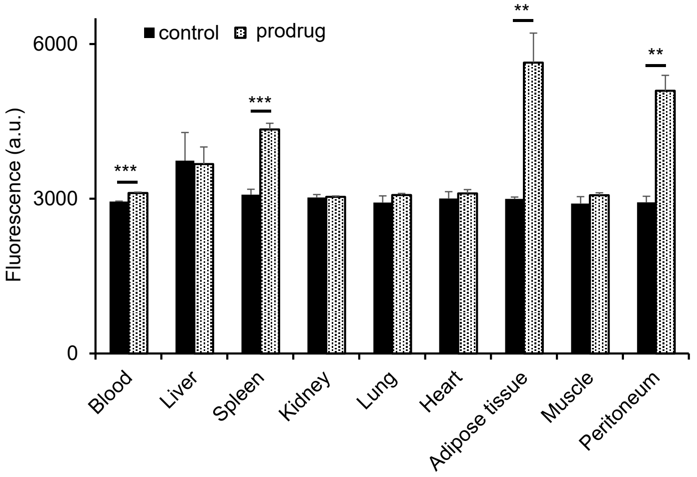

Figure **S44**. Fluorescence of butyl acetate extracts from organs of untreated and treated with the prodrug **2** (24 mg/kg) mice: λ_{ex} = 365 nm, λ_{em} = 450 nm. Before the extraction the supernatants obtained from the organs were treated with the large excess of H_2O_2 (100 mM) to activate the prodrug. Student's t test: ** - $p< 0.01$; *** - $p< 0.001$. Data pairs in control and treated groups, which are not labelled with either ** or *** are not significantly different from each other.

Evaluation of the effect of the prodrug on the survival of mice after inoculation of Nemeth-Kellner (NK) lymphoma (Ly) NK/Ly

Tumors were transplanted by intraperitoneal inoculation (growth as lymphoma) of aliquots of ascites containing $7{,}5*10^5$ NK/Ly cells into C57/BL6N mice (day 0). Two groups of animals each consisting of 14 animals were injected with the prodrug **2** (dose of 12 mg/kg, 30 μL) or DMSO carrier (30 μL). 10 treatments were made at days 1, 3, 6, 8, 10, 13, 15, 17, 20 and 22. Animal survival was monitored till day 87 (Figure 7B, main text of the paper).

References

S1. V. Reshetnikov, S. Daum, C. Janko, W. Karawacka, R. Tietze, C. Alexiou, S. Paryzhak, T. Dumych, R. Bilyy, P. Tripal, B. Schmid, R. Palmisano, A. Mokhir, *Angew. Chem. Int. Ed.* **2018**, *57*, 11943-11946.

S2. S. Daum, S. Babiy, H. Konovalova, W. Hofer, A. Shtemenko, N. Shtemenko, C. Janko, C. Alexiou, A. Mokhir, *J. Inorg. Biochem.* **2017**, *178*, 9-17.

S3. A. Arlegui, P. Torres, V. Cuesta, J. Crusats, A. Moyano, *Eur. J. Org. Chem*. **2020**, *28*, 4399-4407.

S4. Pathak, R.K., McNitt, C.D., Popik, V.V. and Dhar, S., *Chem. Eur. J.*, **2014**, *20*, 6861-6865.

S5. B. Selvi, S. Patel, M. Savva, *J. Pharm. Sci*. **2008**, *97*, 4379-4390.

S6. Case, D. A.; Belfon, K.; Ben-Shalom, I. Y.; Brozell, S. R.; Cerutti, D. S.; Cheatham III, T. E.; V.W.D. Cruzeiro; Darden, T. A.; Duke, R. E.; Giambasu, G.; Gilson, M. K.; Gohlke, H.; Goetz, A. W.; Harris, R.; Izadi, S.; Kasavajhala, K.; Kovalenko, A.; Krasny, R.; Kurtzman, T.; Lee, T. S.; LeGrand, S.; Li, P.; Lin, C.; Liu, J.; Luchko, T.; Luo, R.; Man, V.; Merz, K. M.; Miao, Y.; Mikhailovskii, O.; Monard, G.; Nguyen, H.; Onufriev, A.; Pan, F.; Pantano, S.; Qi, R.; Roe, D. R.; Roitberg, A.; Sagui, C.; Schott-Verdugo, S.; Shen, J.; Simmerling, C. L.; Skrynnikov, N.; Smith, J.; Swails, J.; Walker, R. C.; Wang, J.; Wilson, L.; Wolf, R. M.; Wu, X.; York, D. M.; Kollman, P. A. AMBER 2020, University of California: San Francisco, **2020**.

S7. Wang, J.; Wolf, R. M.; Caldwell, J. W.; Kollman, P. A.; Case, D. A. *J. Comput. Chem.* **2004**, *25*, 1157-1174.

S8. Wang, J.; Wang, W.; Kollman, P. A.; Case, D. A. *J. Mol. Graphics Modell.* **2006**, *25*, 247-260.

S9. Cieplak, P.; Cornell, W. D.; Bayly, C.; Kollman, P. A. *J. Comput. Chem.* **1995**, *16*, 1357-1377.

S10. Bayly, C. I.; Cieplak, P.; Cornell, W.; Kollman, P. A. *J. Phys. Chem.* **1993**, *97*, 10269-10280.

S11. Frisch, M. J.; Trucks, G. W.; Schlegel, H. B.; Scuseria, G. E.; Robb, M. A.; Cheeseman, J. R.; Scalmani, G.; Barone, V.; Petersson, G. A.; Nakatsuji, H.; Li, X.; Caricato, M.; Marenich, A. V.; Bloino, J.; Janesko, B. G.; Gomperts, R.; Mennucci, B.; Hratchian, H. P.; Ortiz, J. V.; Izmaylov, A. F.; Sonnenberg, J. L.; Williams; Ding, F.; Lipparini, F.; Egidi, F.; Goings, J.; Peng, B.; Petrone, A.; Henderson, T.; Ranasinghe, D.; Zakrzewski, V. G.; Gao, J.; Rega, N.; Zheng, G.; Liang, W.; Hada, M.; Ehara, M.; Toyota, K.; Fukuda, R.; Hasegawa, J.; Ishida, M.; Nakajima, T.; Honda, Y.; Kitao, O.; Nakai, H.; Vreven, T.; Throssell, K.; Montgomery Jr., J. A.; Peralta, J. E.; Ogliaro, F.; Bearpark, M. J.; Heyd, J. J.; Brothers, E. N.; Kudin, K. N.; Staroverov, V. N.; Keith, T.

A.; Kobayashi, R.; Normand, J.; Raghavachari, K.; Rendell, A. P.; Burant, J. C.; Iyengar, S. S.; Tomasi, J.; Cossi, M.; Millam, J. M.; Klene, M.; Adamo, C.; Cammi, R.; Ochterski, J. W.; Martin, R. L.; Morokuma, K.; Farkas, O.; Foresman, J. B.; Fox, D. J. Gaussian 16 Rev. B.01, Wallingford, CT, **2016**.

S12. Ditchfield, R.; Hehre, W. J.; Pople, J. A. *J. Chem. Phys.* **1971**, *54*, 724-728.

S13. Hehre, W. J.; Ditchfield, R.; Pople, J. A. *J. Chem. Phys.* **1972**, *56*, 2257-2261.

S14. Hariharan, P. C.; Pople, J. A. *Theor. Chem. Acc.* **1973**, *28*, 213-222.

S15. Hariharan, P. C.; Pople, J. A. *Mol. Phys.* **1974**, *27*, 209-214.

S16. Gordon, M. S. Chem. *Phys. Lett.* **1980**, *76*, 163-168.

S17. Francl, M. M.; Pietro, W. J.; Hehre, W. J.; Binkley, J. S.; DeFrees, D. J.; Pople, J. A.; Gordon, M. S. *J. Chem. Phys.* **1982**, *77*, 3654-3665.

S18. Binning Jr., R. C.; Curtiss, L. A. *J. Comput. Chem.* **1990**, *11*, 1206-1216.

S19. Blaudeau, J.-P.; McGrath, M. P.; Curtiss, L. A.; Radom, L. *J. Chem. Phys.* **1997**, *107*, 5016-5021.

S20. Rassolov, V. A.; Pople, J. A.; Ratner, M. A.; Windus, T. L. *J. Chem. Phys.* **1998**, *109*, 1223-1229.

S21. Rassolov, V. A.; Ratner, M. A.; Pople, J. A.; Redfern, P. C.; Curtiss, L. A. *J. Comput. Chem.* **2001**, *22*, 976-984.

S22. Becke, A. D. *J. Chem. Phys.* **1993**, *98*, 5648-5652.

S23. Mennucci, B. *Wiley Interdisciplinary Reviews: Computational Molecular Science* **2012**, *2*, 386-404.

S24. Tomasi, J.; Mennucci, B.; Cammi, R. *Chem. Rev.* **2005**, *105*, 2999-3094.

S25. Cornell, W. D.; Cieplak, P.; Bayly, C. I.; Gould, I. R.; Merz Jr., K. M.; Ferguson, D. M.; Spellmeyer, D. C.; Fox, T.; Caldwell, J. W.; Kollman, P. A. *J. Am. Chem. Soc.* **1995**, *117*, 5179-5197.

S26. Jorgensen, W. L.; Chandrasekhar, J.; Madura, J. D.; Impey, R. W.; Klein, M. L. *J. Chem. Phys.* **1983**, *79*, 926-935.

S27. Joung, I. S.; Cheatham III, T. E. *J. Phys. Chem. B* **2008**, *112*, 9020-9041.

S28. Ryckaert, J.-P.; Ciccotti, G.; Berendsen, H. J. C. *J. Comput. Phys.* **1977**, *23*, 327-341.

S29. Darden, T.; York, D.; Pedersen, L. *J. Chem. Phys.* **1993**, *98*, 10089-10092.

S30. Essmann, U.; Perera, L.; Berkowitz, M. L.; Darden, T.; Lee, H.; Pedersen, L. G. *J. Chem. Phys.* **1995**, *103*, 8577-8593.

S31. Roe, D. R.; Cheatham, T. E. *J. Chem. Theory Comput.* **2013**, *9*, 3084-3095.

S32. Humphrey, W.; Dalke, A.; Schulten, K. *J. Molec. Graphics* **1996**, *14*, 33-38.

S33. Stewart, J. J. P. "PM3". *Encyclopedia of Computational Chemistry. Wiley*, **1998**.

S34. Brooks, B.R.; Brooks, C.L.; Mackerell, A.D.; Nilsson, L.; Petrella, R.J.; Roux, B.; Won, Y.; Archontis, G.; Bartels, C.; Boresch, S.; et al. *J. Comput. Chem.* **2009**, *30*, 1545–1614.

S35. a) Kumar, S.; Rosenberg, J.M.; Bouzida, D.; Swendsen, R.H.; Kollman, P.A. *J. Comput. Chem.* **1992**, *13*, 1011–1021; b) Souaille, M.; Roux, B. *Comput. Phys. Commun.* **2001**, *135*, 40–57.

S36. Dijkstra, E.A. *Numer. Math.* **1959**, *1*, 269.

S37. Van Rossum, G.; Drake, F. L. Python 3 Reference Manual ; CreateSpace: Scotts Valley, CA, **2009**.

S38. Hunter, J. D. Matplotlib: A 2D graphics environment. *Comput. Sci. Eng*. **2007**, *9*, 90-95.

S39. Ingargiola, M.; Runge, R.; Heldt, J.M.; Freudenberg, R.; Steinbach, J.; Cordes, N.; Baumann, M.; Kotzerke, J.; Brockhoff, G.; Kunz-Schughart, L.A. *Int. J. Cancer* **2014**, *135(4)*, 968-980.

S40. Kunz-Schughart, L.A.; Schroeder, J.A.; Wondrak, M.; van Rey, F.; Lehle, K.; Hofstaedter, F.; Wheatley, D.N. *Am. J. Physiol. Cell Physiol.* **2006**, *290(5)*, C1385-98.

S41. Xu, H.-G.; Reshetnikov, V.; Wondrak, M.; Eckardt, L.; Kunz-Schughart, L. A.; Janko, C.; Tietze, R.; Alexiou, C.; Borchardt, H.; Aigner, A.; Gong, W.; Schmitt, M.; Sellner, L.; Daum, S.; Özkan, H. G.; Mokhir, A. *Cancers* **2022**, *14(1)*, 208.

S42. Chen, O.; Manig, F.; Lehmann, L.; Sorour, N.; Löck, S.; Yu, Z.; Dubrovska, A.; Baumann, M.; Kessler, B.M.; Stasyk, O.; Kunz-Schughart, L.A. *Cell. Mol. Life Sci.* **2021**, *78(6)*, 3021-3044.

S43. Özkan, H. G.; Thakor, V.; Xu, H.; Bila, G; Bilyy, R.; Bida, D.; Böttcher, M.; Mougiakakos, D.; Tietze, R.; Mokhir, A. *Chem. Eur. J.* **2022**, e202104420.
